# Synthesis of Perfluoroalkylated Pyrazoles from α-Perfluoroalkenylated Aldehydes

**DOI:** 10.3390/molecules29215034

**Published:** 2024-10-25

**Authors:** Lennart Bunnemann, Christian Wulkesch, Victoria Carina Voigt, Constantin Czekelius

**Affiliations:** Department of Organic Chemistry and Macromolecular Chemistry, Heinrich-Heine-Universität, 40225 Düsseldorf, Germanychristian.wulkesch@hhu.de (C.W.);

**Keywords:** pyrazoles, aldehydes, perfluoroalkylations, photocatalysis, heterocycles

## Abstract

Within this study, we report a simple two-step process for the synthesis of perfluoroalkylated pyrazoles from aliphatic aldehydes. In the photocatalytic first step, the aldehydes are transformed into the corresponding perfluoroalkylated enals, which then undergo nucleophilic attack by hydrazine and subsequent ring closure, providing the fluorinated 3,4-substituted pyrazole products in a 64–84% yield. Using triphenylphosphine and imidazolidinone as organocatalysts, the method is operationally simple and omits heavy metal-containing waste.

## 1. Introduction

Pyrazole derivatives are a very common structural motif in fine chemical synthesis and have found widespread application in pharmacy, agrochemistry, and material sciences. They show interesting characteristics as inhibitors of protein glycation as well as anti-bacterial, anti-cancer, anti-depressant, anti-fungal, anti-tuberculosis, and anti-viral activity [1,2]. Consequently, several well-established drugs are based on the pyrazole structural motif such as Celecoxib and Mavacoxib [3,4,5,6,7,8]. The incorporation of fluorine into organic molecules can profoundly alter their characteristics [9]. This modification is frequently employed to finetune pharmacodynamics and pharmacokinetics associated with pKa, metabolic stability, and lipophilicity [10,11,12,13]. Therefore, easy synthetic access to fluorinated pyrazoles opens new avenues, as evidenced by the growing number of papers and patents dedicated to this class of heterocycles [14]. These rising numbers also correlate to the advent of really successful fungicides such as bixafen, benzovindiflupyr, fluxapyroxad, and others [15,16].

There are multiple ways to synthesize pyrazoles via (3+2) cyclocondensations or (3+2) cycloadditions, which have been addressed in several reviews [1,14]. A recent, comprehensive review by Müller and co-workers highlighted the advantages of multicomponent synthesis in this respect [17]. Different methods are established to fluorinate a pre-existing pyrazole with electrophilic fluorinating agents such as Selectfluor^®^ or perfluoroalkyl esters. For example, Yamakawa and co-workers reported the trifluoromethylation of this heterocycle in the presence of Fe(II) compounds [18,19]. However, such fluorination is often hampered by low regioselectivity, and therefore most of the syntheses focus on already fluorinated or perfluoroalkylated precursors, which undergo heterocyclization to form the corresponding pyrazoles [14]. Examples include the condensation of perfluoroalkylated 1,3-dicarbonyl compounds [20] and the [3+2] cycloaddition reaction of fluorinated ene components with diazo compounds [21,22].

**Scheme 1:**
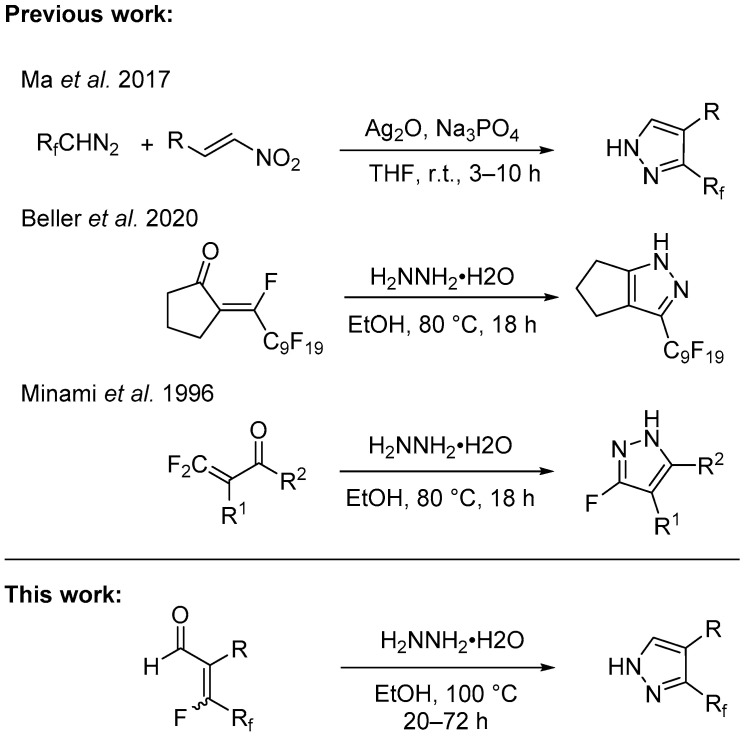
Methods for the synthesis of perfluoroalkylated pyrazoles [23,24,25].

For the synthesis of perfluoroalkylated 3,4-disubstituted-1*H*-pyrazoles, there are very few methods reported in the literature so far. Previous work by Ma and co-workers demonstrated the use of nitroolefins and trifluorodiazoethane in the presence of silver oxide and sodium phosphate [23]. This reaction shows a wide substrate scope and good yields but employs hazardous intermediates. The research groups of Beller and Minami independently showed that the condensation of fluorinated/perfluorinated enons with hydrazine leads to fluorinated pyrazoles (Scheme 1) [24,25]. Both approaches are limited to fluorinated enones, however, and only pyrazoles carrying a substituent in 5-position are accessible this way.

**Scheme 2:**
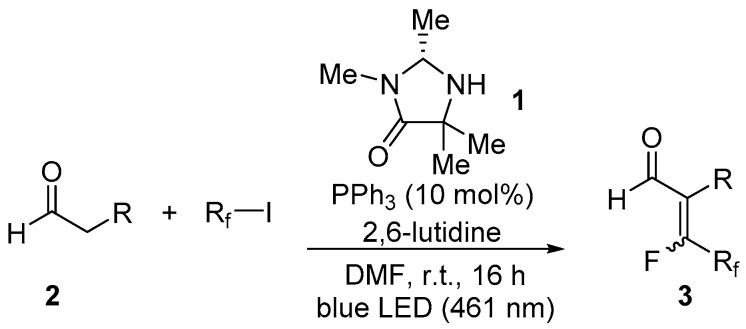
Photocatalytic synthesis of perfluoroalkylenals.

In 2021, we reported an operationally simple way to synthesize α-perfluoroalkenylated aldehydes under mild photocatalytic conditions [26]. A coupled catalyst system is used for this purpose (Scheme 2). Perfluoroalkyl iodides are activated by the phosphine photocatalyst weakening the C-I bond, which is then prone to cleavage under blue light irradiation [27]. The perfluoroalkyl radical formed this way presumably attacks the enamine intermediately formed from the imidazolidinone and the aldehyde. Iminium hydrolysis and HF-elimination render the electron-deficient enal.

In this contribution, the highly electron-deficient fluorinated enals are used as intermediates for the synthesis of pyrazoles in an operationally simple way. A double nucleophilic attack of hydrazine at the aldehyde and the conjugate position renders the hetero-aromatic, five-membered ring with high functional group tolerance (Scheme 1).

## 2. Results and Discussion

As a precursor for the evaluation of the method, *E*/*Z*-2-(perfluorobutylidene)-octanal (**3a**) was synthesized from octanal (**2a**) and perfluorobutyl iodide using (*S*)-2,2,3,5-tetra-methylimidazolidin-4-one (**1**), triphenylphosphine, and 2,6-lutidine following our previously reported procedure [26]. Beller and coworkers showed that enones provide access to perfluoroalkylated pyrazoles using hydrazine monohydrate (1.25 equiv) in ethanol upon heating to reflux for 18 h [25]. Employing these conditions, we investigated the corresponding cyclization of β-fluoro-β-perfluoroalkylated enal **3a**. Only two new sets of signals were detected by ^1^H NMR, corresponding to the (2-(perfluorobutylidene)octylidene)hydrazone (**4a′**) but only minor amounts of the desired product 4-hexyl-3-perfluoropropyl-1*H*-pyrazole (**4a**) (Table 1). No residual signals of starting material **3a** were visible. For method optimization, the conversion of the starting material under different reaction conditions was therefore monitored using the signal of the CF_3_-groups in ^19^F-NMR of hydrazone **4a′** and pyrazole **4a** in the crude reaction mixture.

Raising the temperature to 100 °C and using superstoichiometric amounts of hydrazine monohydrate led to a significant increase in yield (Entry 2 and 3) compared to Beller’s conditions. When the reaction mixture was heated for elongated periods of time (11 days), a conversion of 96% was observed (Entry 4). Testing DMF as a solvent for a potential one-pot transformation led to a significant number of unidentified byproducts, as found by many new signals in the ^19^F NMR spectrum. The transformation is technically straightforward and no exclusion of air is necessary. For a simple and convenient reaction setup, a sealed pressure tube was employed.

**Scheme 3:**
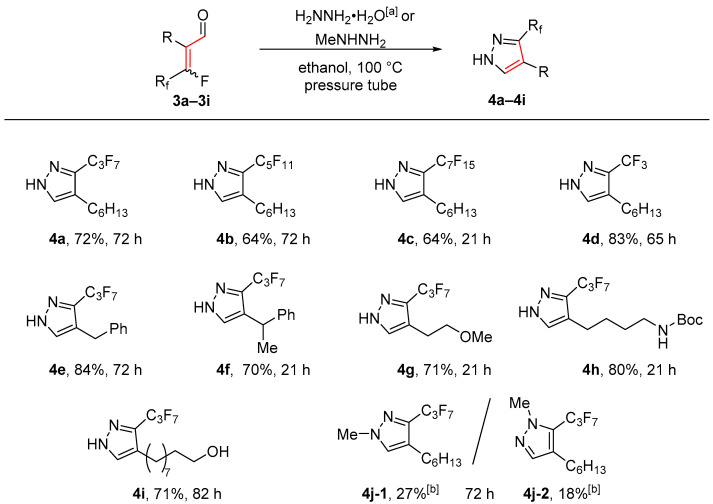
Synthesis of 3-(perfluoroalkylated)-1*H*-pyrazoles; [a] reaction conditions: **3** (0.5 mmol), hydrazine monohydrate (1.85 mmol), in 2 mL ethanol at 100 °C under nitrogen. Isolated yields only. [b] Reduced yield due to high volatility of the product at reduced pressure.

As illustrated in Scheme 3, a variety of enals were successfully transformed into the corresponding pyrazoles (**4a**–**4i**) in a 64–84% yield by condensation with hydrazine hydrate. Varying the perfluoroalkyl chain length (compounds **4a**–**4d**) shows that with a decreasing chain length, higher yields can be achieved. Different functional groups are suitable without any decrease in yield, such as alcohols (**4i**), ethers (**4g**), protected amines (**4h**), or benzyl groups (**4e**). It could also be shown that the sterically more hindered, branched β-methylated enal **3f** does not suffer from a decrease in yield in the reaction to the corresponding pyrazole **4f**.

To clarify whether 3,4- or 4,5-substituted pyrazoles are predominantly formed, a NOESY-NMR experiment of compound **4d** was conducted (see Supplementary Information). Coupling between the aromatic proton and the proton on the nitrogen atom was found, indicating that, indeed, the 3,4-substituted tautomer is the dominant one. This is in line with the nearly identical shift values in the NMR spectra of 4-butyl-3-(trifluoromethyl)-1*H*-pyrazole that was reported by Ma and coworkers [23]. Calculations suggest that an electron-withdrawing group in the 3-position of the pyrazole ring leads to a more stable tautomer [28].

Unprotected amines were not investigated since the perfluoroalkenylation of aldehydes is incompatible with such a functional group. A synthetic pathway to 3-fluoro-pyrazoles would be desirable by the analogous transformation of an α-(trifluromethyl)-aldehyde. Unfortunately, these intermediates do not undergo efficient elimination to the corresponding difluoromethylidenes and are therefore synthetically not easily accessible.

Not surprisingly, the synthesis of pyrazoles bearing a good leaving group in the aldehyde side chain, such as bromine or iodine, is not feasible since hydrazine is a strong nucleophile leading to fast nucleophilic substitution as a competing process. Further modification of the pyrazoles is also possible using substituted hydrazines. This was exemplified by using methylhydrazine. Both regioisomers, hexyl-1-methyl-3-(perfluoropropyl)-1*H*-pyrazol (**4j-1**) and 4-hexyl-1-methyl-5-(perfluoropropyl)-1*H*-pyrazol (**4j-2**), were isolated in a 2:1 ratio. The reduced combined yield in this transformation can be explained by the volatility of the product at low pressure.

**Scheme 4:**
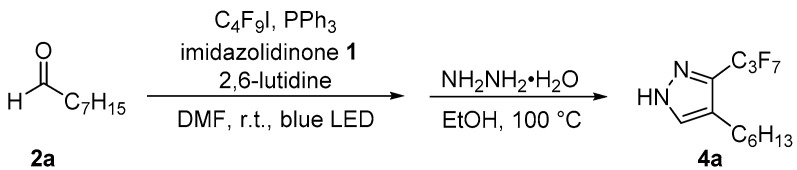
One-pot transformation of *n*-octanal (**2a**) to pyrazole **4a**. Reaction conditions: **2a** (0.53 mmol), **1** (0.1 equiv), PPh_3_ (0.1 equiv), 2,6-lutidine (1.5 equiv), nonafluoro-1-iodobutane (1.9 equiv), in 0.33 mL DMF at r.t., 20 h under irradiation of blue light (461 nm). Then, addition of hydrazine hydrate (3.5 equiv) in 1.5 mL ethanol and heating to 100 °C for 21 h.

A one-pot transformation was also conducted using the standard conditions for both reactions using *n*-octanal (**2a**) as a model substrate (Scheme 4). First, the photoreaction was done in the typical way and afterwards, the whole reaction mixture was diluted with ethanol to give the same concentration of hydrazine hydrate and perfluoroalkenylated aldehyde in the solution as in the described cyclization reaction. This gave a yield of 11% of 4-hexyl-3-perfluoropropyl-1*H*-pyrazol (**4a**), which should be compared to the combined yield of 45% if the reaction is completed in two isolated steps using different solvents. A possible explanation for this observation could be the presence of DMF, which is an unfitting solvent for this type of cyclization, as found before (Table 1, entry 6).

**Scheme 5:**
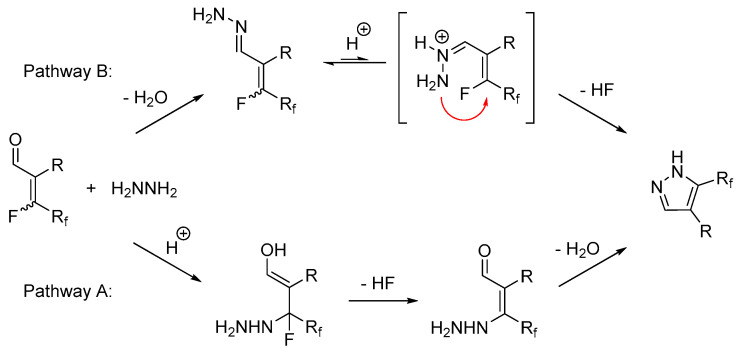
Proposed mechanism for the cyclization of the enal to the pyrazole.

The proposed reaction mechanism of the described cyclization is depicted in Scheme 5. In principle, there are two possible pathways. A direct substitution of the fluorine atom (pathway A) has been proposed by Minami and coworkers for the related functionalization of enones [24]. In our case, we believe that hydrazine first attacks the carbonyl group to form the corresponding hydrazone by the elimination of water (pathway B). This is feasible, since the aldehyde moiety is more accessible than the sterically congested 4-position. In fact, it is possible to isolate the hydrazones in substantial amounts if the reaction is not allowed to go to completion. However, for the cyclization of the hydrazone to the pyrazoles, *E*/*Z*-isomerization of both the C=C and the C=N bond to the all-cis isomer is presumably necessary to allow for efficient ring closure. Its formation under equilibrium conditions is presumably mediated by nucleophilic catalysis, i.e., by hydrazine leading to full consumption of the starting material. Related to the results by the groups of Minami and Bouillon, an excess of hydrazine is needed, which could be explained by the capture of hydrogen fluoride formed in the aromatization process [24,29].

## 3. Experimental Section

### 3.1. General Information

All reactions were performed in oven-dried glassware equipped with a magnetic stirring bar and heating was performed by an oil bath if necessary. All commercially available chemicals were purchased from Sigma Aldrich (Saint Louis, MO, USA), Fluka (Buchs, Switzerland), VWR (Radnor, PA, USA), TCI (Tokyo, Japan), Merck (Darmstadt, Germany), Macherey-Nagel (Düren, Germany), Acros Organics (Waltham, MA, USA), Fluorochem (Hadfield, UK), Honeywell (Acton, MA, USA), and AppliChem (Darmstadt, Germany). Not commercially available aldehydes were prepared by literature-known procedures and were used immediately. The solvents *n*-hexane, ethyl acetate, *n*-pentane, acetone, and dichloromethane were distilled from the technical grade solvents. Other solvents were purchased from the companies mentioned above in p.a. grade, degassed if necessary, and dried over molecular sieve (4 Å). Further solvents were dried using the solvent purification system MP-SPS 800 from M.Braun (Garching, Germany).

Nonafluoro-1-iodobutane was purchased from TCI (Tokyo, Japan) and filtered through an oven-dried plug packed with aluminum oxide 90 basic 0.063–0.200 mm (Brockmann I) and molecular sieves (4 Å). The received colorless liquid was stored in a brown glass bottle.

All photoreactions were performed in a self-built, air-cooled photoreactor equipped with RGB LED strips (120 LEDs/m) [30]. The emission maximum was set to 461 nm. The glass vessels by Avantor (Radnor, PA, USA) were made of borosilicate glass and airtightly sealed by a Teflon-lined screw cap. Thin layer chromatography (TLC) was performed using precoated TLC sheets ALUGRAM Xtra SIL G/UV_254_ from Macherey-Nagel (Düren, Germany).

All ^1^H-, ^13^C-, COSY-, NOESY-, and ^19^F-NMR-spectra were recorded on a Bruker Advance III 300 (300 MHz) or Bruker Advance III 600 (600 MHz) at room temperature. Chemical shifts are reported in parts per million (ppm) with respect to tetramethylsilane. The spectra were calibrated to the residual solvent signal. The order of citation in parentheses is (a) multiplicity (s = singlet, d = doublet, t = triplet, q = quartet, p = pentet, m = multiplet, and combinations of those), (b) coupling constants (in Hertz), (c) number of protons or fluorine atoms. In the case of diastereomer mixtures, the number of protons or fluorine atoms is given in decimals referring to their ratio. All diastereomeric ratios were determined by the integration of distinguishable signals in the ^1^H-NMR spectra.

IR spectra were measured on a Jasco (Tokyo, Japan) FT/IR-6200 spectrometer as a thin film on a NaCl single crystal.

High-resolution mass spectra (HRMS) were measured with a Bruker Daltonics UHR-QTOF maXis 4G.

### 3.2. Synthesis of Starting Materials

The syntheses of the aldehydes and the perfluoroalkenylations were carried out according to the methodology developed earlier by our group [26].

General procedure I for the photocatalytic perfluoroalkenylation of aldehydes

In a 2.5 mL reaction vessel with a stirring bar, (*S*)-2,2,3,5-tetramethylimidazolidin-4-one (**1a**) and triphenylphosphine were dissolved in dimethylformamide. The corresponding perfluoroiodoalkane, aldehyde, and 2,6-lutidine were added and the reaction mixture was irradiated for 20 h using blue LEDs at r.t. while stirring. After dilution with diethyl ether (10 mL), the organic phase was washed with aqueous HCl solution (0.1 M, 10 mL). After separation, the aqueous phase was extracted with diethyl ether (5× 10 mL). The combined organic phases were dried and the solvent was removed at 50 °C without vacuum. The product was dried by slow evaporation overnight.

*2-(perfluoroethylidene)-octanal* (**3d**). Following general procedure I, octanal (**2a**) (0.128 g, 1.00 mmol, 1.00 equiv), (*S*)-2,2,3,5-tetramethylimidazolidin-4-on (**1a**) (16.4 mg, 0.115 mmol 0.15 equiv), triphenylphosphine (26.2 mg, 0.100 mmol, 0.10 equiv), pentafluoro-1-iodoethane (0.17 mL, 1.4 mmol, 1.4 equiv), and 2,6-lutidine (0.14 mL, 1.2 mmol, 1.2 equiv) were mixed with dimethylformamide (0.5 mL) and irritated for 21 h. Purification by flash chromatography (*n*-pentane/diethyl ether 95:5) gave a mixture of (*E*)- and (*Z*)-2-(perfluoroethylidene)-octanal (0.143 g, 0.632 mmol, 63%, *E*/*Z*; 60:40) as a colorless liquid.
^1^H NMR (300 MHz, CDCl_3_) δ = 10.19 (s, 0.4H), 10.06 (s, 0.6H), 2.43–2.27 (m, 2H), 1.43–1.24 (m, 8H), 0.88 (t, 3H). ^19^F NMR (282 MHz, CDCl_3_) δ = −63.52 (d, *J* = 7.8 Hz), −67.66 (d, *J* = 6.6 Hz), −110.52 (q, *J* = 7.9 Hz), −129.26 (q, *J* = 6.6 Hz). ^13^C NMR (75 MHz, CDCl_3_) δ 188.09–187.24 (m), 160.00–116.39 (m), 77.41, 76.98, 76.56, 34.11, 31.35, 31.28, 29.12, 28.99, 27.90, 22.70, 22.66, 22.44, 22.32, 21.86, 13.99, 13.92. IR: $\tilde{\nu }$ [cm^−1^] = 2960, 2933, 2863, 2367, 2341, 1698, 1669, 1335, 1204, 1156, 1094.
*2-(perfluorobutylidene)-3-methyl-3-phenylpropanal* (**3f**). Following general procedure I, 3-phenylbutyraldehyde (**2f**) (0.223 mL, 1.50 mmol, 1.00 equiv), (*S*)-2,2,3,5-tetramethylimidazolidin-4-on (**1a**) (27.4 mg, 0.193 mmol, 0.13 equiv), triphenylphosphine (47.5 mg, 0.181 mmol, 0.12 equiv), nonafluoro-1-iodobutane (0.535 mL, 3.11 mmol, 2.07 equiv), and 2,6-lutidine (0.23 mL, 2.0 mmol, 1.3 equiv) were mixed with dimethylformamide (1 mL) and irritated for 20 h. Purification by flash chromatography (*n*-pentane/diethyl ether 99.4:0.6) gave a mixture of (*E*)- and (*Z*)-2-(perfluorobutylidene)-3-methyl-3-phenylpropanal (0.090 g, 0.26 mmol, 17%, *E*/*Z*; 86:14) as a colorless liquid.
^1^H NMR (300 MHz, CDCl_3_) δ = 10.04 (d, *J* = 1.9 Hz, 0.1H), 9.94 (td, *J* = 1.7 Hz, 0.9, 0.9H), 7.37–7.27 (m, 3H), 7.27–7.17 (m, 2H), 4.46 (q, *J* = 7.3 Hz, 0.9H), 4.13 (d, *J* = 7.5 Hz, 0.1H), 1.70–1.58 (m, 3H). ^19^F NMR (282 MHz, CDCl_3_) δ = −80.27–−80.51 (m, 3F), −106.33 (m, 0.9F), −111.97 (m, 1.8F), −112.69–−112.80 (m, 0.01F), −113.62–−113.87 (m, 0.09F), −114.32–−114.51 (m, 0.08F), −115.44 (m, 0.01F), −122.97 (m, *z*0.03F), −126.81–−126.95 (m, 0.2F), −127.39 (dd, *J* = 6.6, 2.9 Hz, 1.7F). ^13^C NMR (151 MHz, CDCl_3_) δ = 187.65 (m), 187.44–187.17 (m), 156.55–155.77 (m), 154.62–154.04 (m) 141.02, 140.27, 137.32, 137.24, 135.45, 135.41, 133.81, 133.68, 128.68, 128.54, 128.49, 128.45, 128.43, 127.29, 127.18, 126.90, 126.82, 119.07–107.59 (m), 35.06, 34.14, 17.19, 17.16. IR: $\tilde{\nu }$ [cm^−1^] = 3059, 2930, 1698, 1654, 1435, 1354, 1265, 1232, 1189, 1127.

### 3.3. Synthesis of Perfluoroalkylated Pyrazoles

General procedure II for the cyclization of the α-perfluoroalkenylated aldehydes to the corresponding pyrazols (**4a**–**4j**)

All reactions were performed in an oven-dried pressure tube (20 mL) under a N_2_ atmosphere. The α-perfluoroalkenylated aldehydes (**3a**–**3i**) were added through a septum and dissolved in ethanol (2 mL). Hydrazine monohydrate was added and the septum was replaced by a screw plug. The reaction mixture was stirred at 100 °C. Subsequently, the solvent was removed and the crude product was purified by flash chromatography.

*4-hexyl-3-perfluoropropyl-1H-pyrazol* (**4a**). Following general procedure II, 2-(perfluorobutylidene)-octanal (**3a**) (0.169 g, 0.518 mmol, 1.00 equiv), hydrazine monohydrate (0.095 mL, 2.0 mmol, 3.9 equiv), and ethanol (2 mL) were mixed and stirred at 100 °C for 72 h. Purification by flash chromatography (*n*-pentane/diethyl ether 7:3) gave the desired product (120 mg, 0.373 mmol, 72%) as a colorless oil.
^1^H NMR (300 MHz, CDCl_3_) δ 13.19 (s, 1H), 7.47 (s, 1H), 2.57 (t, *J* = 7.8 Hz, 2H), 1.67–1.51 (m, 1H), 1.45–1.21 (m, 2H), 0.96–0.84 (m, 3H). ^19^F NMR (282 MHz, CDCl_3_) δ −80.25 (t, *J* = 9.6 Hz, 3F), −109.81 (q, *J* = 9.7 Hz, 2F), −126.75 (q, *J* = 7.4 Hz, 2F). ^13^C NMR (151 MHz, CDCl_3_) δ = 137.95 (t, *J* = 27.0 Hz.), 129.31, 122.85, 121.66–106.69 (m), 31.72, 30.55, 29.14, 23.35, 22.71, 14.11. IR: $\tilde{\nu }$ [cm^−1^] = 3177, 2962, 2935, 2863, 2360, 2341, 1347, 1230, 1212, 1116. HRMS-ESI: calcd for C_12_H_15_F_7_N_2_ [M + H^+^] *m*/*z* 321.1196, found 321.1197.; R_f_ = 0.3 in *n*-pentane/diethyl ether 7:3.
*4-hexyl-3-perfluoropentyl-1H-pyrazol* (**4b**). Following general procedure II, 2-(perfluorohexylidene)octanal (**3b**) (0.212 g, 0.498 mmol, 1.00 equiv), hydrazine monohydrate (0.090 mL, 1.9 mmol, 3.8 equiv), and ethanol (2 mL) were mixed and stirred at 100 °C for 72 h. Purification by flash chromatography (*n*-pentane/diethyl ether 7:3) gave the desired product (0.135 g, 0.321 mmol, 64%) as a colorless oil.
^1^H NMR (300 MHz, CDCl_3_) δ = 13.16 (s, 1H, H-1), 7.46 (s, 1H), 2.58 (t, *J* = 7.8 Hz, 2H), 1.67–1.51 (m, 2H), 1.45–1.21 (m, 6H,), 0.97–0.84 (m, 3H). ^19^F NMR (282 MHz, CDCl_3_) δ = −80.83 (tt, *J* = 10.0, 2.7 Hz, 3F), −108.77–−109.06 (m, 2F), −122.28–−122.50 (m, 2F), −122.49–−122.72 (m, 2F), −126.08–−126.34 (m, 2F). ^13^C NMR (151 MHz, CDCl_3_) δ = 138.24–137.73 (m), 129.21, 122.86, 120.85–105.82 (m), 30.45, 29.10, 23.31, 22.68, 14.12. IR: $\tilde{\nu }$ [cm^−1^] = 3177, 2962, 2934, 2863, 1469, 1361, 1239, 1205, 1145, 1106. HRMS-ESI: calcd for C_14_H_15_F_11_N_2_ [M + H^+^] *m*/*z* 421.1132, found 421.1133. R_f_ = 0.25 in *n*-pentane/diethyl ether 9:1.
*4-hexyl-3-perfluoroheptyl-1H-pyrazol* (**4c**). Following general procedure II, 2-(perfluorooctylidene)octanal (**3c**) (0.263 g, 0.500 mmol, 1.00 equiv), hydrazine monohydrate (0.090 mL, 1.9 mmol, 3.8 equiv), and ethanol (2 mL) were mixed and stirred at 100 °C for 21 h. Purification by flash chromatography (*n*-pentane/diethyl ether 7:3) gave the desired product (0.167 g, 0.322 mmol, 64%) as a colorless solid.
^1^H NMR (300 MHz, CDCl_3_) δ = 12.86 (s, 1H), 7.46 (d, *J* = 1.6 Hz, 1H), 2.57 (t, *J* = 7.8 Hz, 2H), 1.67–1.51 (m, 2H), 1.45–1.21 (m, 6H), 0.95–0.83 (m, 3H). ^19^F NMR (282 MHz, CDCl_3_) δ = −80.83 (tt, *J* = 9.9, 2.4 Hz, 3F), −108.74–−109.00 (m, 2F), −121.45–−121.77 (m, 2F), −121.95–−122.29 (m, 4F), −122.56–−122.87 (m, 2F), −126.15 (dddt, *J* = 19.3, 10.9, 7.3, 4.1 Hz, 2F). ^13^C NMR (151 MHz, CDCl_3_) δ = 137.77 (t, *J* = 27.5 Hz), 129.06, 122.68, 120.68–105.88 (m), 31.53, 30.26, 28.94, 23.14, 22.50, 13.90. IR: $\tilde{\nu }$ [cm^−1^] = 3178, 3116, 2962, 2936, 2865, 1471, 1366, 1241, 1210, 1150. HRMS-ESI: calcd for C_16_H_15_F_15_N_2_ [M + H^+^] *m*/*z* 521.1068, found 521.1063. Mp. 35–36 °C. R_f_ = 0.3 in *n*-pentane/diethyl ether 4:1.
*4-hexyl-3-(trifluoromethyl)-1H-pyrazole* (**4d**). Following general procedure II, 2-(perfluoroethylidene)octanal (**3d**) (0.104 g, 0.460 mmol, 1.00 equiv), hydrazine monohydrate (0.085 mL, 1.8 mmol, 3.9 equiv), and ethanol (1.9 mL) were mixed and stirred at 100 °C for 65 h. Purification by flash chromatography (*n*-pentane/diethyl ether 8:2) gave the desired product (0.084 g, 0.38 mmol, 83%) as a colorless oil.
^1^H NMR (300 MHz, CDCl_3_) δ = 13.62 (s, 1H), 7.49 (q, *J* = 1.0 Hz, 1H), 2.60 (t, *J* = 7.8 Hz, 2H), 1.67–1.56 (m, 2H), 1.41–1.27 (m, 6H), 0.96–0.86 (m, 3H). ^19^F NMR (282 MHz, CDCl_3_) δ = −60.77. ^13^C NMR (75 MHz, CDCl_3_) δ = 139.74 (q, *J* = 36.0 Hz), 129.24, 123.03 (q, *J* = 267.17), 120.66, 31.70, 30.52, 29.09, 23.17, 22.73, 14.18. IR: $\tilde{\nu }$ [cm^−1^] = 3177, 2959, 2861, 1490, 1377, 1281, 1158, 1130, 1062, 958. HRMS-ESI: calcd for C_10_H_15_F_3_N_2_ [M + H^+^] *m*/*z* 221.1260, found 221.1262. R_f_ = 0.25 in *n*-hexane/ethyl acetate 8:2.
*4-benzyl-3-perfluoropropyl-1H-pyrazol* (**4e**). Following general procedure II, 2-(perfluorobutylidene)-3-phenylpropanal (**3e**) (0.153 g, 0.461 mmol, 1.00 equiv), hydrazine monohydrate (0.085 mL, 1.7 mmol, 3.7 equiv), and ethanol (2 mL) were mixed and stirred at 100 °C for 72 h. Purification by flash chromatography (*n*-pentane/diethyl ether 8:2) gave the desired product (0.127 g, 0.388 mmol, 84%) as a colorless oil.
^1^H NMR (300 MHz, CDCl_3_) δ = 12.79 (s, 1H), 7.37–7.13 (m, 6H), 3.92 (s, 2H). ^19^F NMR (282 MHz, CDCl_3_) δ = −80.21 (t, *J* = 9.6 Hz, 3F), −109.80 (q, *J* = 9.7 Hz, 2F), −126.69 (d, *J* = 7.7 Hz, 2F). ^13^C NMR (151 MHz, CDCl_3_) δ = 139.61, 137.86 (t, *J* = 27.5 Hz), 130.57, 128.76, 128.70, 126.68, 121.79, 121.36–106.68 (m), 29.65. IR: $\tilde{\nu }$ [cm^−1^] = 3177, 3063, 2962, 1497, 1348, 1231, 1211, 1184, 1115, 1034. HRMS-ESI: calcd for C_13_H_9_F_7_N_2_ [M + H^+^] *m*/*z* 327.0727, found 327.0730. R_f_ = 0.3 in *n*-pentane/diethyl ether 4:1.
*3-(perfluorpropyl)-4-(1-phenylethyl)-1H-pyrazol* (**4f**). Following general procedure II, 2-(perfluorobutylidene)-3-methylen-3-phenylpropanal (**3f**) (0.103 g, 0.310 mmol, 1.00 equiv), hydrazine monohydrate (0.055 mL, 1.1 mmol, 3.5 equiv), and ethanol (1.4 mL) were mixed and stirred at 100 °C for 21 h. Purification by flash chromatography (*n*-pentane/diethyl ether 7:3) gave the desired product (73.8 mg, 0.217 mmol, 70%) as a colorless oil.
^1^H NMR (300 MHz, CDCl_3_) δ = 12.84 (s, 1H), 7.50 (d, *J* = 1.4 Hz, 1H), 7.34–7.16 (m, 5H), 4.26 (q, *J* = 7.2 Hz, 1H), 1.60 (d, *J* = 7.2 Hz, 3H). ^19^F NMR (282 MHz, CDCl_3_) δ = −80.17 (t, *J* = 9.7 Hz, 3F), −108.76–−109.12 (m, 2F), −126.47 (d, *J* = 6.6 Hz, 2F). ^13^C NMR (151 MHz, CDCl_3_) δ = 145.56, 137.30 (t, *J* = 27.6 Hz), 129.15, 128.61, 127.72, 127.04, 126.49, 121.70–106.26 (m), 34.62, 23.76. IR: $\tilde{\nu }$ [cm^−1^] = 3176, 2967, 1494, 1454, 1349, 1228, 1210, 1186, 1116, 1024. HRMS-ESI: calcd for C_14_H_11_F_7_N_2_ [M + H^+^] *m*/*z* 341.0883, found 341.0885.
*4-(2-methoxyethyl)-3-(perfluorpropyl)-1H-pyrazol* (**4g**). Following general procedure II, 2-(perfluorobutylidene)-4-methoxybutanal (**3g**) (0.164 g, 0.545 mmol, 1.00 equiv), hydrazine monohydrate (0.10 mL, 2.2 mmol, 4.0 equiv), and ethanol (2.2 mL) were mixed and stirred at 100 °C for 21 h. Purification by flash chromatography (*n*-pentane/diethyl ether 7:3) gave the desired product (0.114 g, 0.388 mmol, 71%) as a colorless oil.
^1^H NMR (300 MHz, CDCl_3_) δ = 13.04 (s, 1H), 7.63–7.56 (m, 1H), 3.57 (t, *J* = 6.5 Hz, 2H), 3.37 (s, 3H), 2.91–2.81 (m, 2H). ^19^F NMR (282 MHz, CDCl_3_) δ = −80.23 (t, *J* = 9.7 Hz, 3F), −109.82 (q, *J* = 9.7 Hz, 2.1F), −126.67–−126.88 (m, 2F). ^13^C NMR (151 MHz, CDCl_3_) δ = 137.93 (t, *J* = 27.2 Hz), 130.13, 118.82, 120.83–106.26 (m), 72.10, 58.50, 23.71. IR: $\tilde{\nu }$ [cm^−1^] = 3184, 2963, 2361, 2355, 2341, 1468, 1347, 1232, 1117, 1038. HRMS-ESI: calcd for C_9_H_9_F_7_N_2_ [M + H^+^] *m*/*z* 295.0676, found 295.0679. R_f_ = 0.16 in *n*-pentane/diethyl ether 4:1.
*tert-butyl (4-(3-(perfluorpropyl)-1H-pyrazol-4-yl)butyl)carbamate* (**4h**). Following general procedure II, *tert*-butyl-5-(perfluorobutylidene)-(6-oxohexyl)carbamate (**3h**) (0.201 g, 0.487 mmol, 1.00 equiv), hydrazine monohydrate (0.090 mL, 1.9 mmol, 3.9 equiv), and ethanol (2.5 mL) were mixed and stirred at 100 °C for 21 h. Purification by flash chromatography (*n*-pentane/diethyl ether 1:1) gave the desired product (0.158 g, 0.388 mmol, 80%) as a colorless oil.
^1^H NMR (300 MHz, CDCl_3_) δ = 12.86 (s, 1H), 7.46 (d, *J* = 1.6 Hz, 1H), 4.57 (s, 1H), 3.15 (q, *J* = 6.4 Hz, 2H), 2.58 (t, *J* = 7.3 Hz, 2H), 1.57 (dddd, *J* = 16.8, 15.0, 8.3, 3.4 Hz, 3H), 1.44 (s, 10H). ^19^F NMR (282 MHz, CDCl_3_) δ = −80.21 (t, *J* = 9.6 Hz, 3F), −109.67 (p, *J* = 8.5 Hz, 2F), −126.75 (d, *J* = 7.5 Hz, 2F). ^13^C NMR (151 MHz, CDCl_3_) δ = 156.24, 137.91 (t, *J* = 51.08), 129.21, 122.09, 121.62–106.15 (m), 79.38, 40.42, 29.92, 28.51, 27.83, 23.02. IR: $\tilde{\nu }$ [cm^−1^] = 3302, 3190, 2943, 1692, 1519, 1457, 1368, 1228, 1180, 1115. HRMS-ESI: calcd for C_15_H_20_F_7_N_3_NaO_2_ [M + Na^+^] *m*/*z* 430.1336, found 430.1135. R_f_ = 0.16 in *n*-pentane/diethyl ether 1:1.
*7-(3-(perfluorpropyl)-1H-pyrazol-4-yl)heptan-1-ol* (**4i**). Following general procedure II, 9-hydroxy-2-(perfluorobutylidene)nonanal (**3i**) (0.179 g, 0.504 mmol, 1.00 equiv), hydrazine monohydrate (0.090 mL, 1.9 mmol, 3.8 equiv), and ethanol (2 mL) were mixed and stirred at 100 °C for 82 h. Purification by flash chromatography (*n*-pentane/diethyl ether 3:7) gave the desired product (0.125 g, 0.357 mmol, 71%) as a colorless oil.
^1^H NMR (300 MHz, CDCl_3_) δ = 12.99 (s, 1H, H-1), 7.44 (s, 1H, H-2), 3.65 (t, *J* = 6.6 Hz, 2H, H-12), 2.55 (t, *J* = 7.8 Hz, 2H, H-6), 1.66–1.49 (m, 4H, CH2), 1.46–1.27 (m, 6H, CH2). ^19^F NMR (282 MHz, CDCl_3_) δ = −80.26 (t, *J* = 9.6 Hz, 3F), −109.75 (q, *J* = 9.7 Hz, 2.1F), −126.74–−126.81 (m, 2F). ^13^C NMR (151 MHz, CDCl_3_) δ = 137.75 (t, *J* = 27.5 Hz), 129.15, 122.61, 121.75–106.86 (m), 63.01, 32.75, 30.49, 29.37, 29.25, 25.78, 23.27. IR: $\tilde{\nu }$ [cm^−1^] = 3183, 2935, 2862, 1467, 1347, 1227, 1209, 1184, 1116, 1037. HRMS-ESI: calcd for C_13_H_17_F_7_N_2_O [M + H^+^] *m*/*z* 351.1302, found 351.1298. R_f_ = 0.1 in *n*-pentane/diethyl ether 3:7.
*4-hexyl-1-methyl-3(5)-(perfluoropropyl)-1H-pyrazol* (**4j**). General procedure II was employed, but hydrazine hydrate was exchanged by methylhydrazine. 2-(Perfluorobutylidene)octanal (**3a**) (0.169 g, 0.518 mmol, 1.00 equiv), methylhydrazine (0.098 mL, 1.9 mmol, 3.7 equiv), and ethanol (2 mL) were mixed and stirred at 100 °C for 72 h. Purification by flash chromatography (*n*-pentane/diethyl ether; 98:2, 95:5, 7:3) gave 4-hexyl-1-methyl-3-(perfluoropropyl)-1H-pyrazol (**4j-1**) (45 mg, 0.14 mmol, 27%) and 4-hexyl-1-methyl-5-(perfluoropropyl)-1H-pyrazol (**4j-2**) (30 mg, 0.090 mmol, 18%) as a slightly brown oil.
*4-Hexyl-1-methyl-3-(perfluoropropyl)-1H-pyrazol* (**4j-1**); ^1^H NMR (300 MHz, CDCl_3_) δ = 7.38 (d, *J =* 1.3 Hz, 1H), 3.94 (t, *J =* 1.8 Hz 8, 3H), 2.55–2.45 (m, 2H), 1.61–1.49 (m, 2H), 1.35–1.25 (m, 6H), 0.91–0.82 (m, 4H). ^19^F NMR (282 MHz, CDCl_3_) δ = −80.16 (t, *J =* 10.0 Hz, 3F), −107.68–−107.87 (m, 2F), −126.12–−126.28 (m, 2F). ^13^C NMR (75 MHz, CDCl_3_) δ = 138.86, 126.74, 125.64–125.18 (m), 39.50, 31.57, 30.49, 29.02, 23.67, 22.56, 22.33, 14.02. IR: $\tilde{\nu }$ [cm^−1^] = 2959, 2932, 2862, 1469, 1347, 1228, 1182, 1134, 1115, 1023. HRMS-ESI: calcd for C_13_H_18_F_7_N_2_ [M + H^+^] *m*/*z* 335.1353, found 335.1358. R_f_ = 0.8 in *n*-pentane/diethyl ether 9:1.
*4-Hexyl-1-methyl-5-(perfluoropropyl)-1H-pyrazol* (**4j-2**); ^1^H NMR (300 MHz, CDCl_3_) δ = 7.22 (s, 1H), 3.91 (s, 3H), 2.50 (t, *J =* 7.8 Hz, 2H), 1.57–1.46 (m, 2H), 1.37–1.22 (m, 6H), 0.92–0.85 (m, 3H). ^19^F NMR (282 MHz, CDCl_3_) δ = −80.21 (t, *J =* 9.7 Hz, 3F), −109.29 (q, *J =* 9.6 Hz, 2F), −126.56–−126.63 (m, 2F). ^13^C NMR (75 MHz, CDCl_3_) δ = 137.59 (t, *J =* 28.0 Hz), 130.38, 123.42, 120.72–104.88 (m), 39.48 (d, *J =* 3.1 Hz), 34.12, 31.58, 30.47, 29.00, 23.29, 22.57, 14.01. IR: $\tilde{\nu }$ [cm^−1^] = 2960, 2932, 2862, 1462, 1390, 1346, 1229, 1203, 1138, 1115. HRMS-ESI: calcd for C_13_H_18_F_7_N_2_ [M + H^+^] *m*/*z* 335.1353, found 335.1348. R_f_ = 0.4 in *n*-pentane/diethyl ether 9:1.

## 4. Conclusions

The experimental findings highlight the synthesis of highly functionalized fluorinated pyrazoles through a straightforward two-step process utilizing readily available aldehydes. While existing research primarily focused on alternative starting materials such as fluorinated or perfluoroalkylated enones, no prior synthesis from perfluoroalkylated enals had been documented. These are formed by a photocatalytic perfluoroalkenylation reaction that is followed by condensation with hydrazine hydrate at elevated temperatures. Ethanol as a polar, protic solvent was found most suitable. Larger amounts of hydrazine hydrate and higher temperatures promote conversion. As a compromise between superfluous reagent use and convenient reaction setup, the transformation proceeds smoothly at 100 °C in a pressure tube. The versatility of the method was shown by the successful transformation of substrates with a broad range of functional groups.

## Figures and Tables

**Table 1:** Screening of reaction conditions for the cyclization of 2-(perfluorobutylidene)-octanal (**3a**).

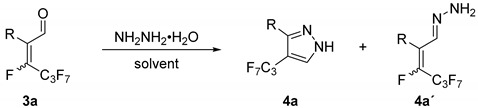					
Entry	Solvent	Hydrazine [Equiv]	Temperature [°C]	Time [h]	Conversion of 4a′ to 4a [%]
1	ethanol	1.3	80	21	31
2	ethanol	2.1	100	19	65
3	ethanol	4.9	100	21	77
4	ethanol	2.1	100	264	96
5	DMF	2.9	120	21	n.a.

General procedure: **3** (1.0 equiv) in ethanol (0.25 M), hydrazine monohydrate (3.7 equiv), at 100 °C.

## Data Availability

Data are contained within the article and Supplementary Materials.

## Supplementary Materials

The following supporting information can be downloaded at: https://www.mdpi.com/article/10.3390/molecules29215034/s1.
