# Supplementary material for: Synthesis of Perfluoroalkylated Pyrazoles from α-Perfluoroalkenylated Aldehydes
Source: Molecules. 2024 Oct 25;29(21):5034. doi: 10.3390/molecules29215034 (PMC11547949; doi:10.3390/molecules29215034)

Supporting Information

Synthesis of Perfluoroalkylated Pyrazoles from α-Perfluoroalkenylated Aldehydes

Lennart Bunnemann, Christian Wulkesch, Victoria Carina Voigt and Constantin Czekelius *

Department of Organic Chemistry and Macromolecular Chemistry,
Heinrich-Heine-Universität, 40225 Düsseldorf, Germany;
christian.wulkesch@hhu.de (C.W.)
* Correspondence: constantin.czekelius@hhu.de

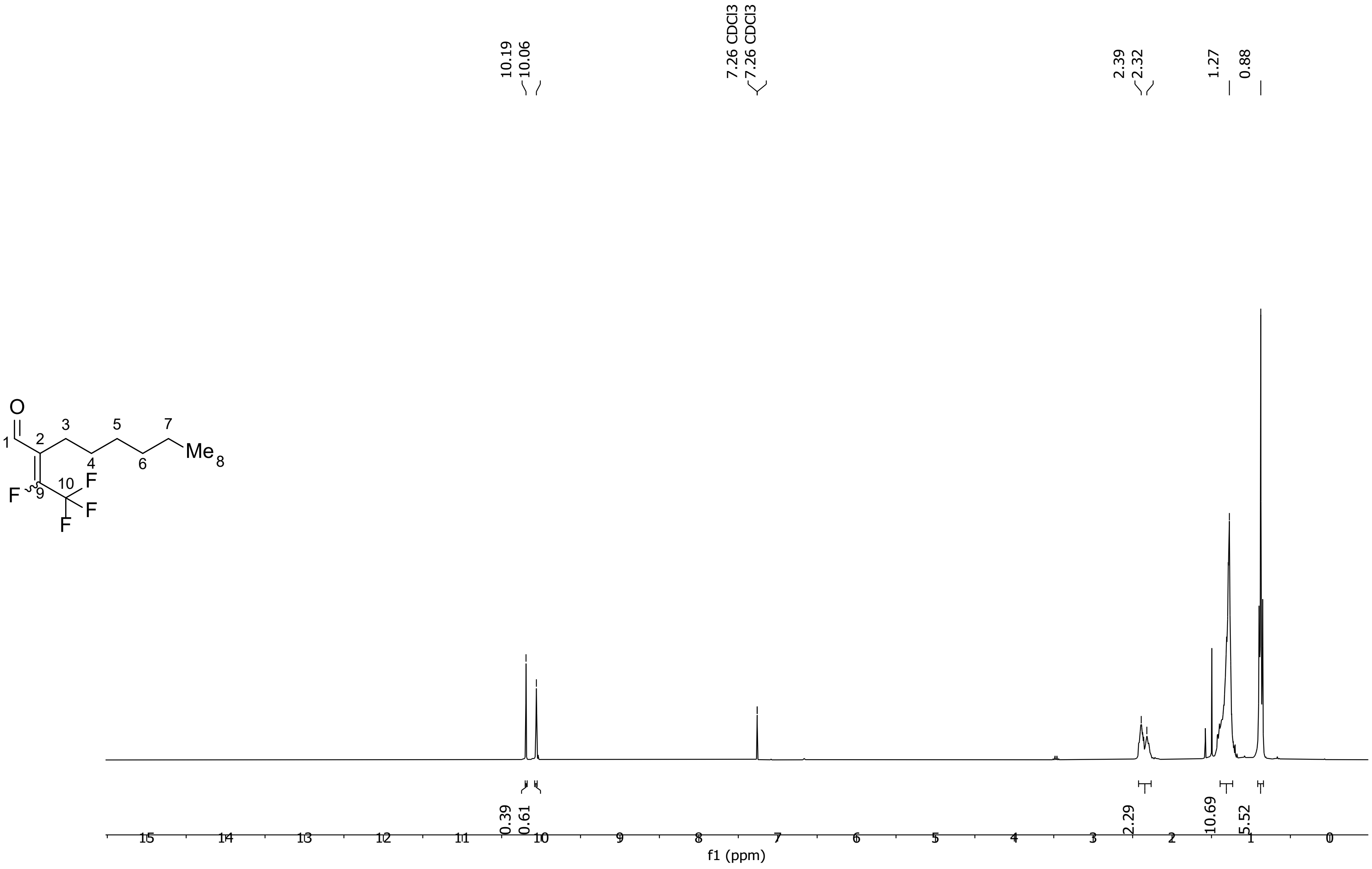

^{1}H NMR spectrum of compound **3d**

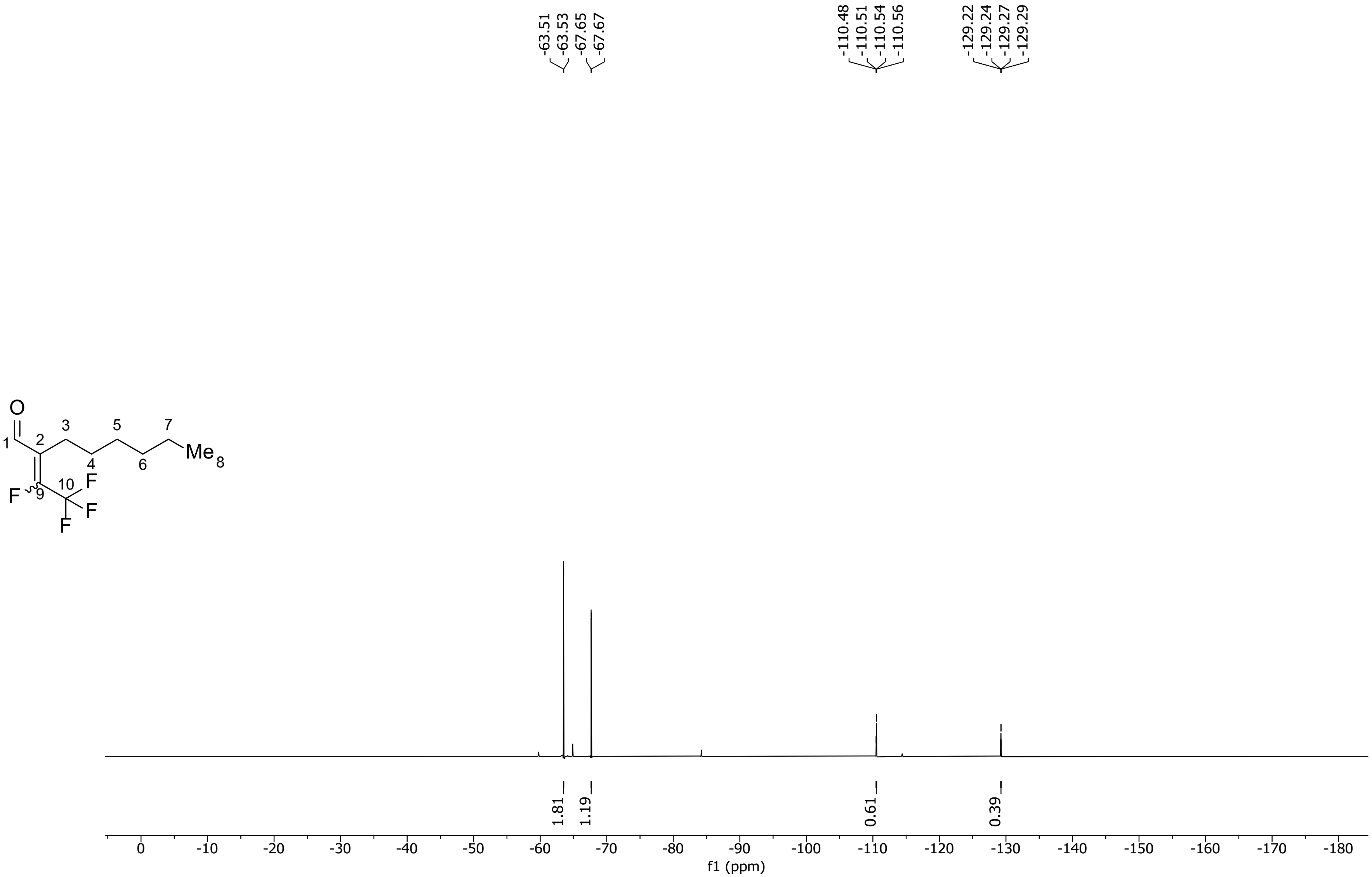

^{19}F NMR spectrum of compound **3d**

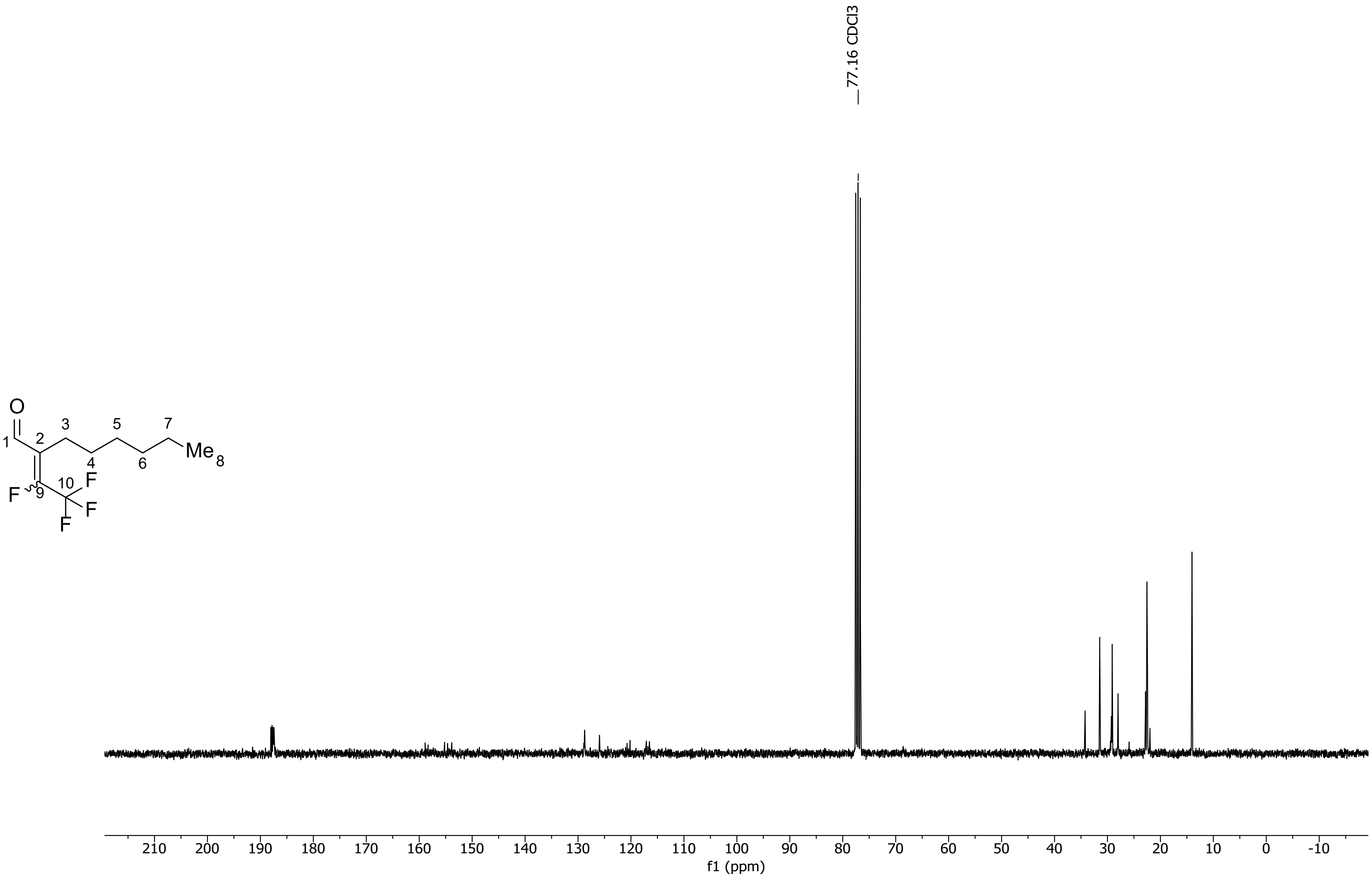

^{13}C NMR spectrum of compound **3d**

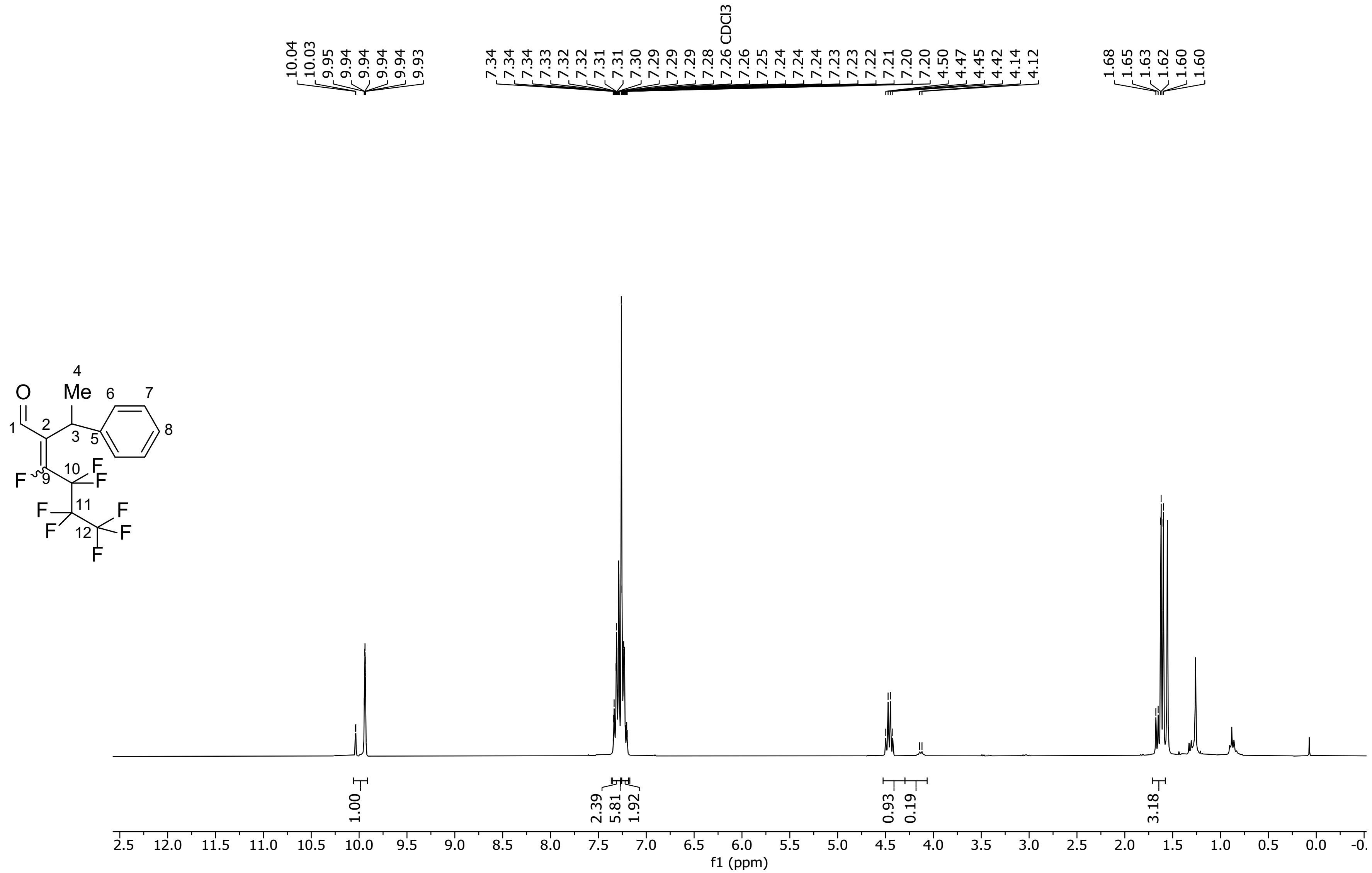

^{1}H NMR spectrum of compound **3f**

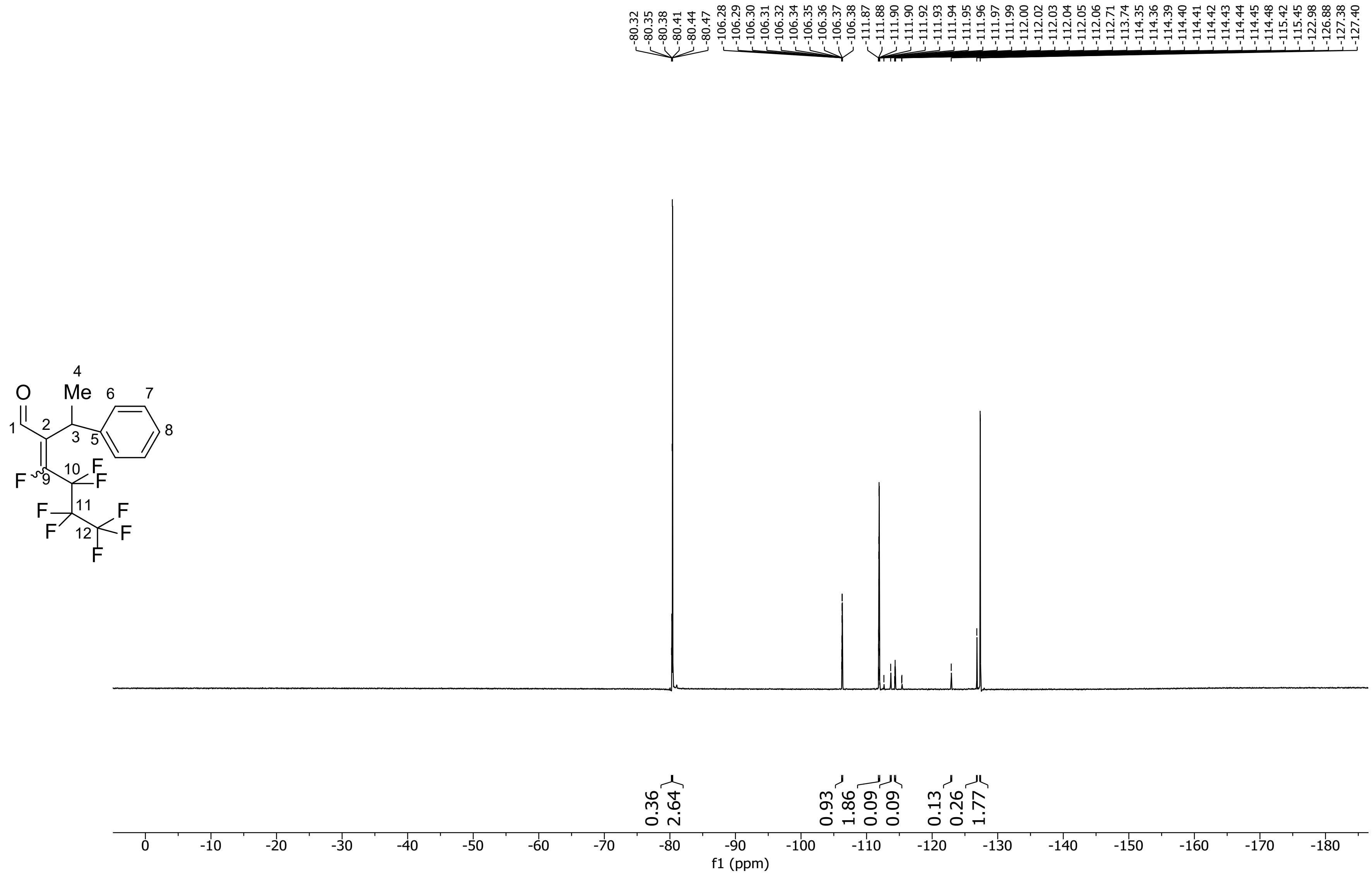

^{19}F NMR spectrum of compound **3f**

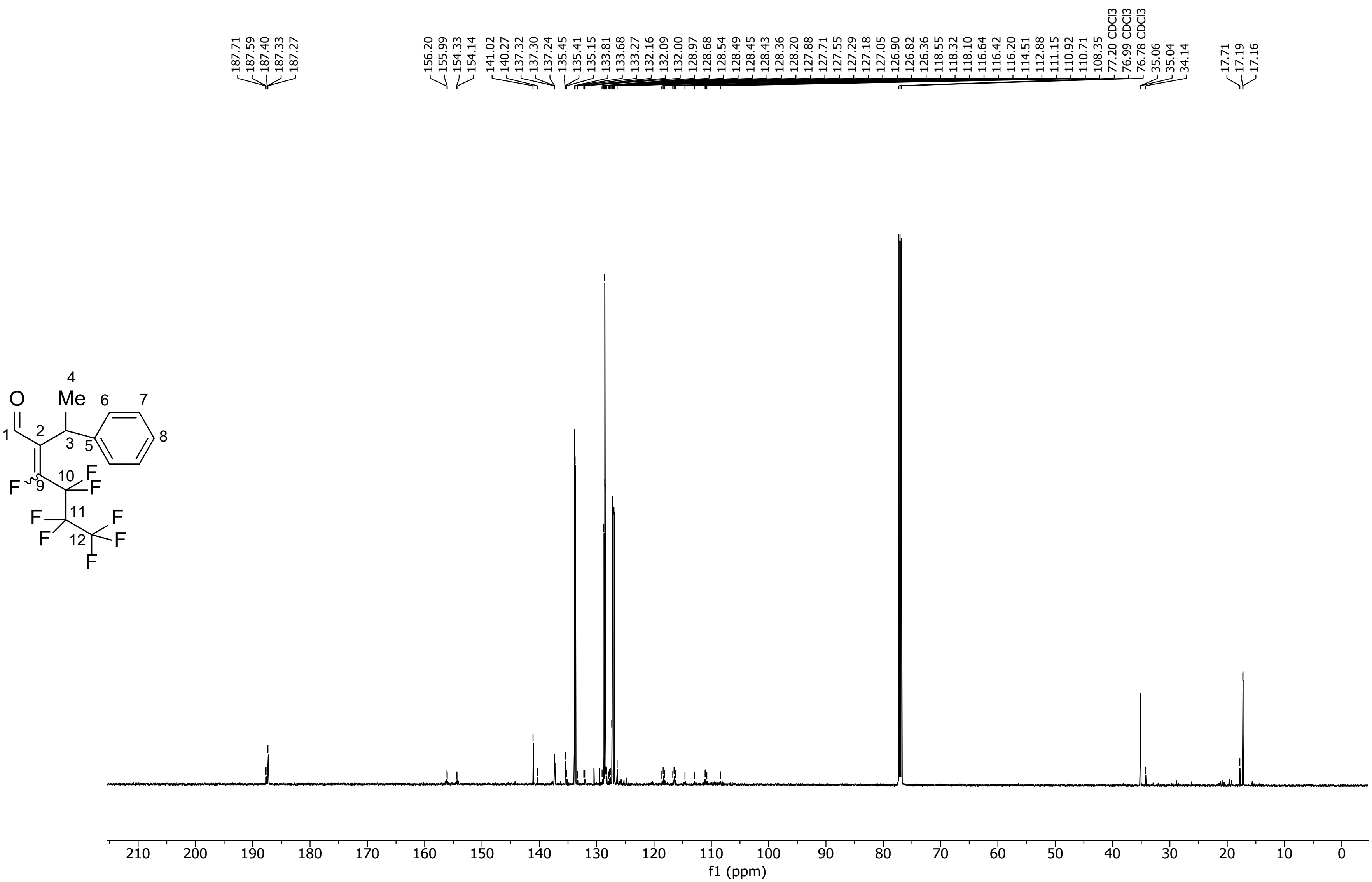

^{13}C NMR spectrum of compound **3f**

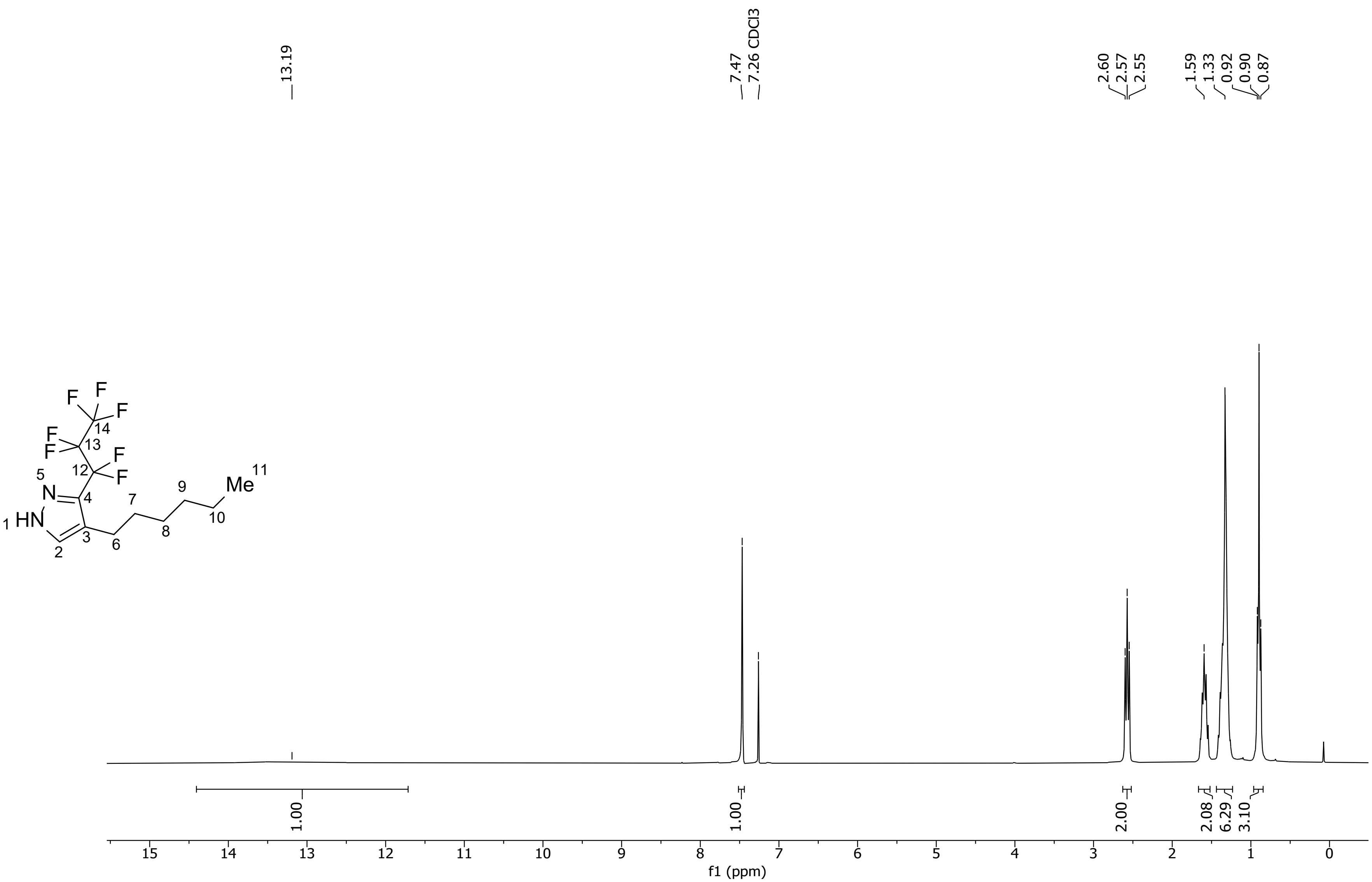

^{1}H NMR spectrum of compound **4a**

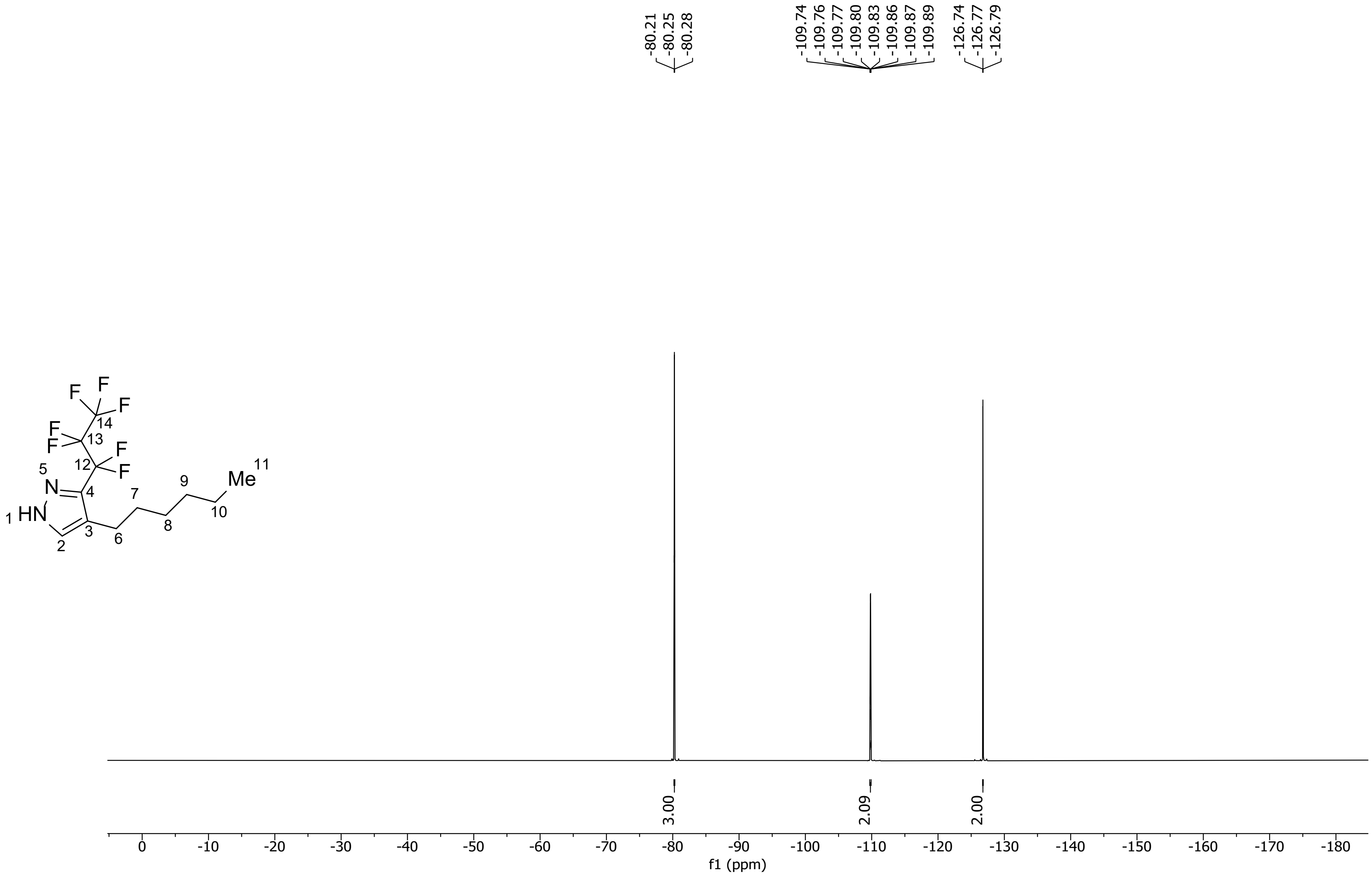

^{19}F NMR spectrum of compound **4a**

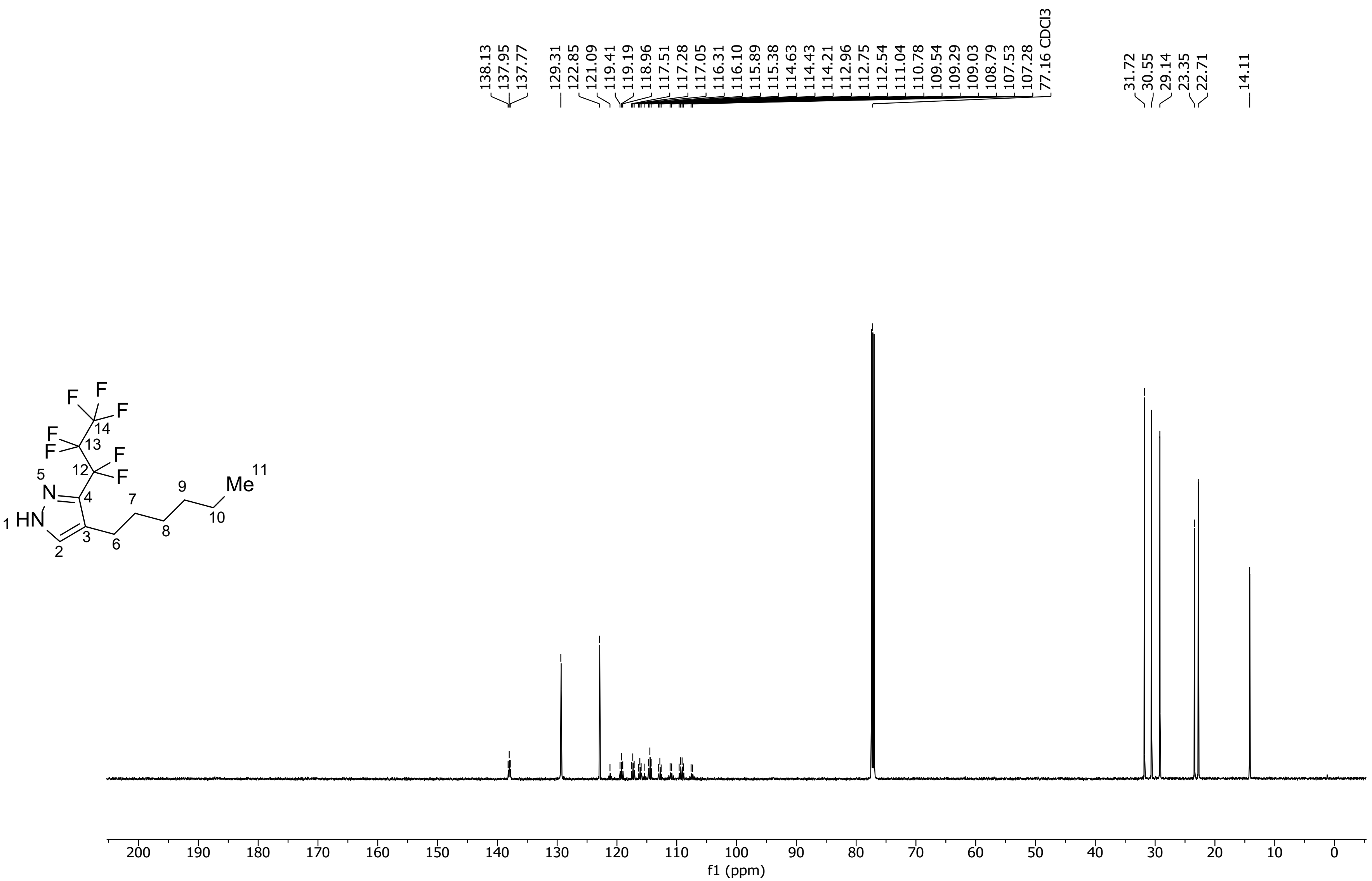

^{13}C NMR spectrum of compound **4a**

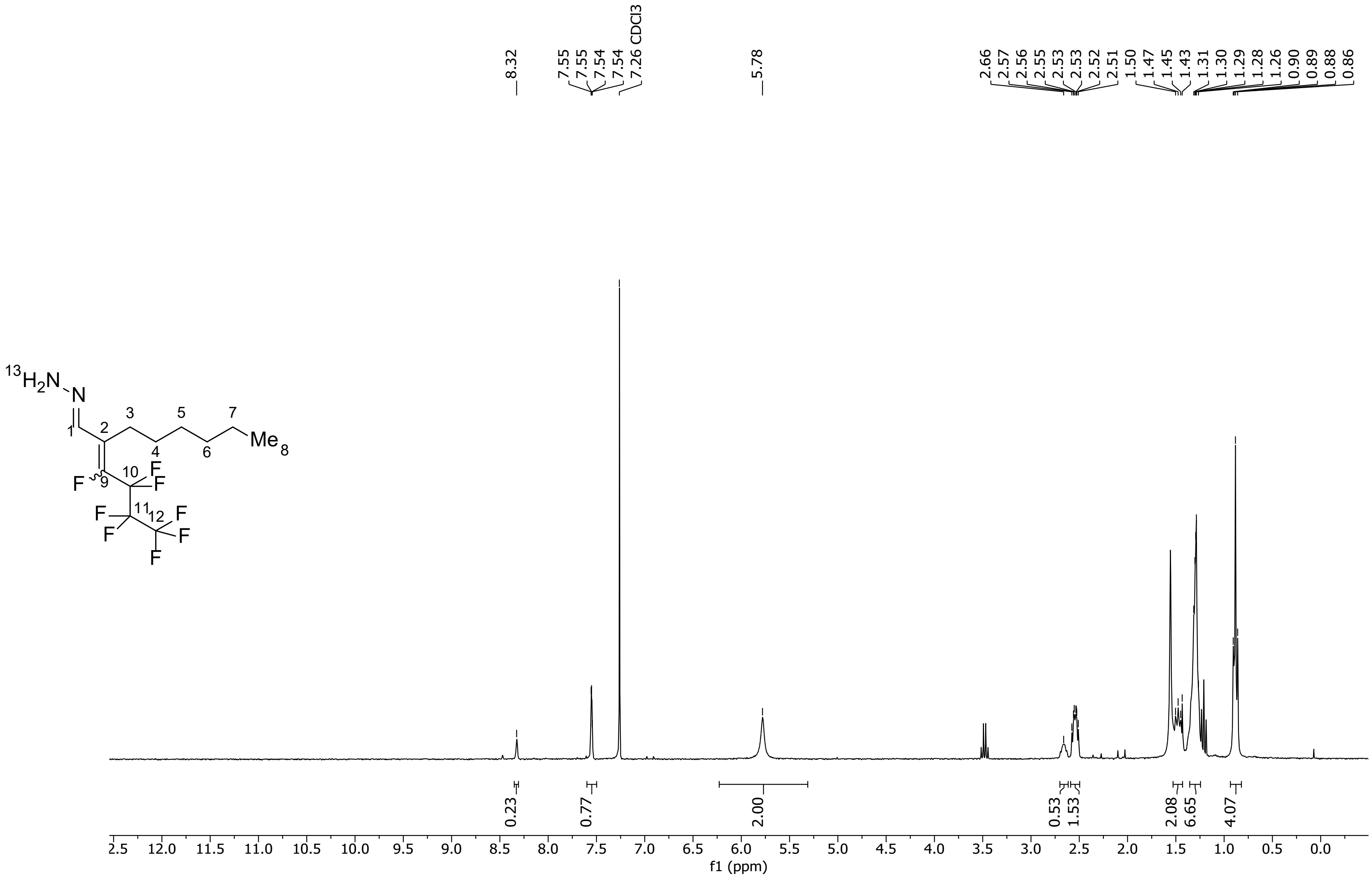

^{1}H NMR spectrum of compound **4a’**

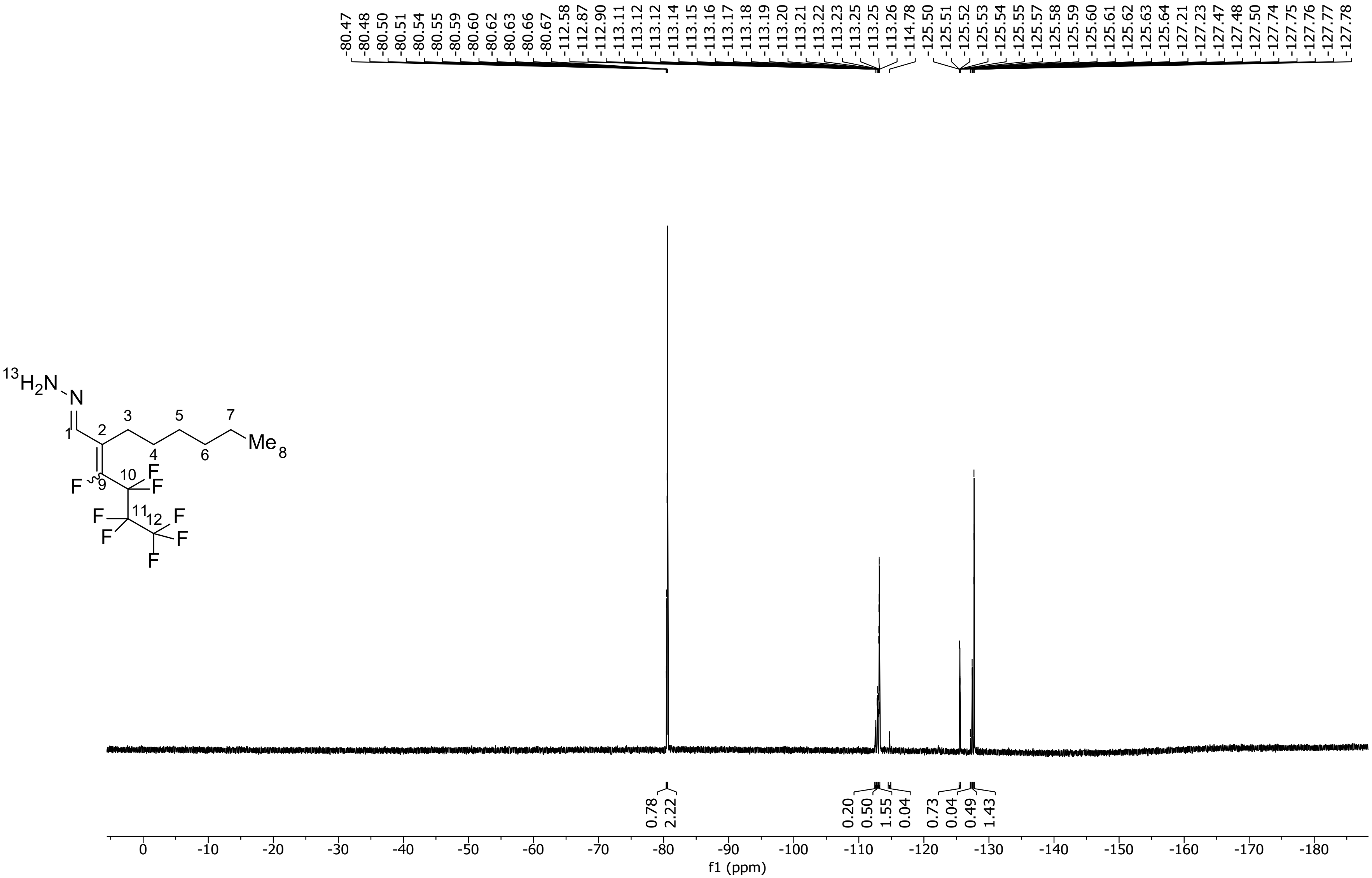

^{19}F NMR spectrum of compound **4a’**

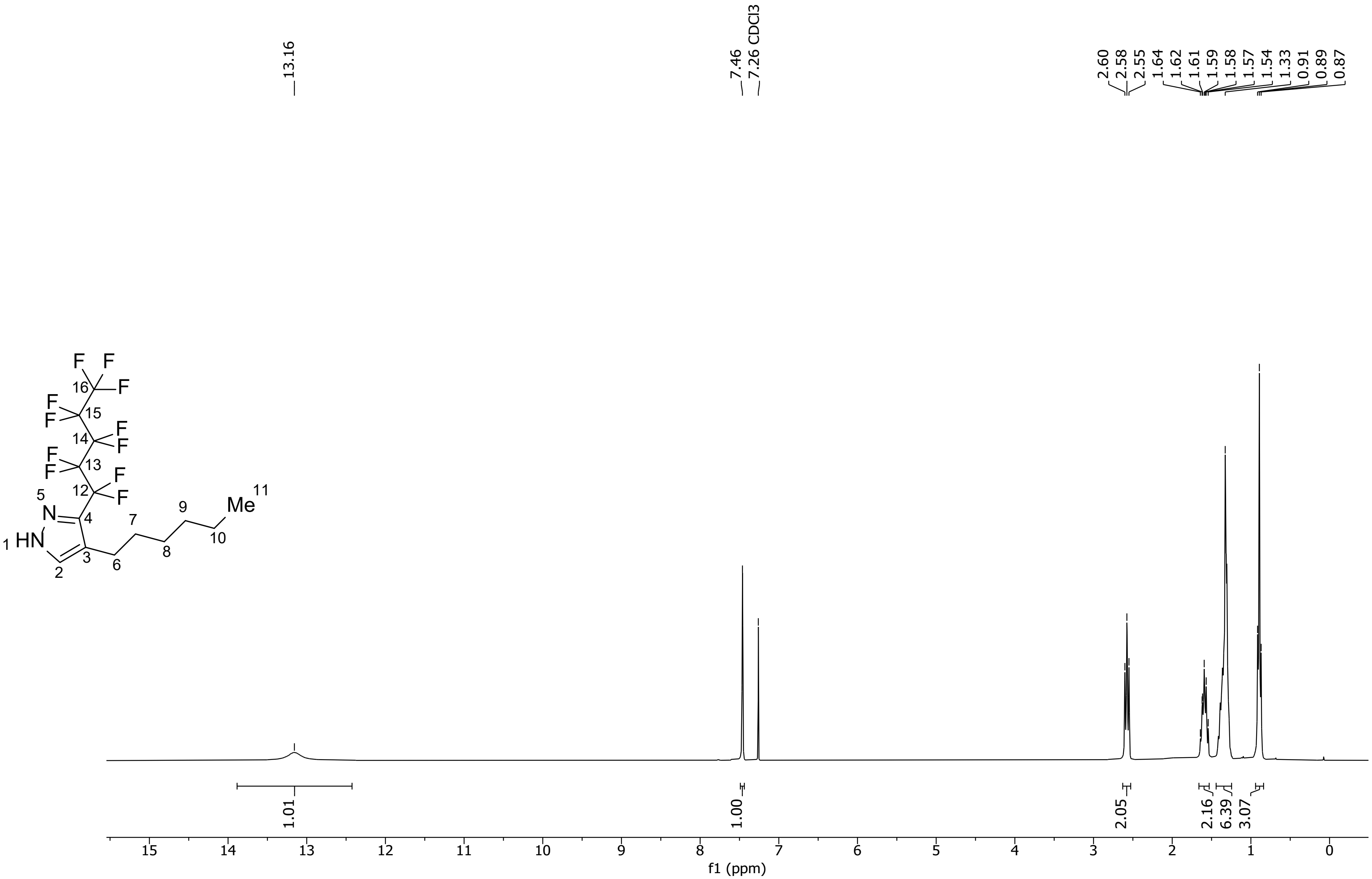

^{1}H NMR spectrum of compound **4b**

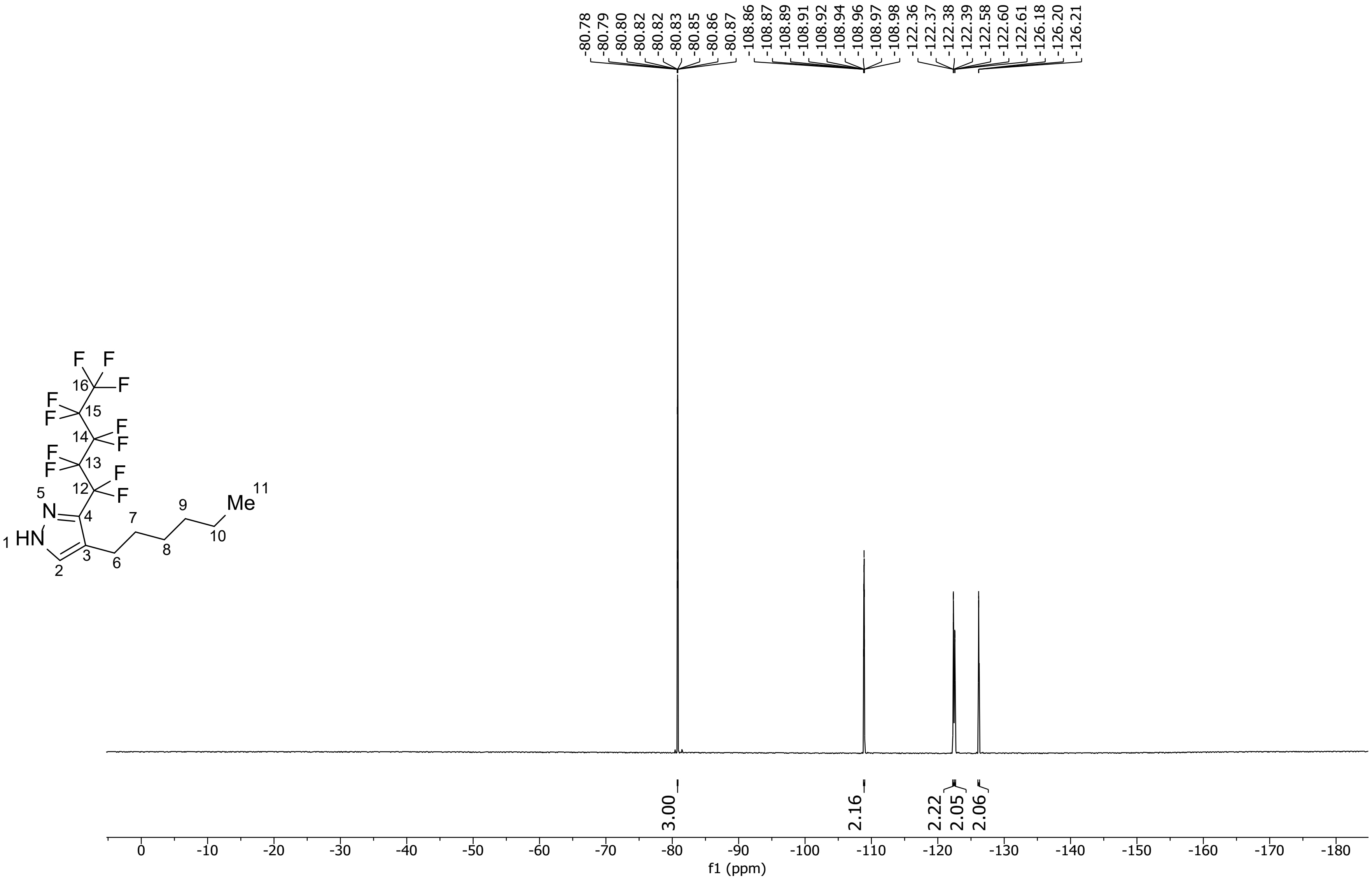

^{19}F NMR spectrum of compound **4b**

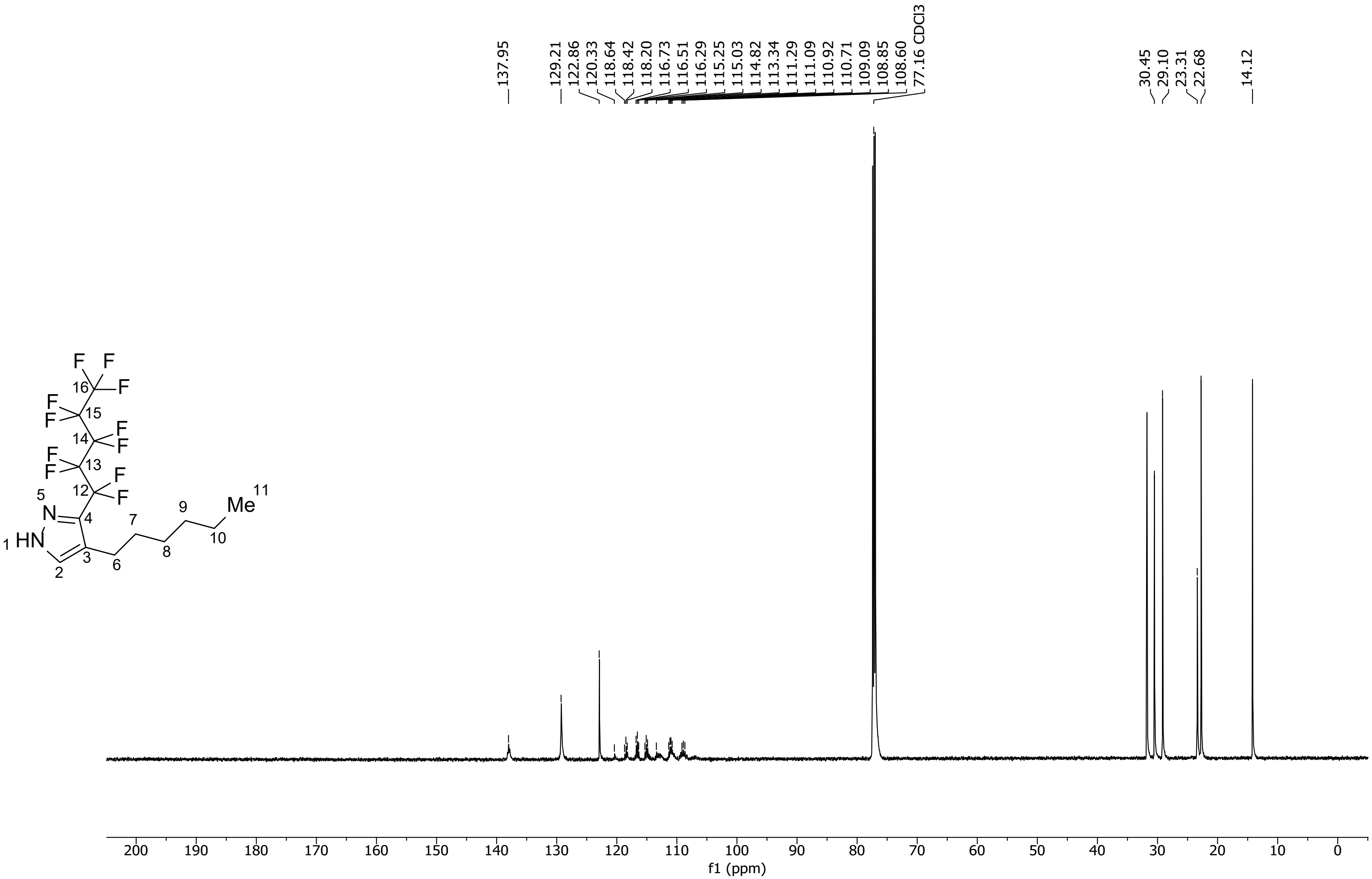

^{13}C NMR spectrum of compound **4b**

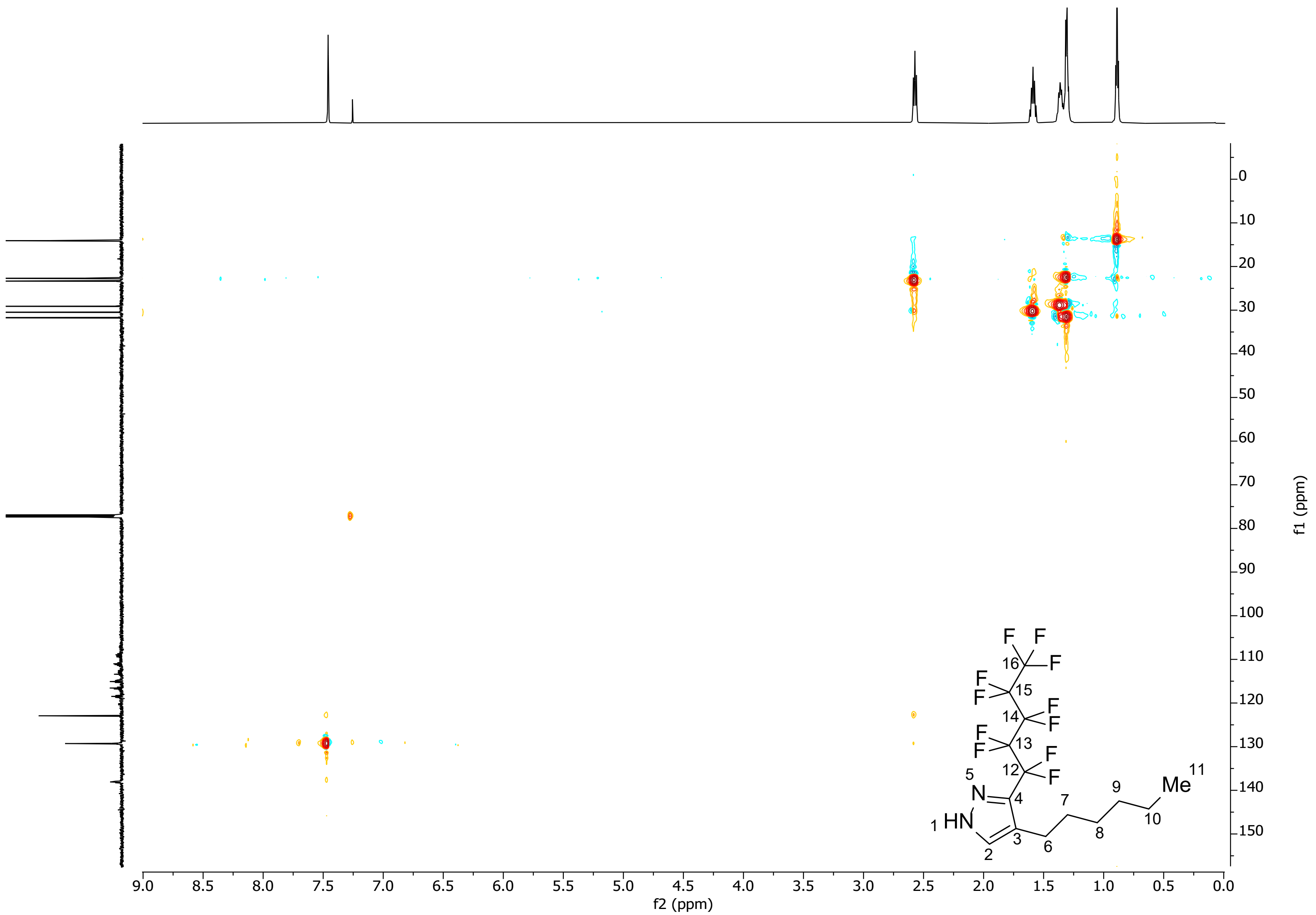

HSQC spectrum of compound **4b**

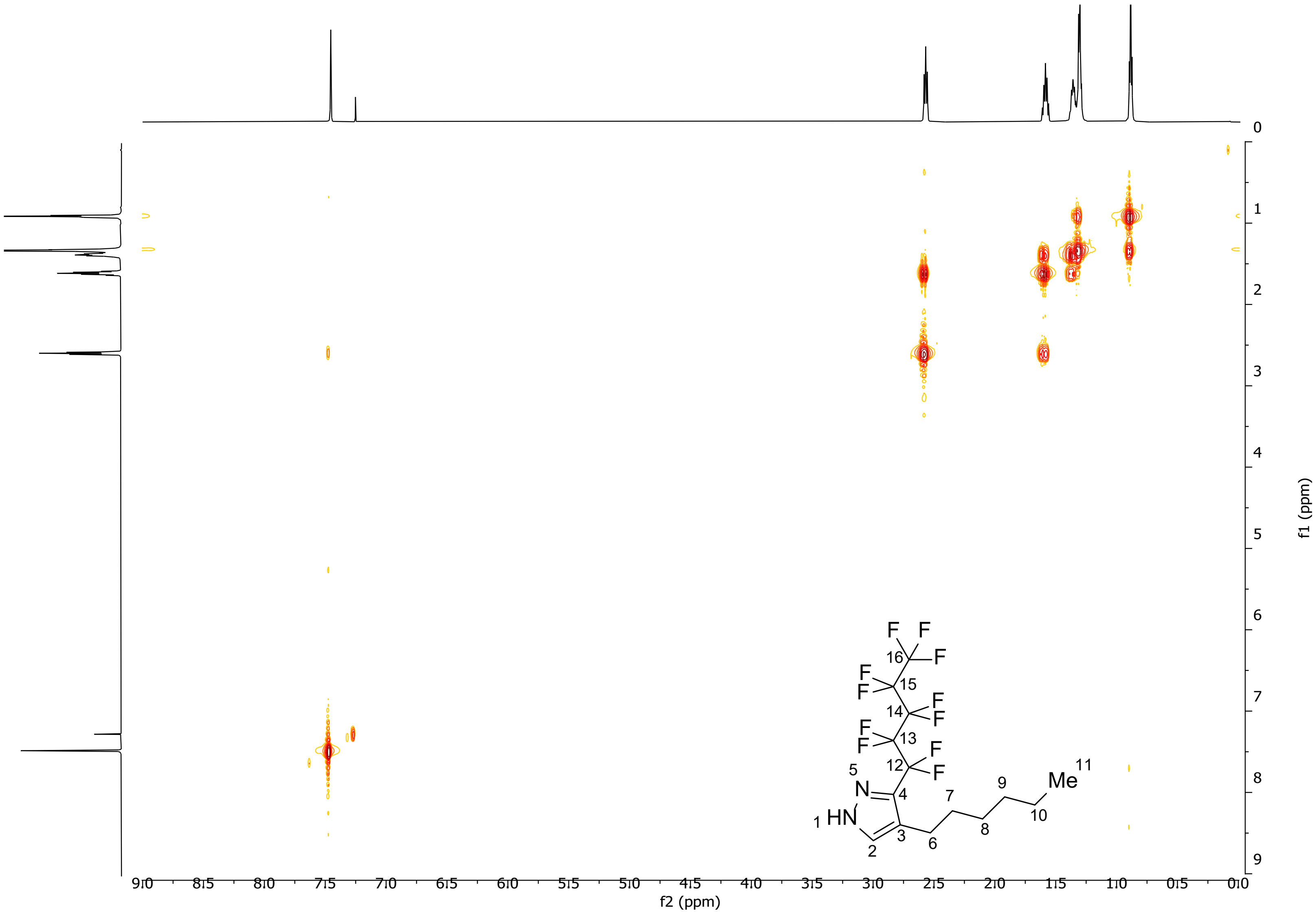

COSY spectrum of compound **4b**

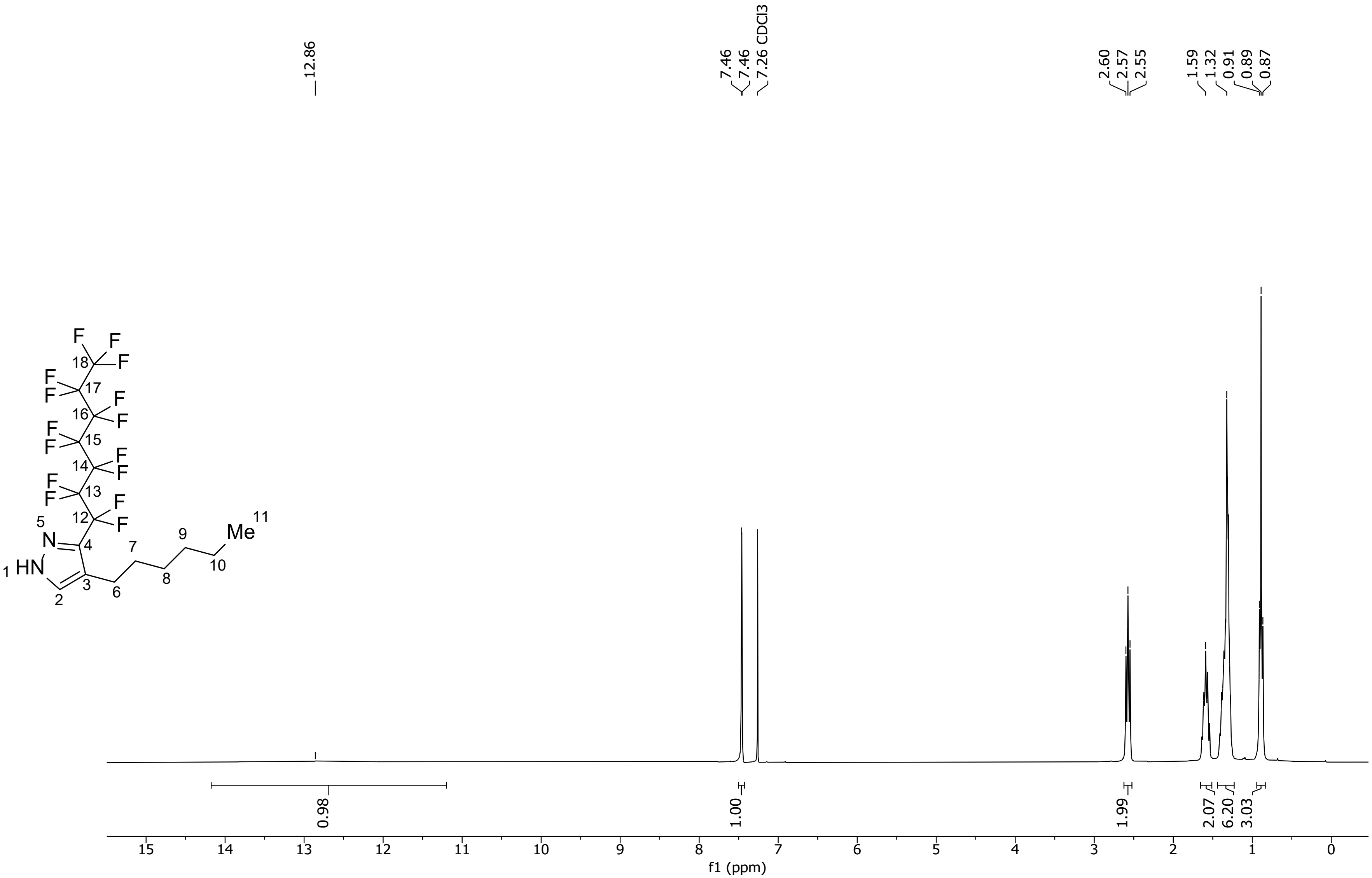

^1H NMR spectrum of compound **4c**

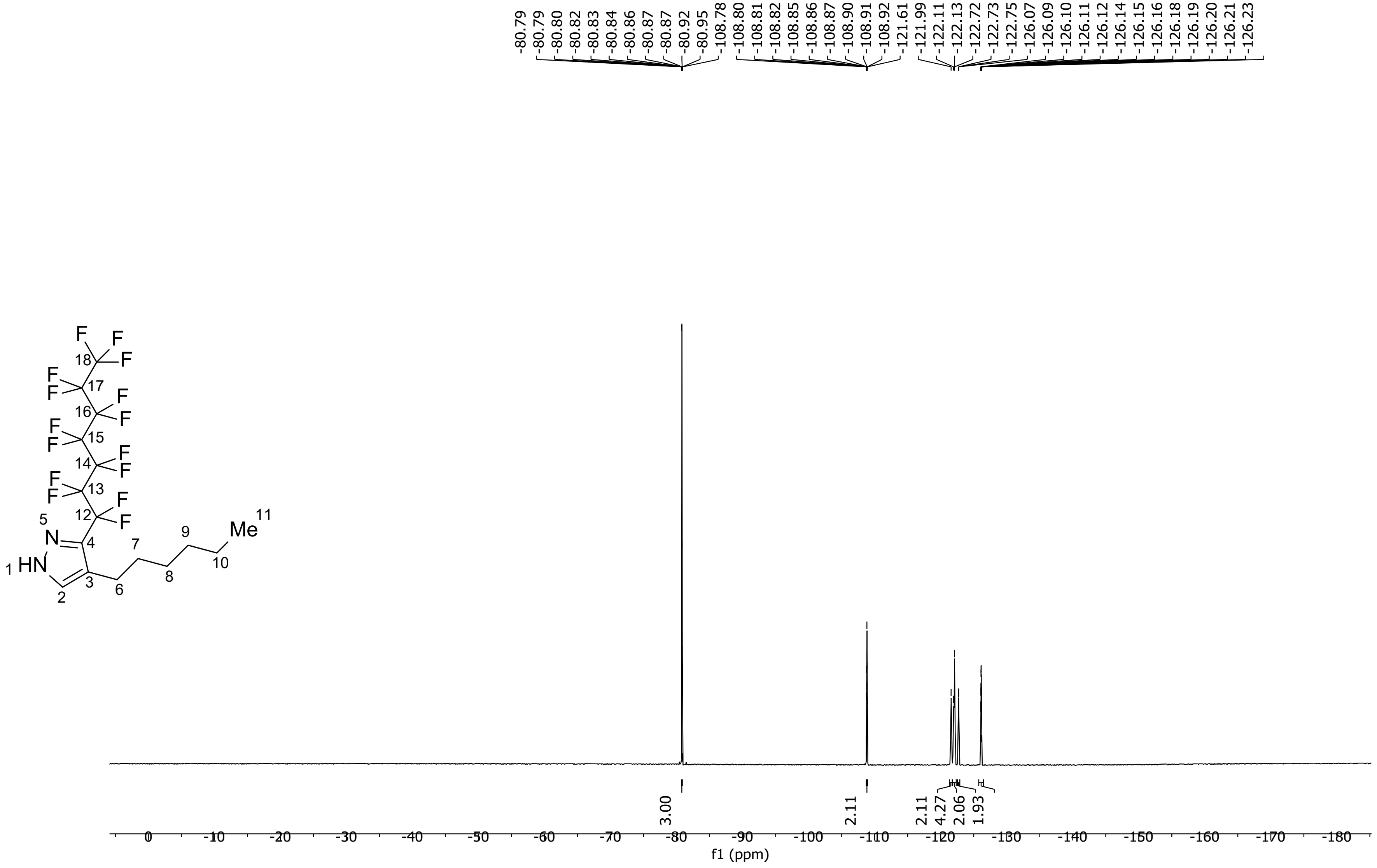

^{19}F NMR spectrum of compound **4c**

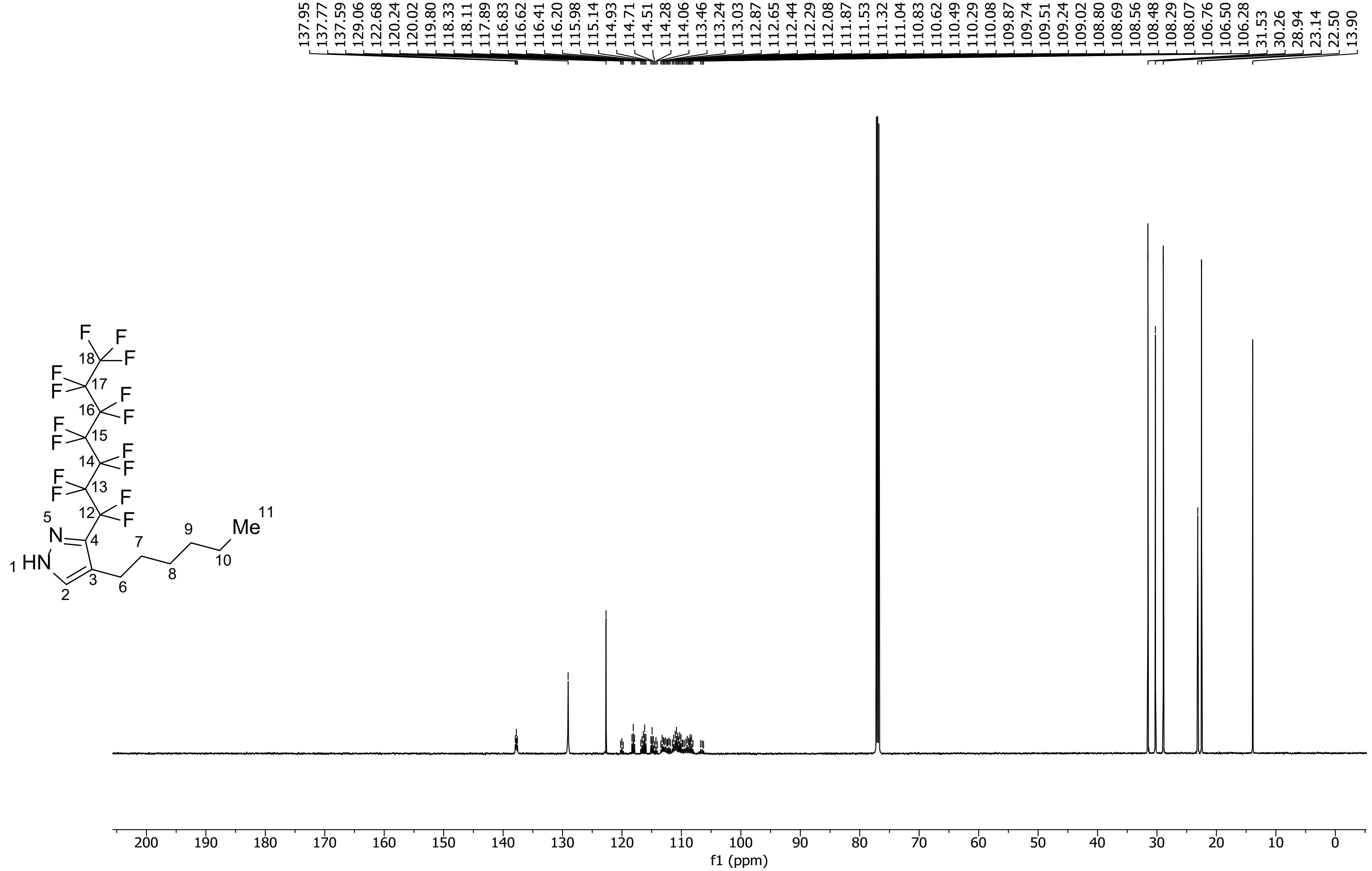

^{13}C NMR spectrum of compound **4c**

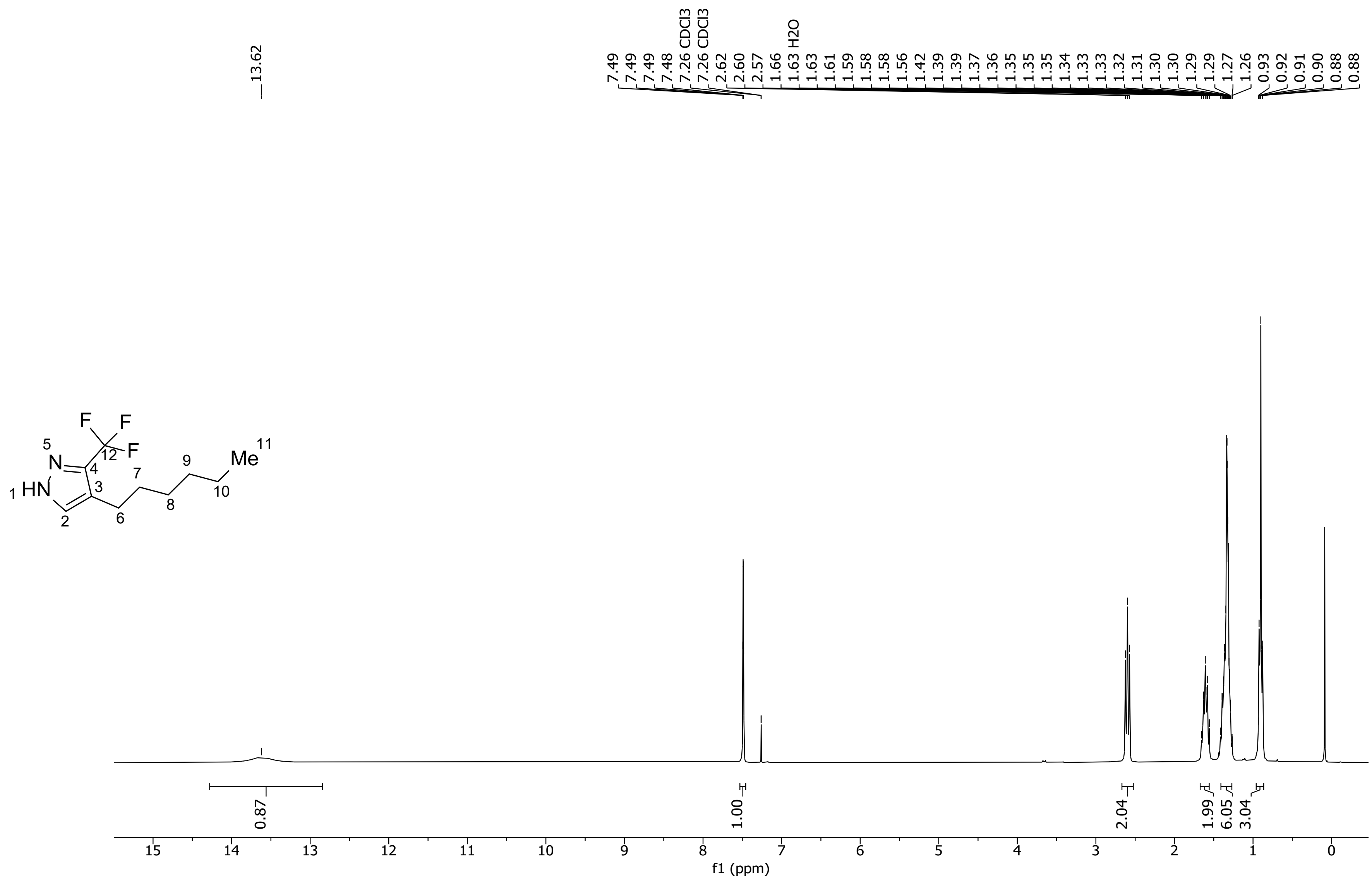

^{1}H NMR spectrum of compound **4d**

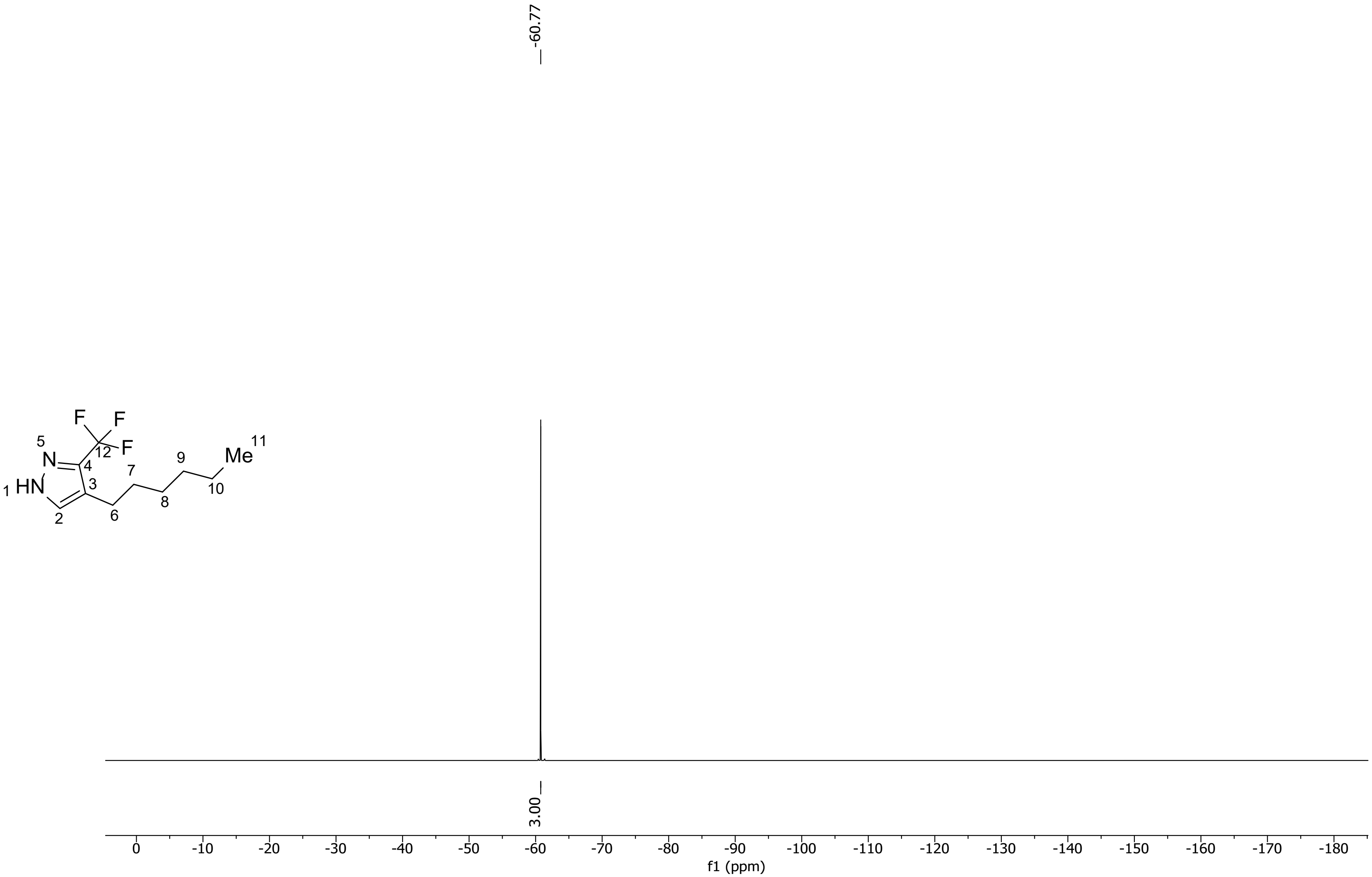

^{19}F NMR spectrum of compound **4d**

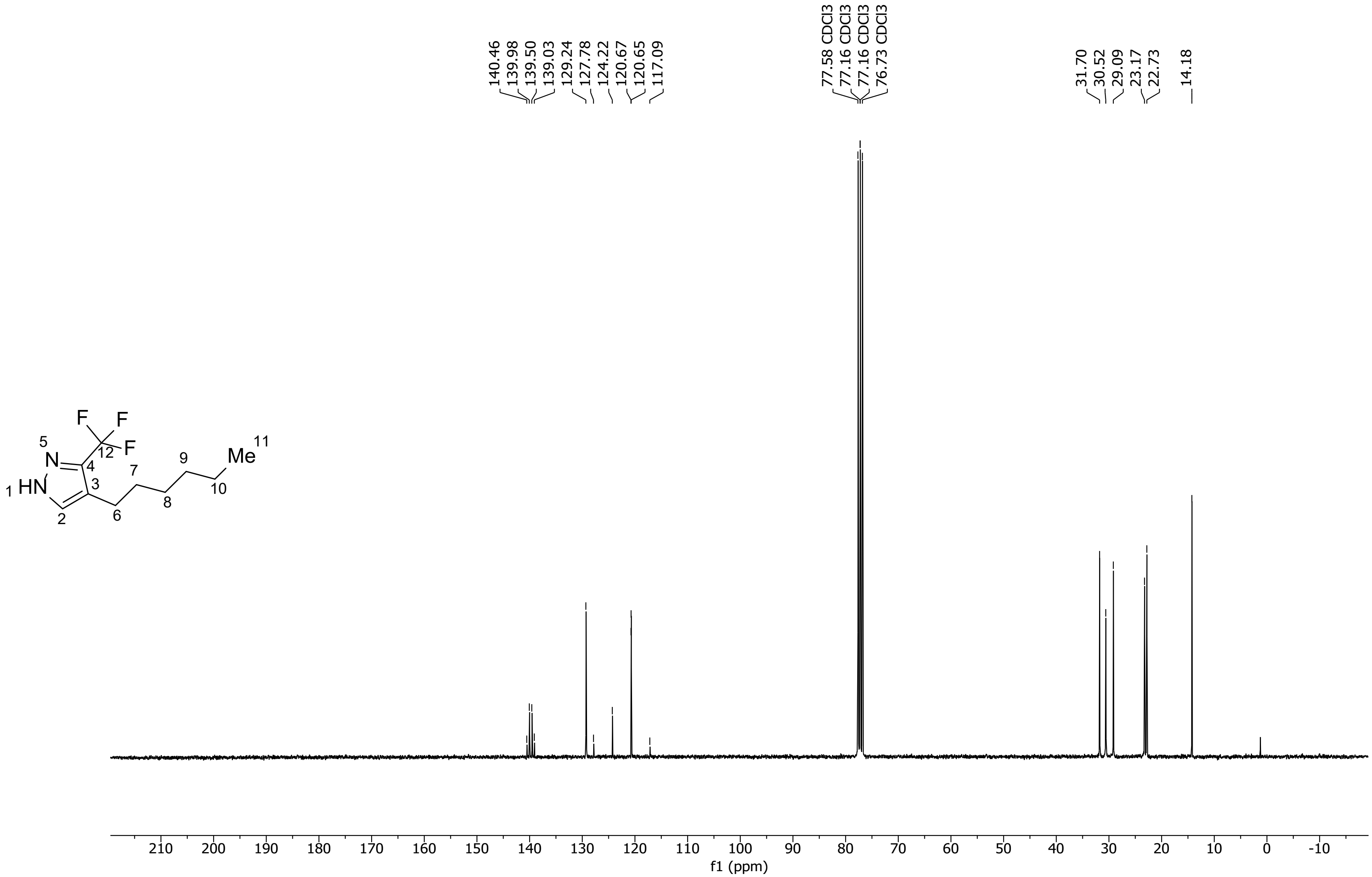

^{13}C NMR spectrum of compound **4d**

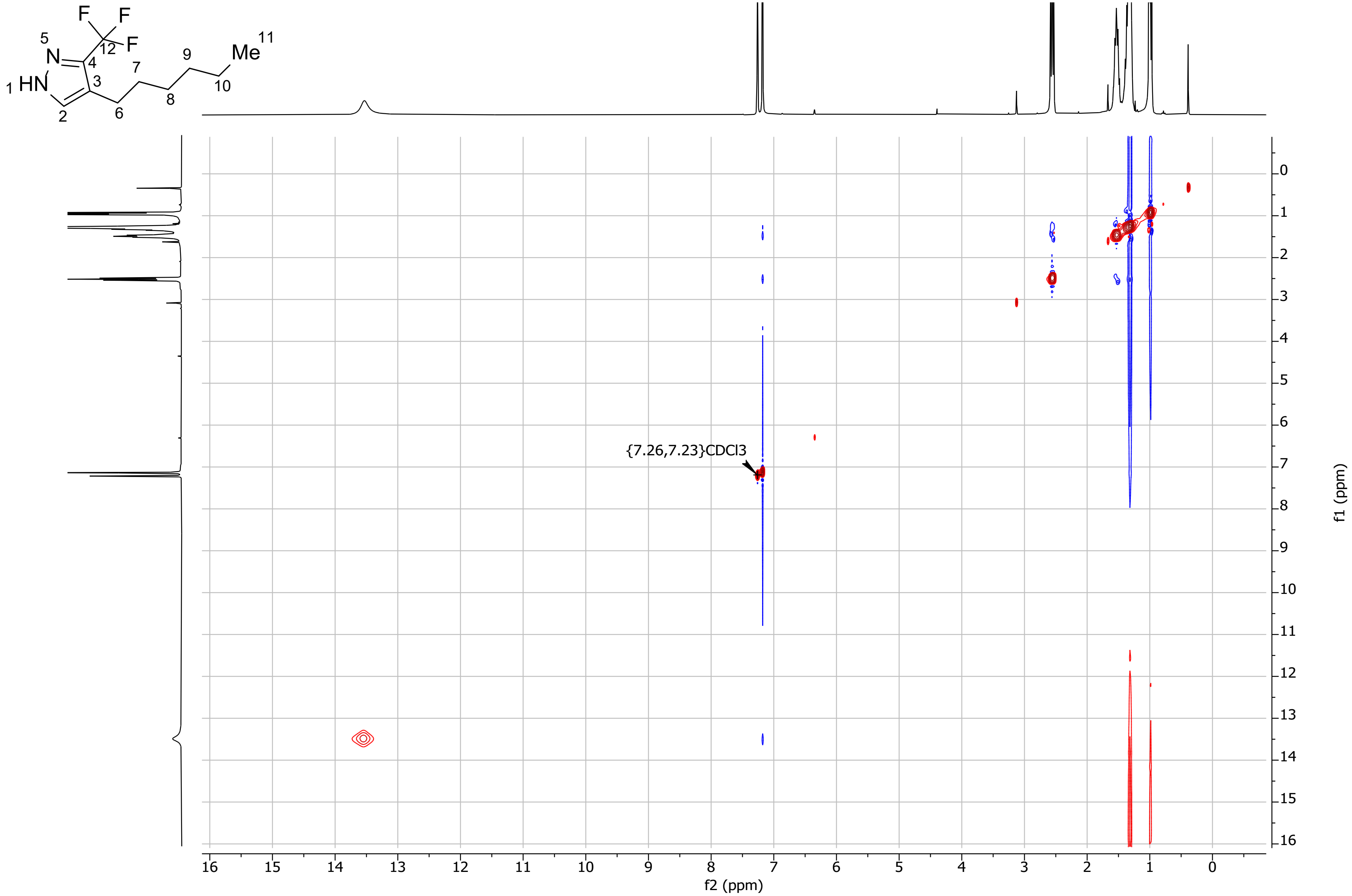

NOESY spectrum of compound **4d**

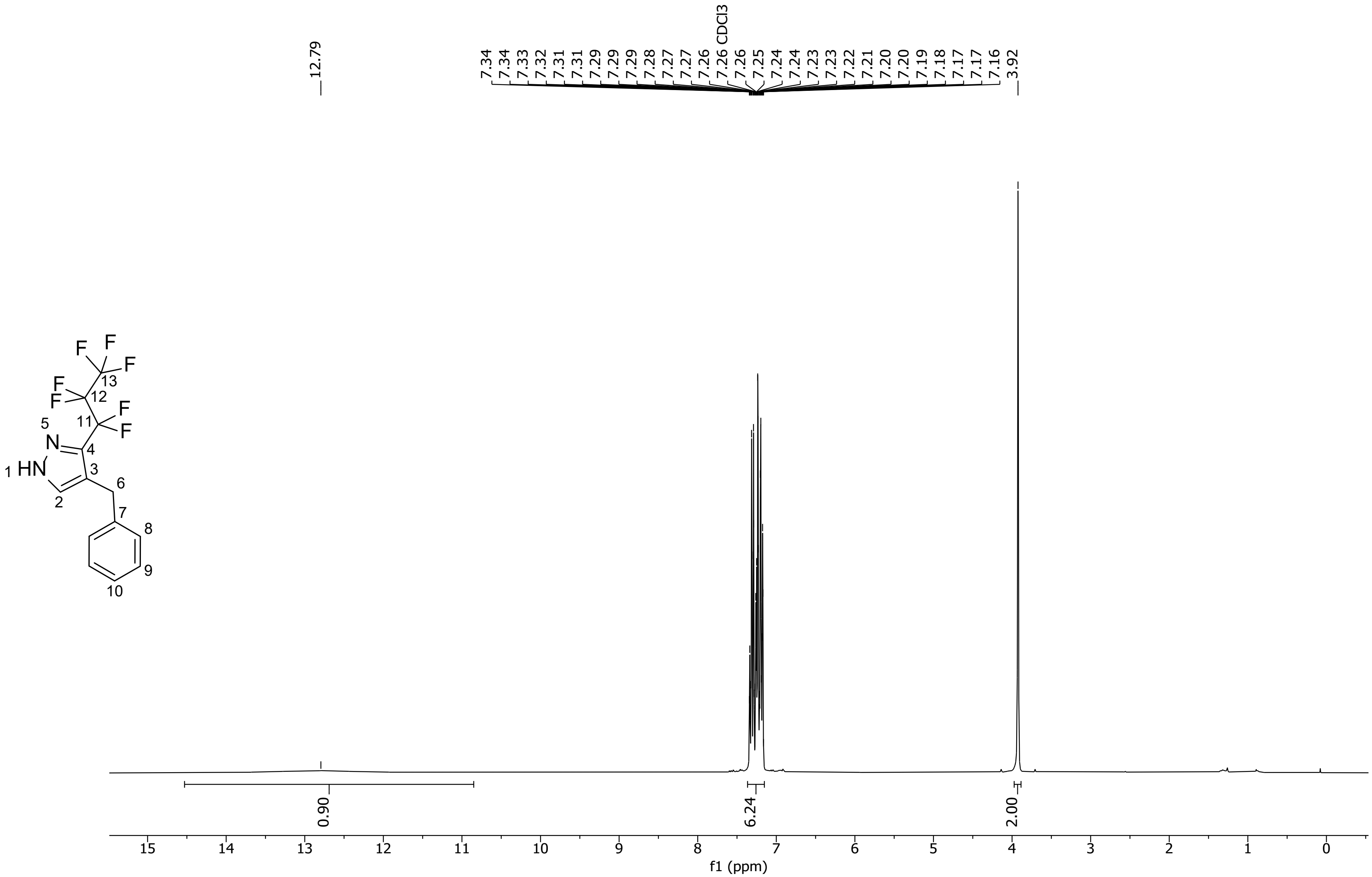

^{1}H NMR spectrum of compound **4e**

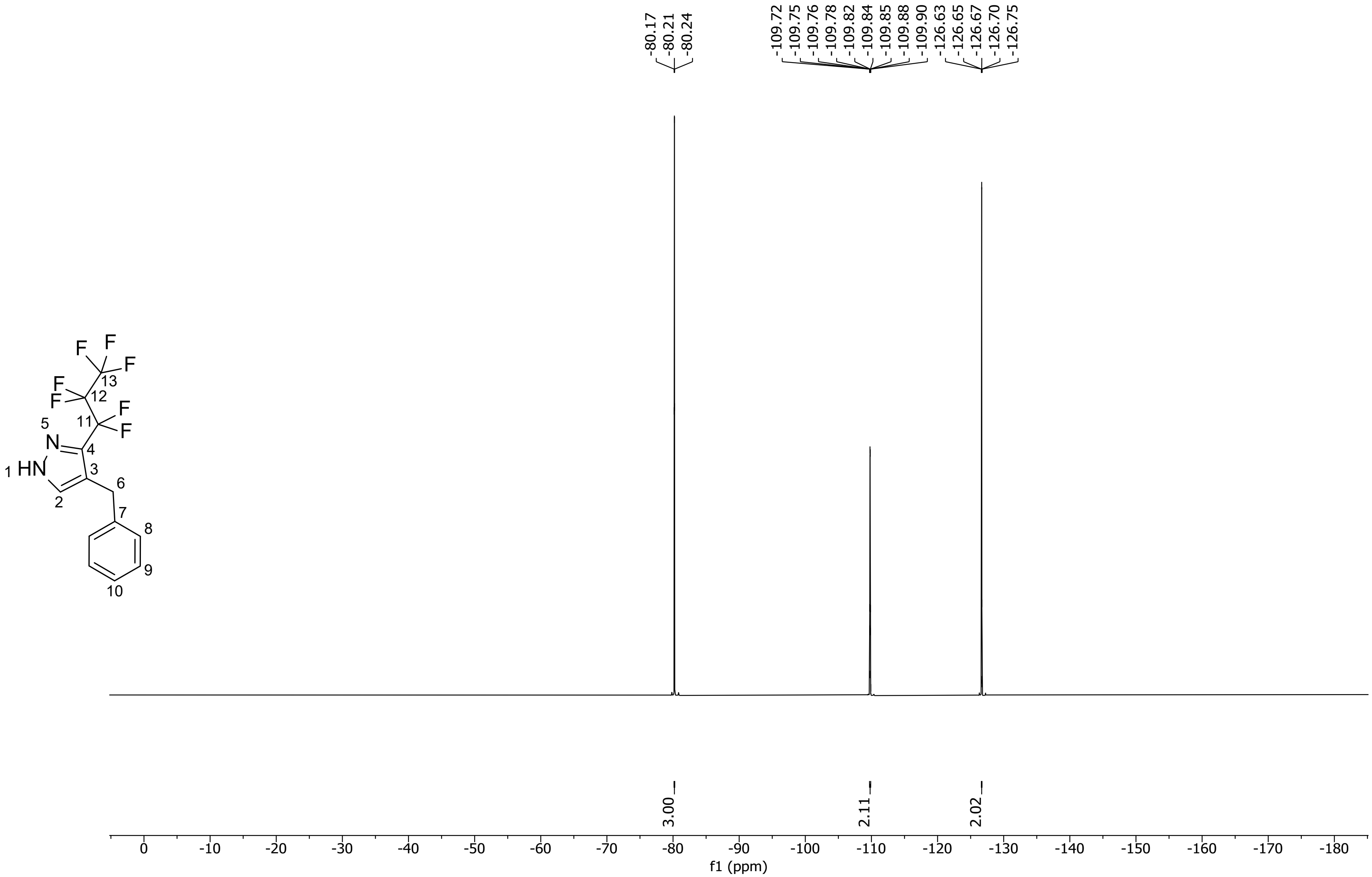

^{19}F NMR spectrum of compound **4e**

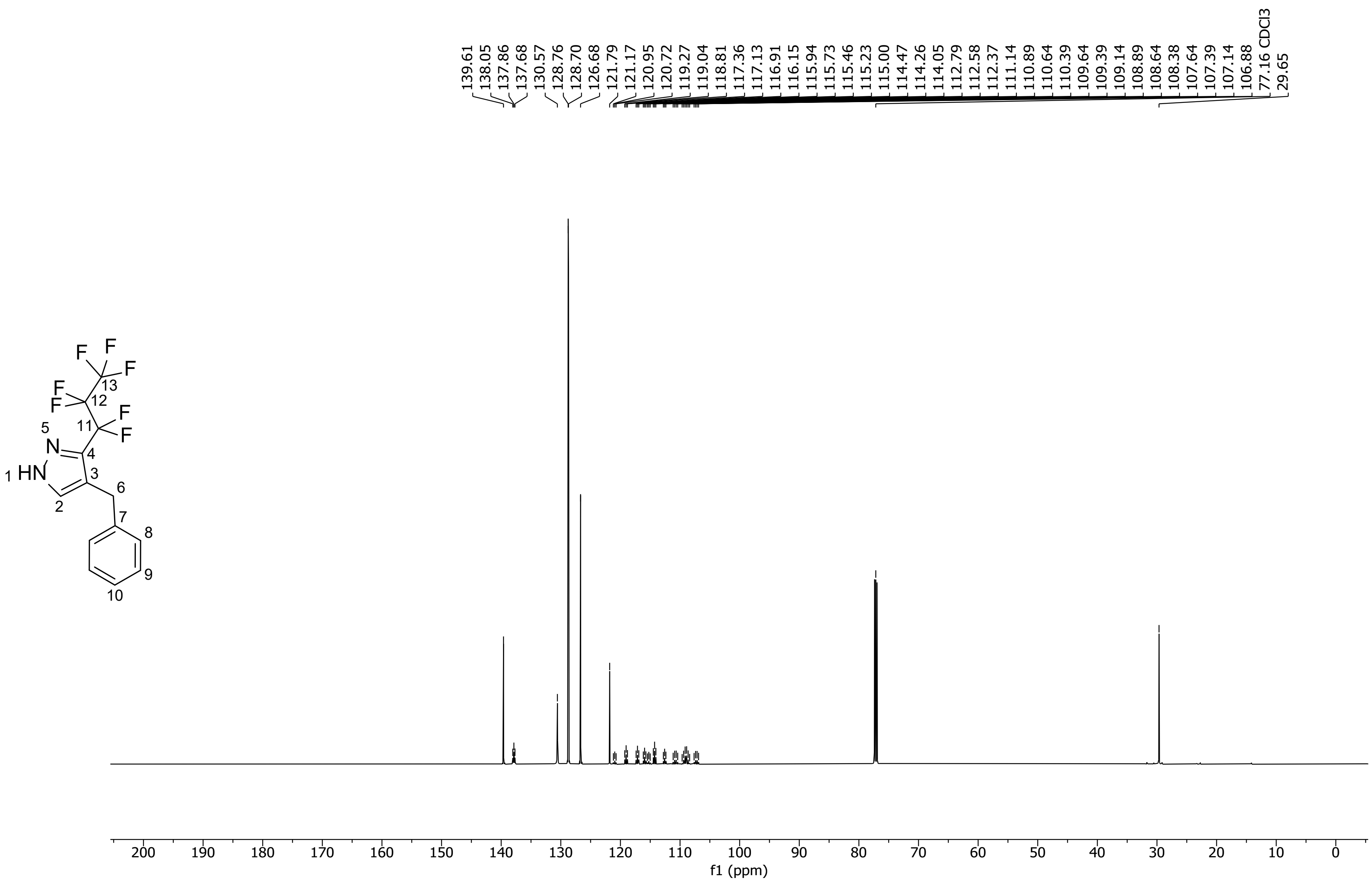

^{13}C NMR spectrum of compound **4e**

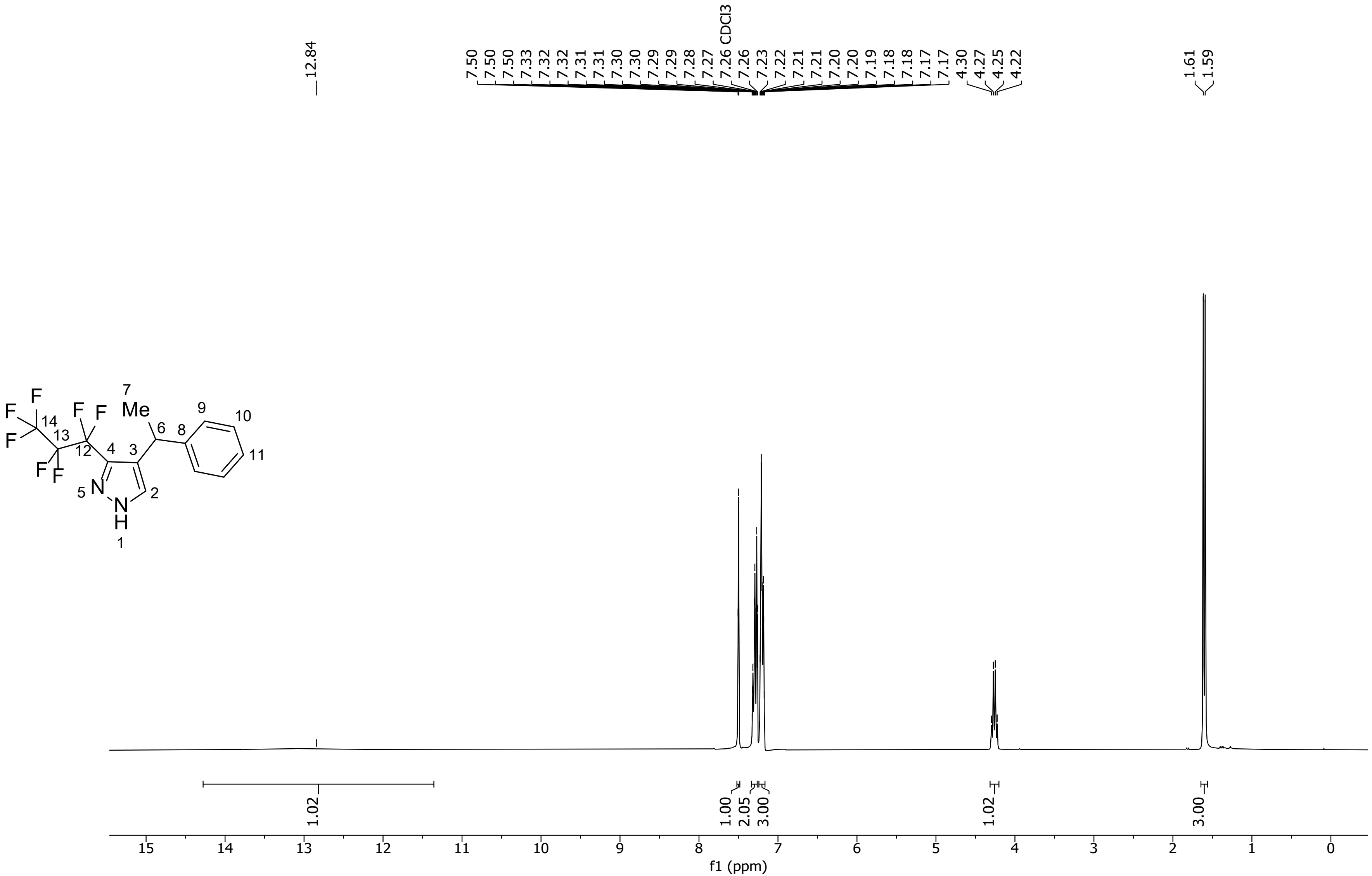

^{1}H NMR spectrum of compound **4f**

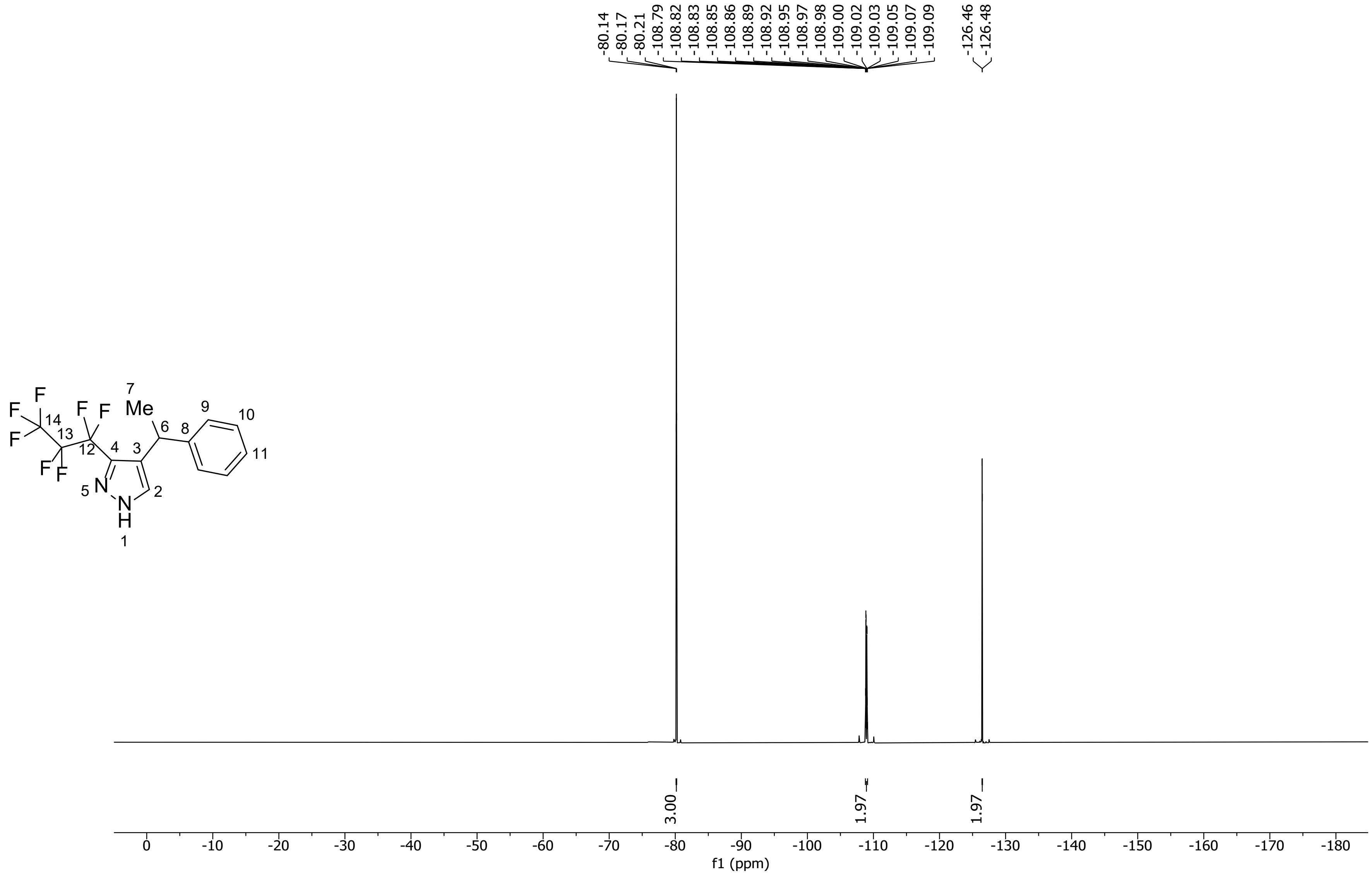

^{19}F NMR spectrum of compound **4f**

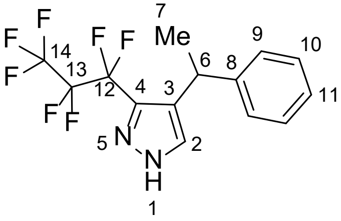

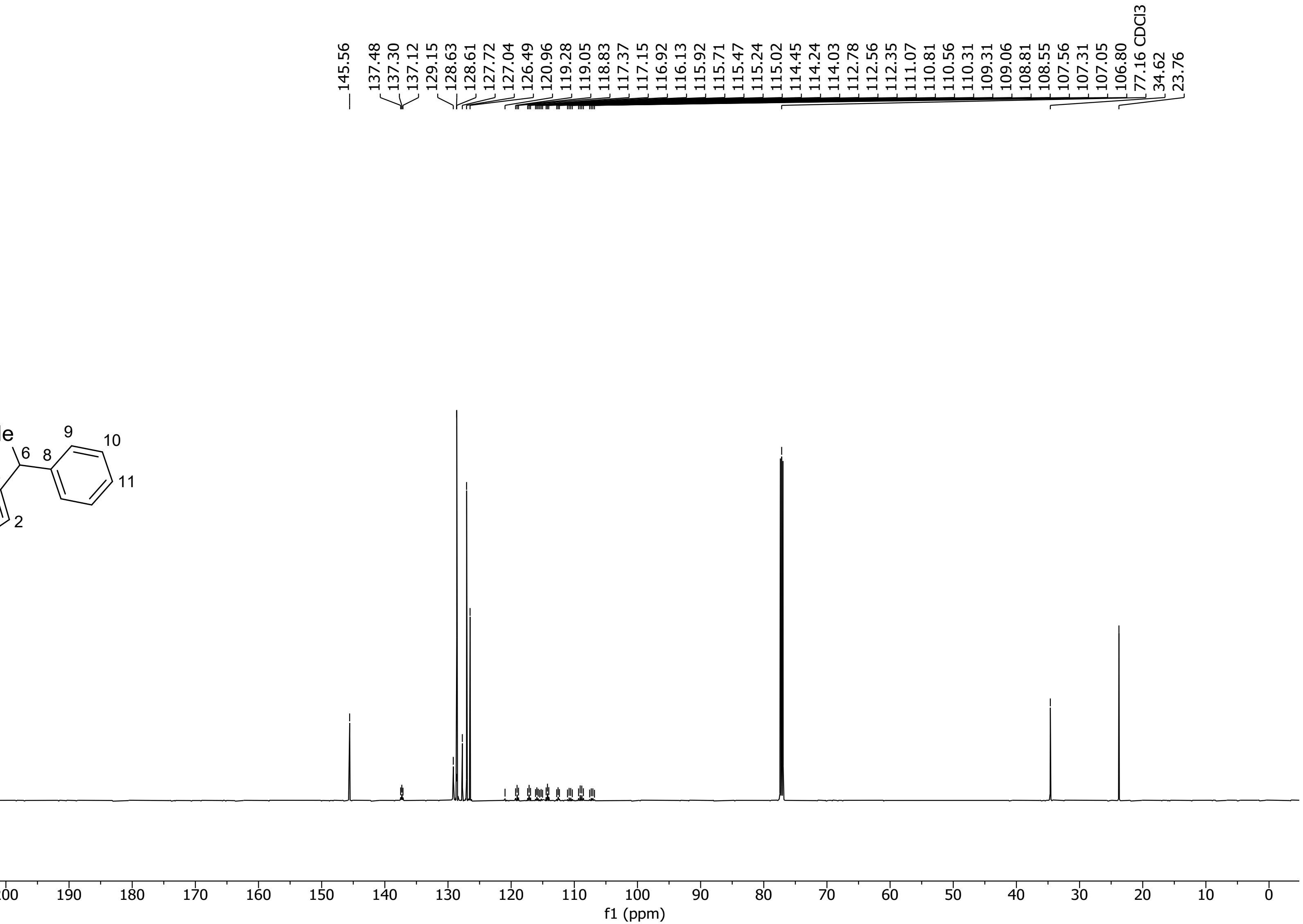

^{13}C NMR spectrum of compound **4f**

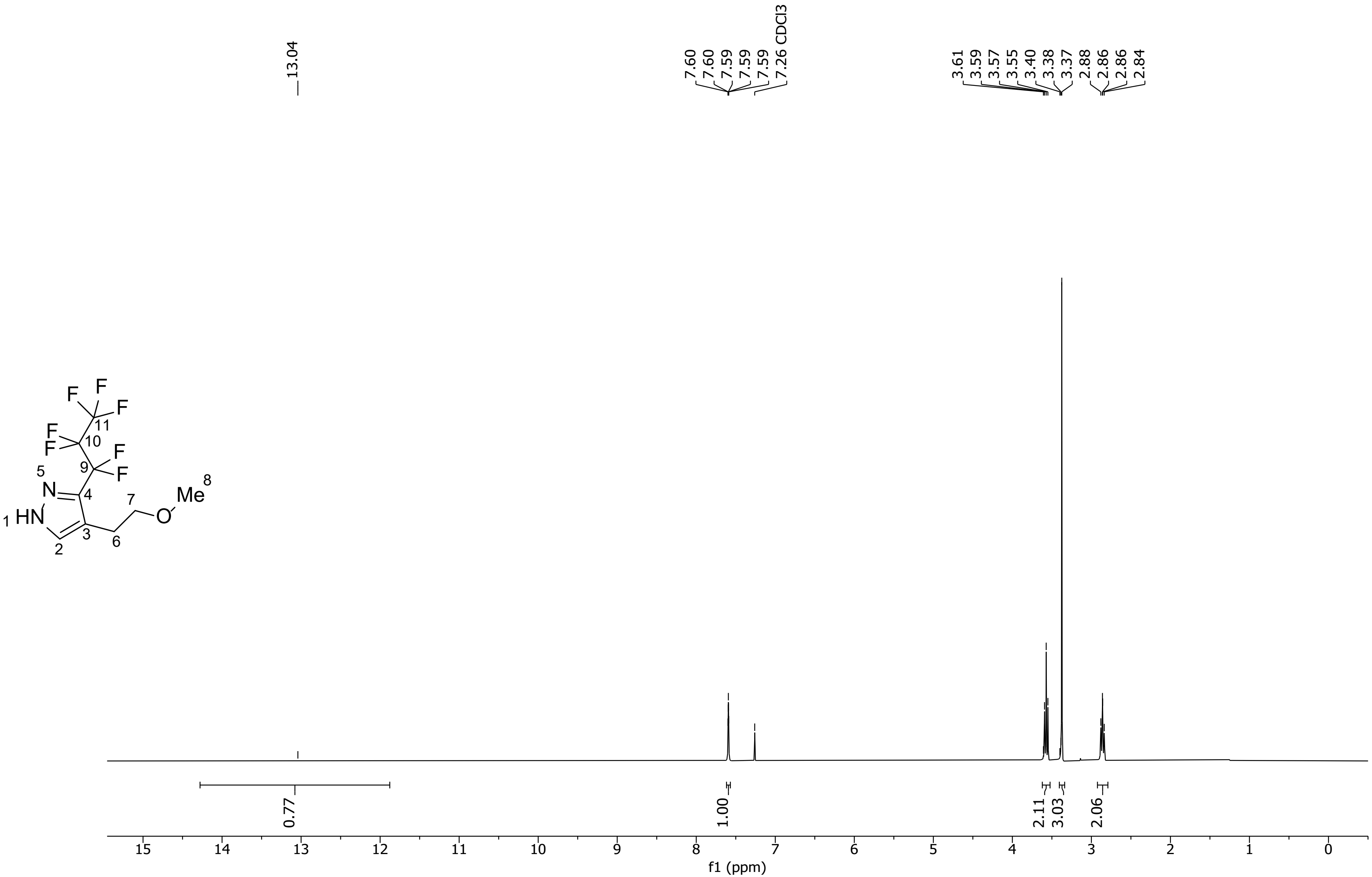

^{1}H NMR spectrum of compound **4g**

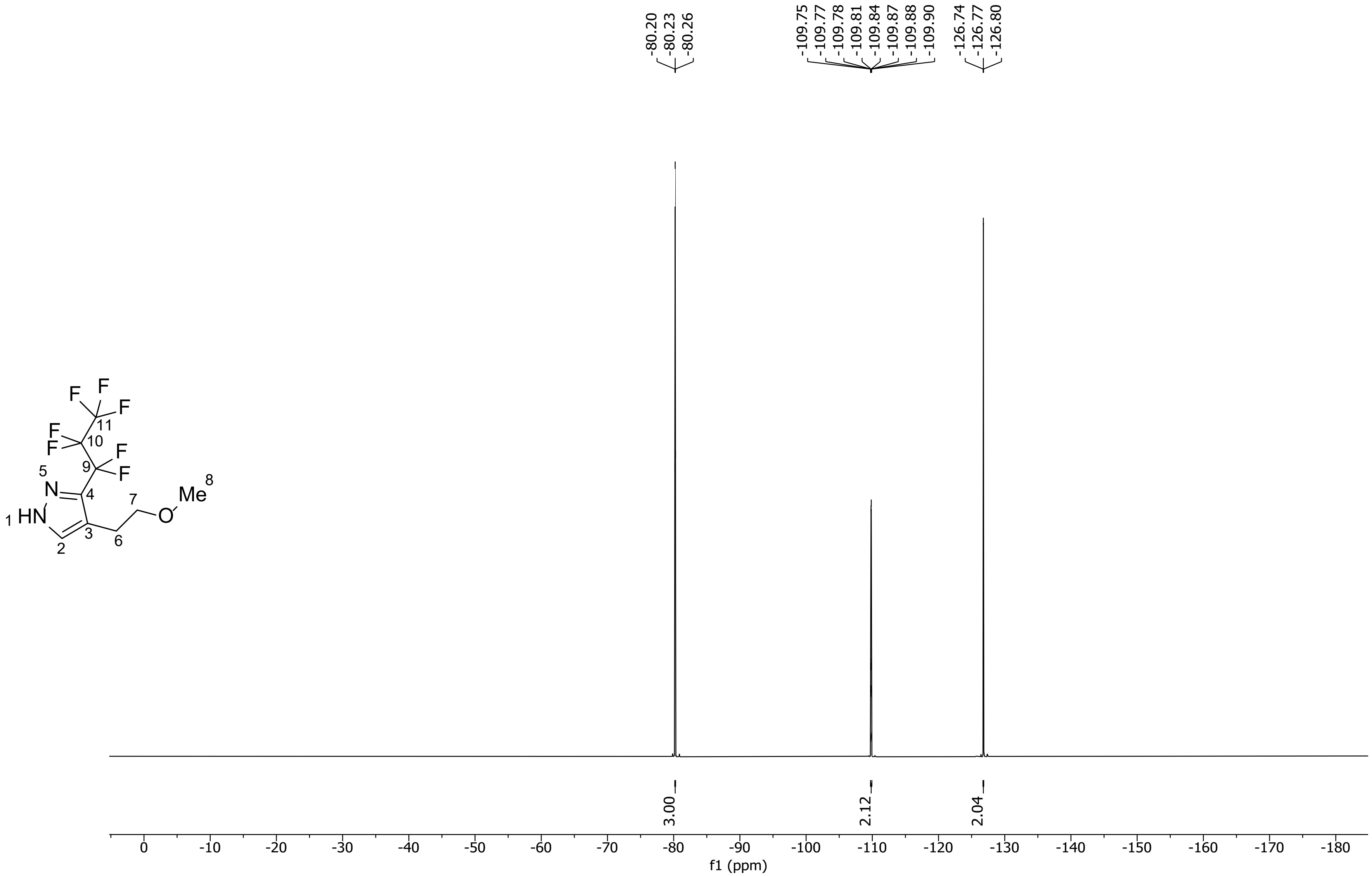

^{19}F NMR spectrum of compound **4g**

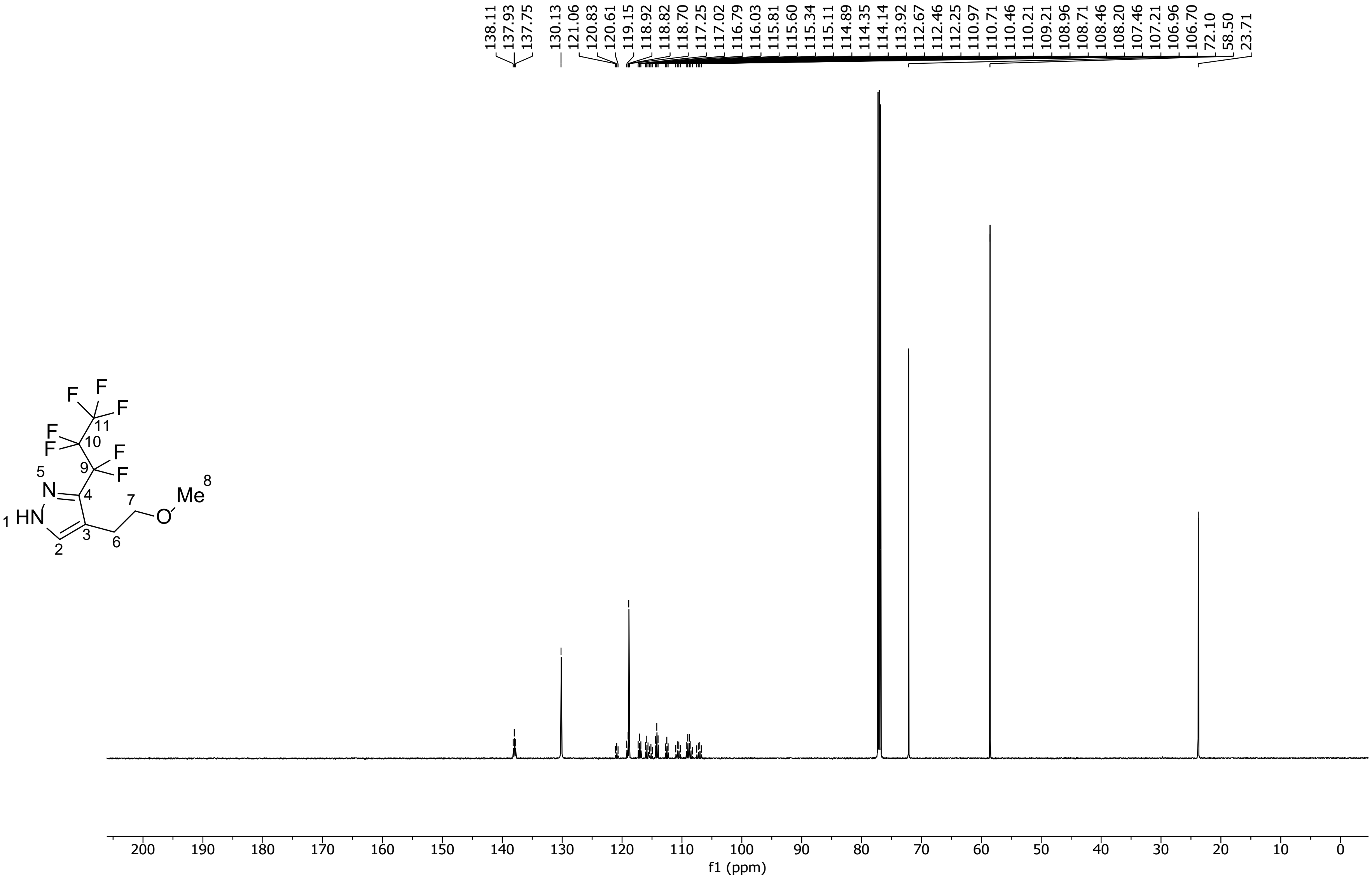

^{13}C NMR spectrum of compound **4g**

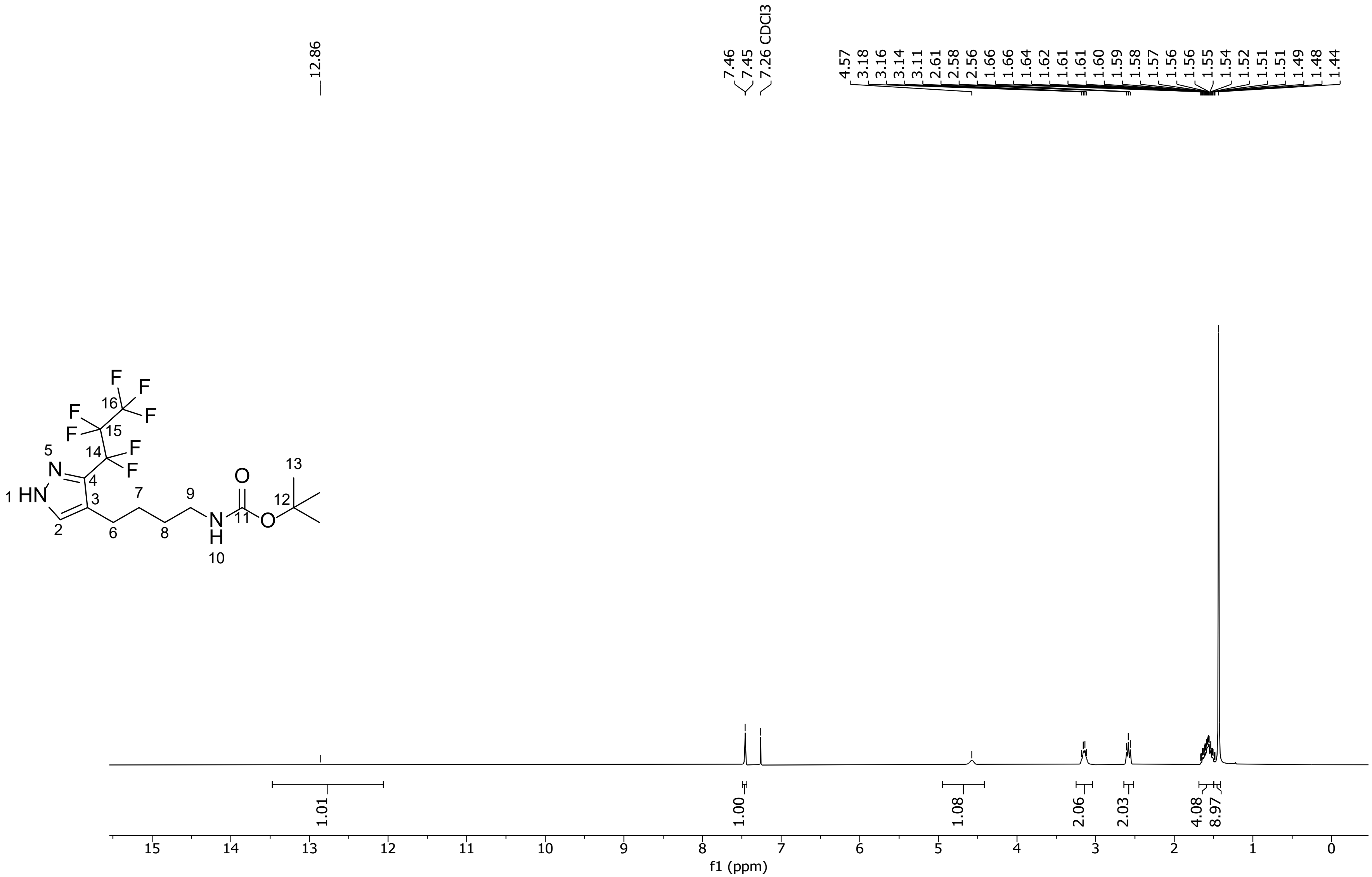

^{1}H NMR spectrum of compound **4h**

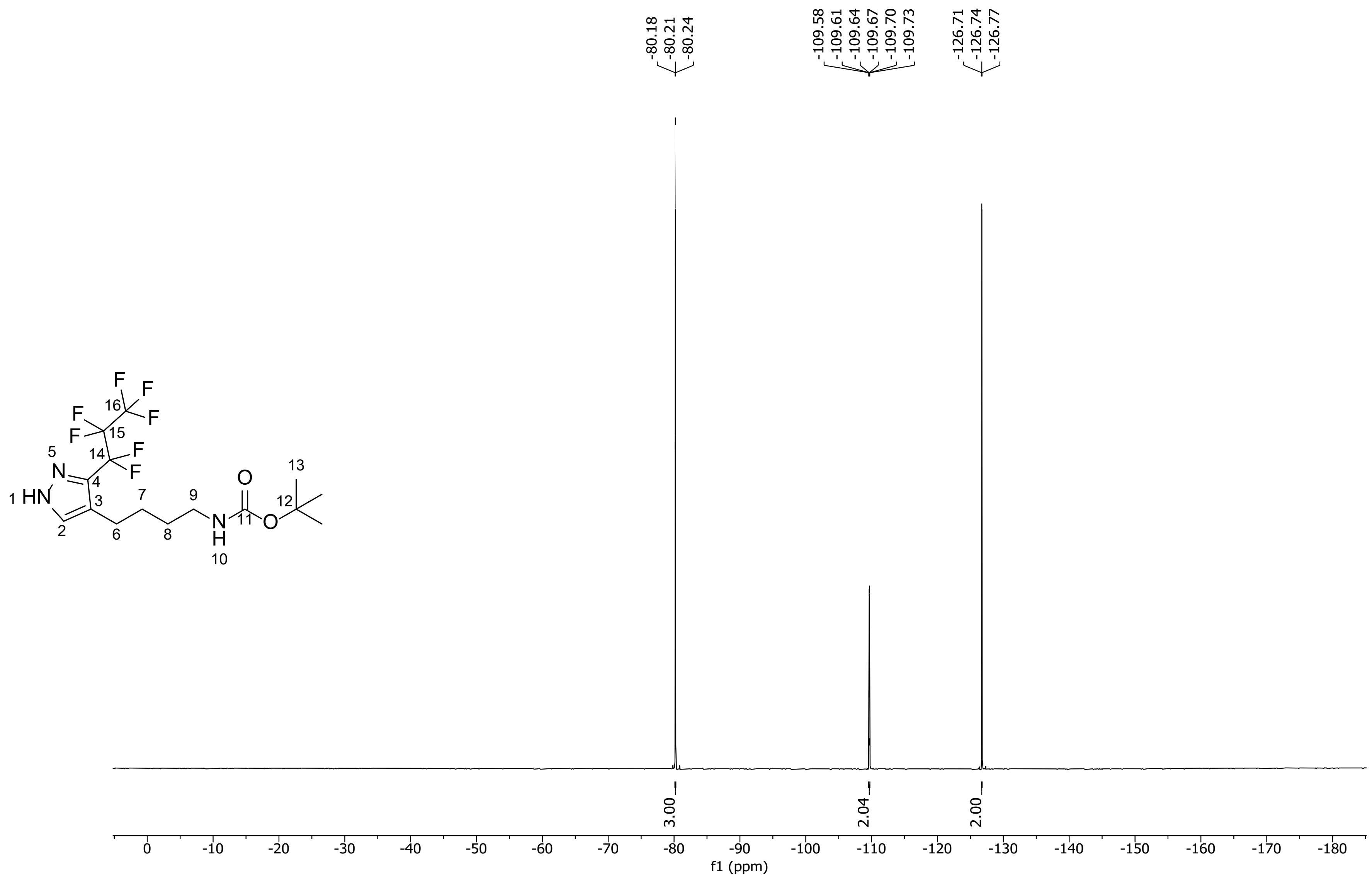

^{19}F NMR spectrum of compound **4h**

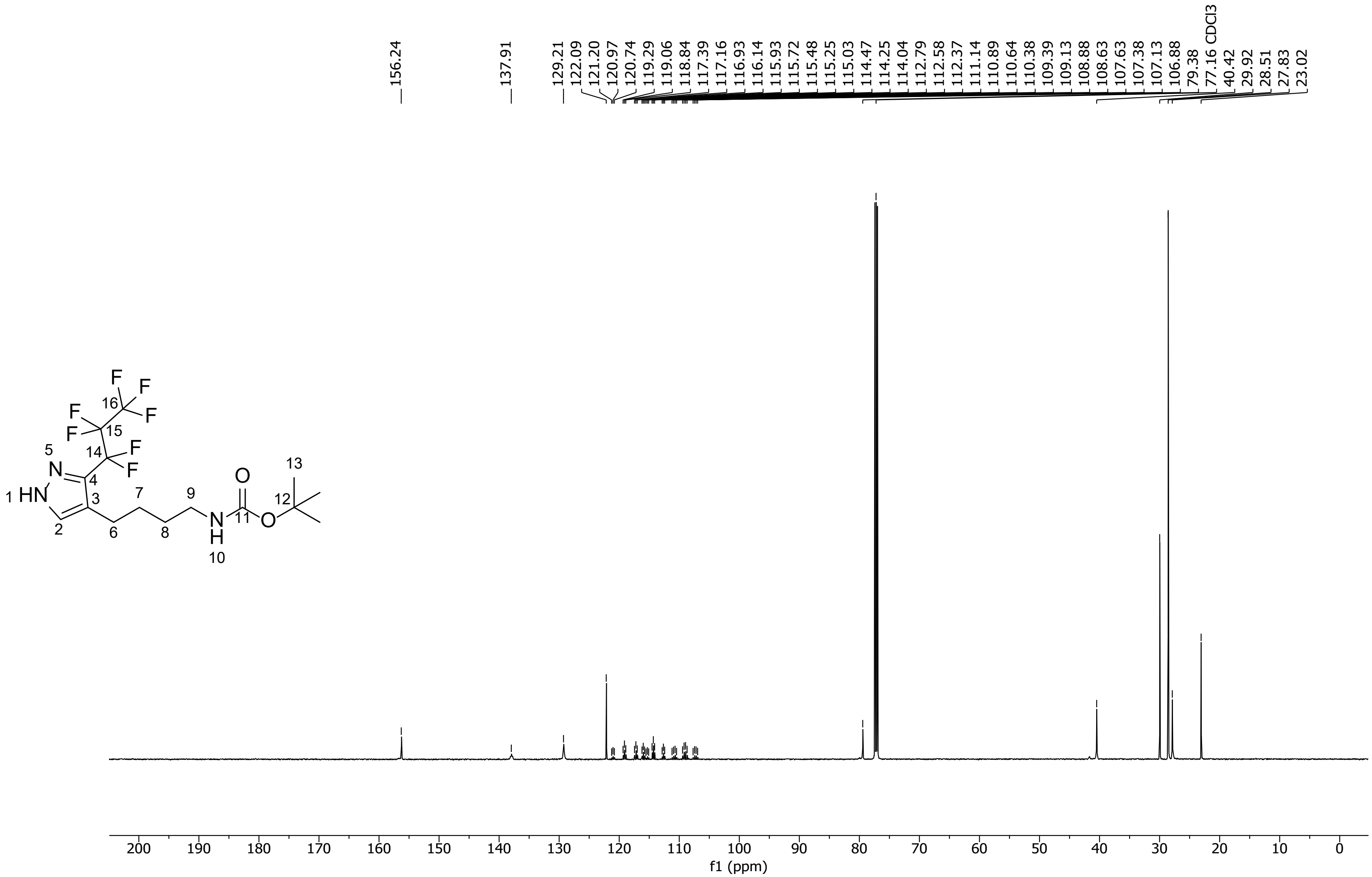

^{13}C NMR spectrum of compound **4h**

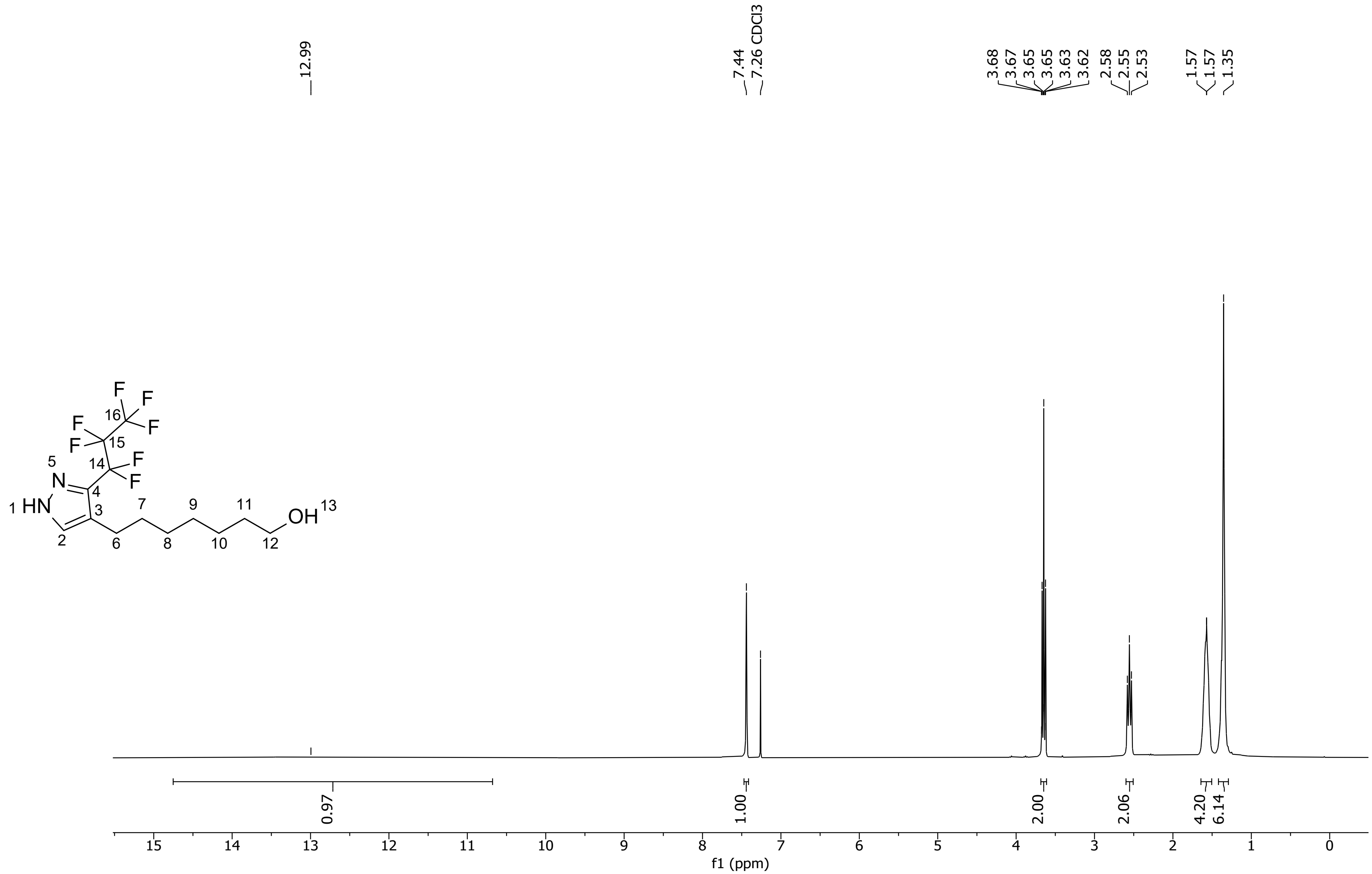

^{1}H NMR spectrum of compound **4i**

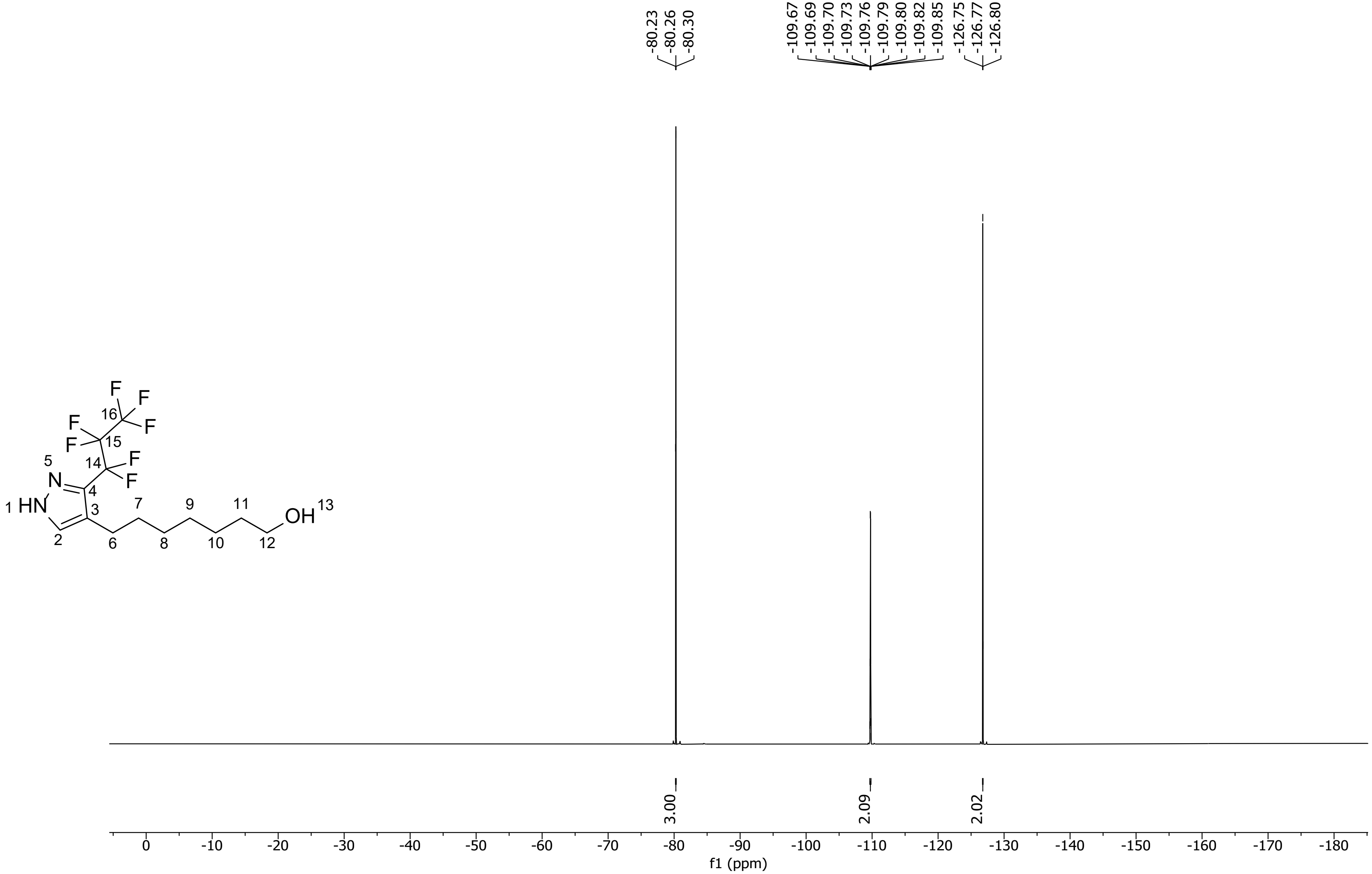

^{19}F NMR spectrum of compound **4i**

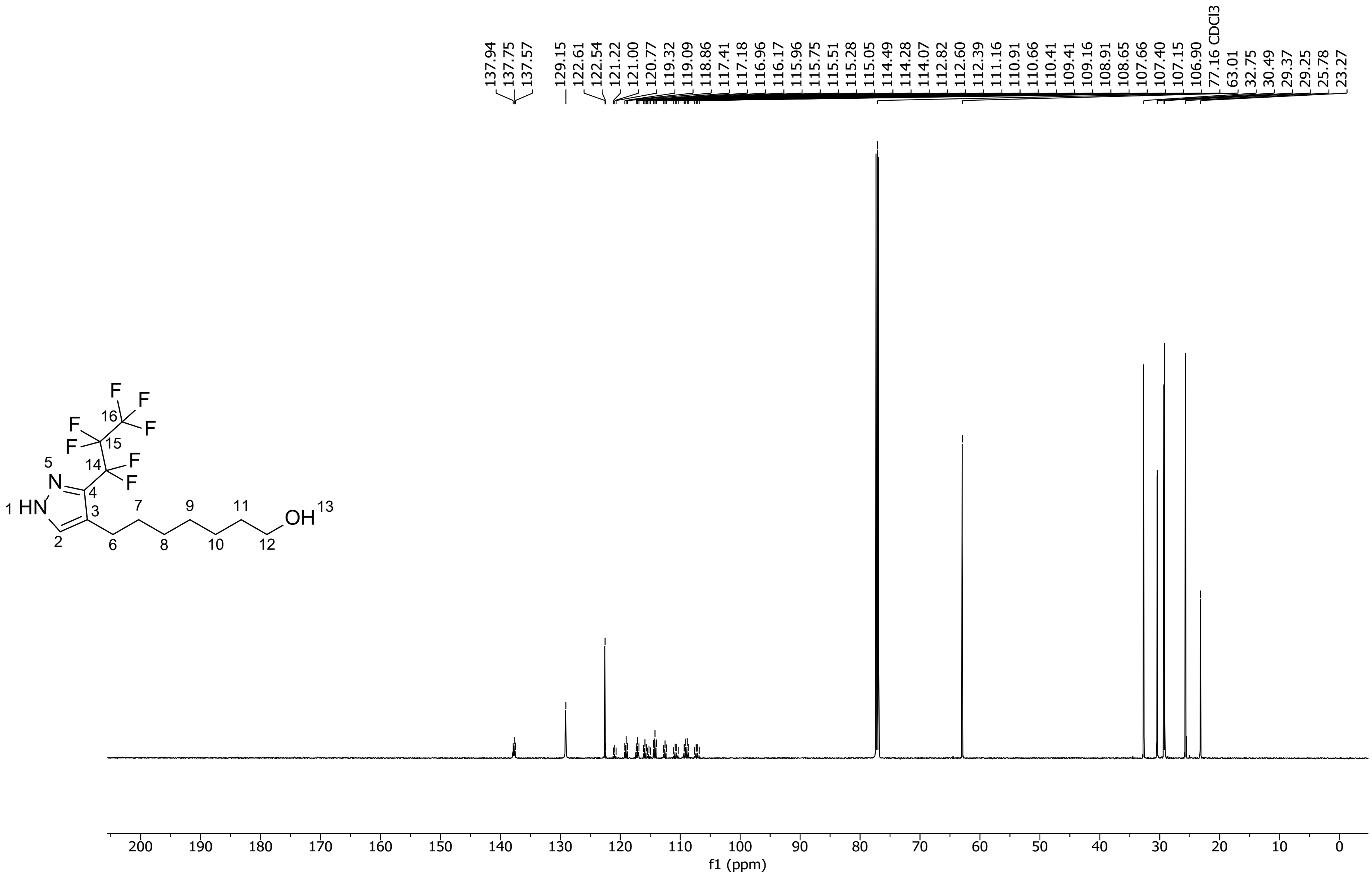

^{13}C NMR spectrum of compound **4i**

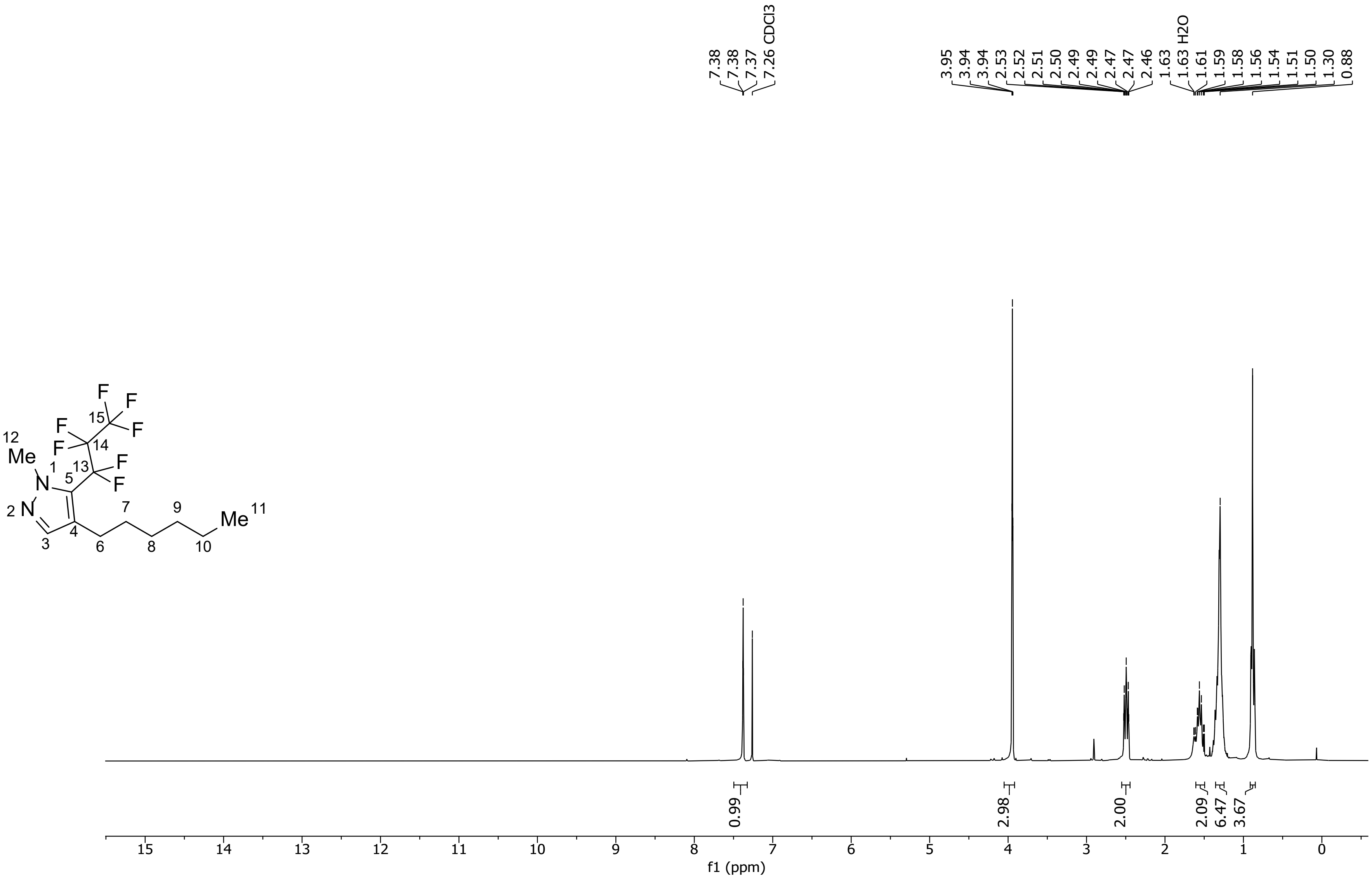

^{1}H NMR spectrum of compound **4j-2**

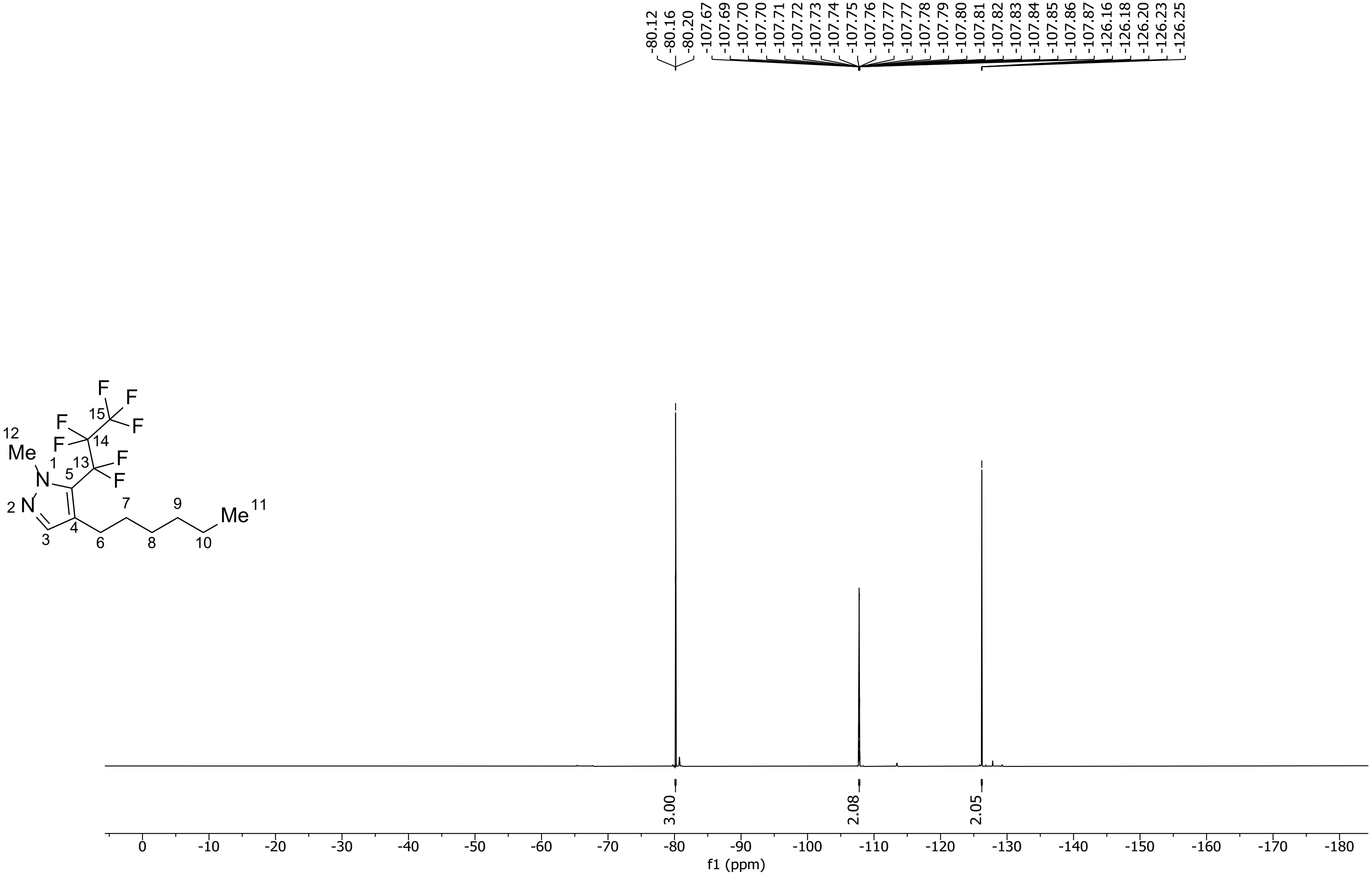

^{19}F NMR spectrum of compound **4j-2**

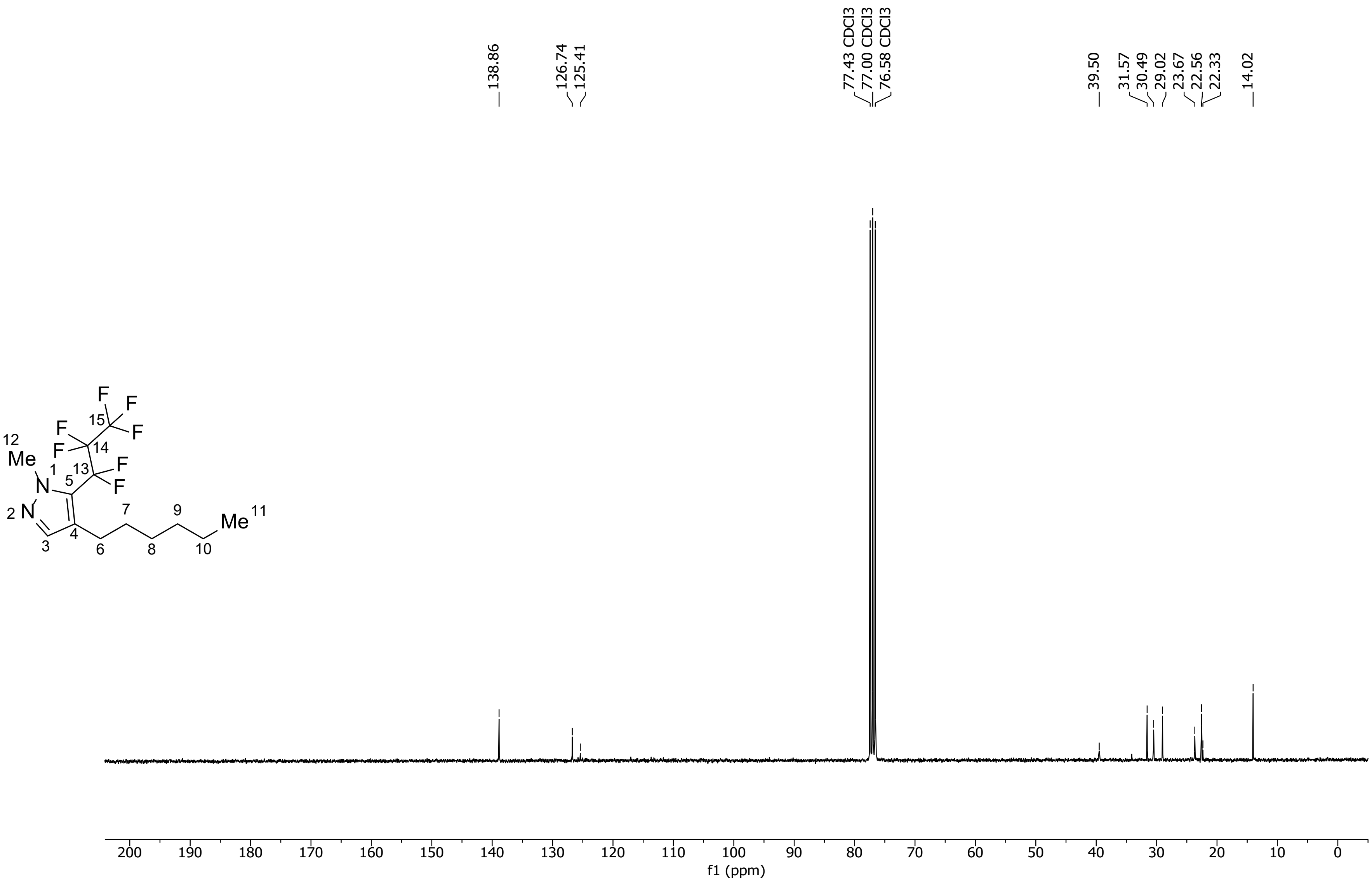

^{13}C NMR spectrum of compound **4j-2**

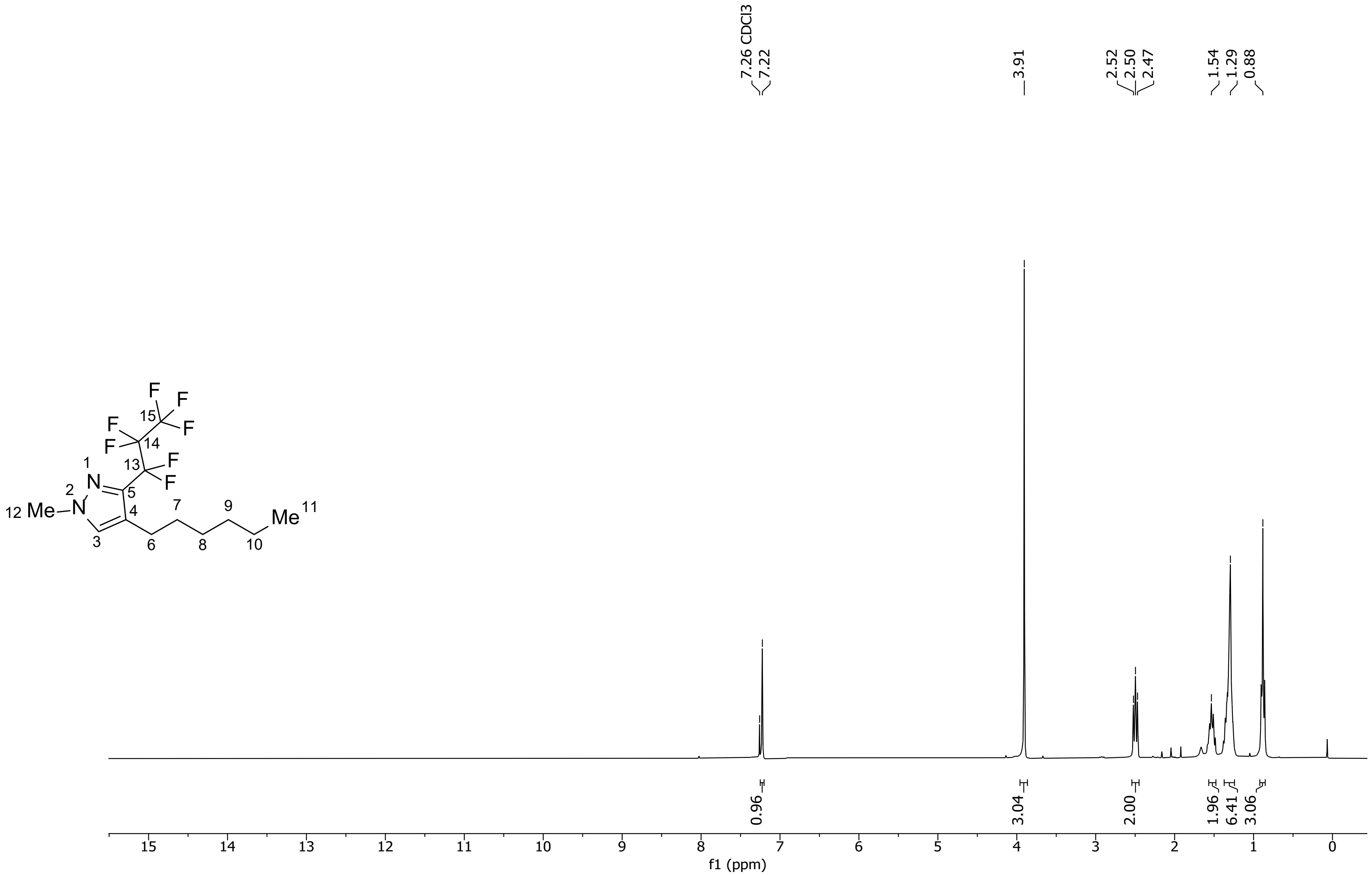

^{1}H NMR spectrum of compound **4j-1**

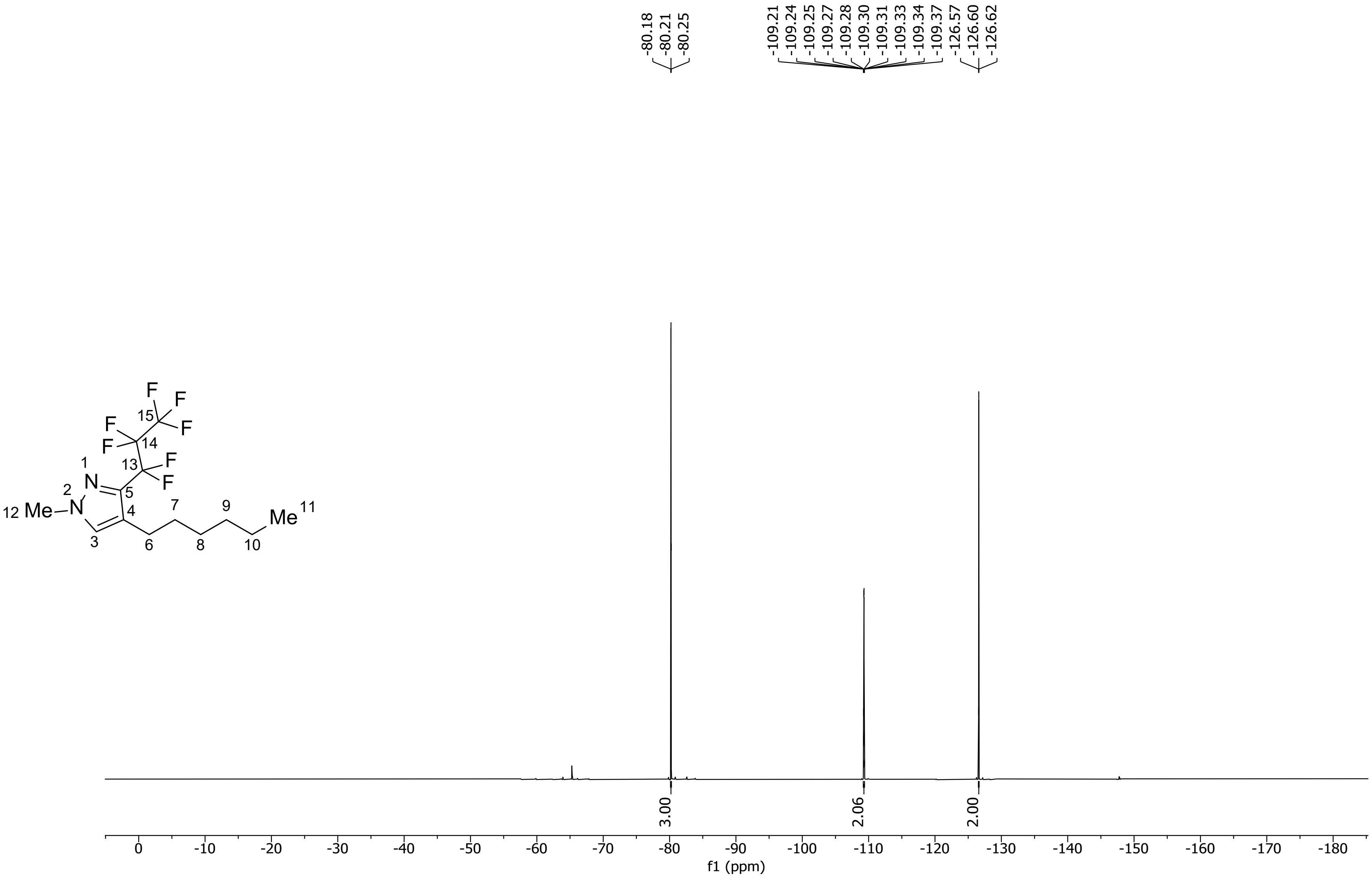

^{19}F NMR spectrum of compound **4j-1**

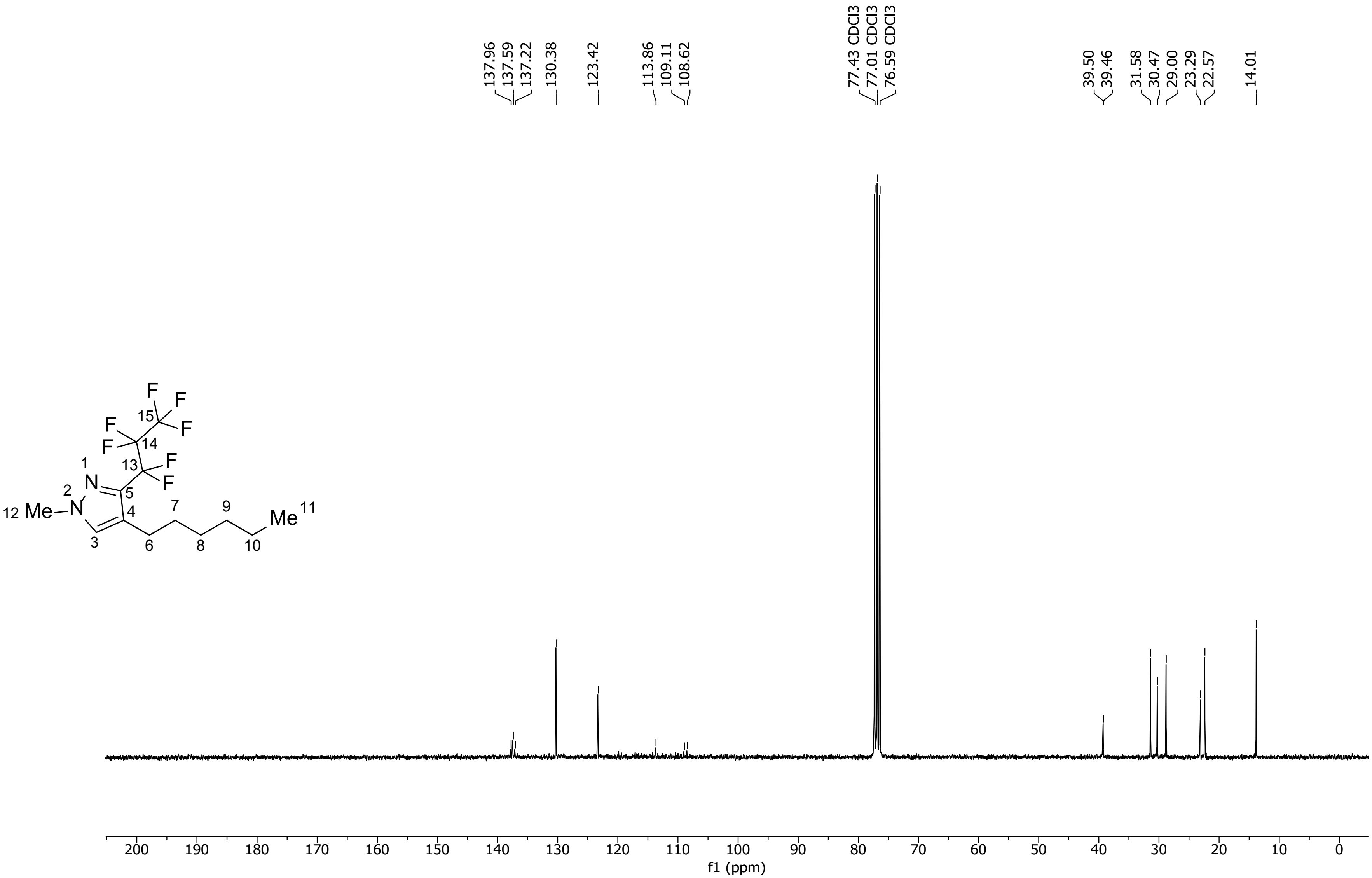

^{13}C NMR spectrum of compound **4j-1**

Mass Spectrum SmartFormula Report

Analysis Info

Acquisition Date 8/3/2022 3:36:41 PM

Analysis Name	D:\Data\Spektren 2022\CZE22HR000089.d		
Method	tune_low_new.m	Operator	PT
Sample Name	Bunnemann LB_V27 (CH3OH)	Instrument	maXis 288882.20213
Comment			

Acquisition Parameter

Source Type	ESI	Ion Polarity	Positive	Set Nebulizer	0.3 Bar
Focus	Not active	Set Capillary	4000 V	Set Dry Heater	180 °C
Scan Begin	50 m/z	Set End Plate Offset	-500 V	Set Dry Gas	4.0 l/min
Scan End	1500 m/z	Set Collision Cell RF	600.0 Vpp	Set Divert Valve	Source

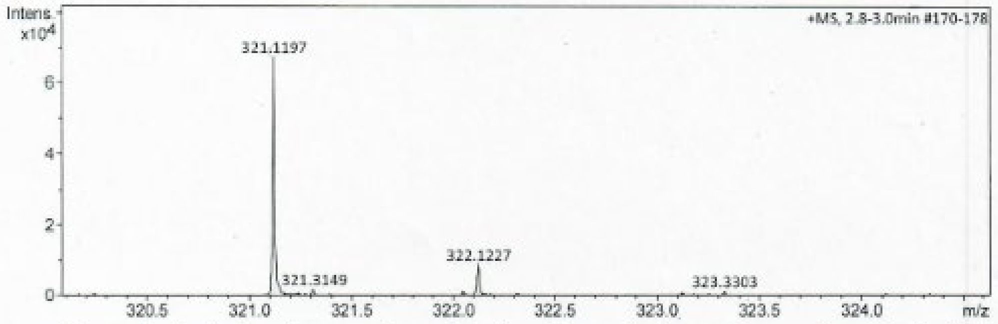

Meas. m/z	#	Ion Formula	m/z	err [ppm]	mSigma	# mSigma	Score	rdb	e⁻ Conf	N-Rule
321.1197	1	C12H16F7N2	321.1196	-0.2	7.6	1	100.00	2.5	even	ok

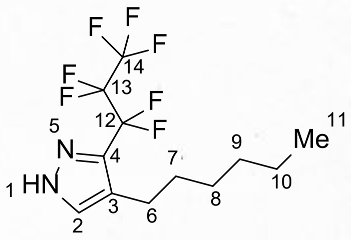

Mass Spectrum SmartFormula Report

Analysis Info

Analysis Name	D:\Data\Spektren 2022\CZE22HR000095.d	Acquisition Date	8/5/2022 8:51:07 AM
Method	tune_low_new.m	Operator	PT
Sample Name	Bunnemann LB_V41 (CH3OH)	Instrument	maXis 288882.20213
Comment			

Acquisition Parameter

Source Type	ESI	Ion Polarity	Positive	Set Nebulizer	0.3 Bar
Focus	Not active	Set Capillary	4000 V	Set Dry Heater	180 °C
Scan Begin	50 m/z	Set End Plate Offset	-500 V	Set Dry Gas	4.0 l/min
Scan End	1500 m/z	Set Collision Cell RF	600.0 Vpp	Set Divert Valve	Source

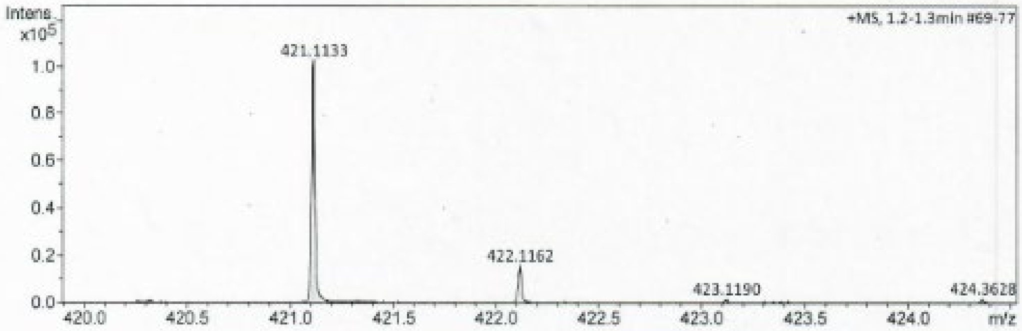

Meas. m/z	#	Ion Formula	m/z	err [ppm]	mSigma	# mSigma	Score	rdb	e⁻ Conf	N-Rule
421.1133	1	C14H16F11N2	421.1132	-0.0	5.2	1	100.00	2.5	even	ok

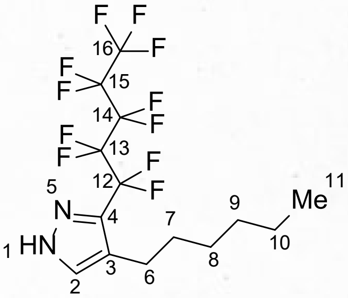

Mass Spectrum SmartFormula Report

Analysis Info

Analysis Name	D:\Data\Spektren 2022\CZE22HR000093.d	Acquisition Date	8/4/2022 4:43:07 PM
Method	tune_low_new.m	Operator	PT
Sample Name	Bunnemann LB_V40 (CH3OH)	Instrument	maXis 288882.20213
Comment			

Acquisition Parameter

Source Type	ESI	Ion Polarity	Positive	Set Nebulizer	0.3 Bar
Focus	Not active	Set Capillary	4000 V	Set Dry Heater	180 °C
Scan Begin	50 m/z	Set End Plate Offset	-500 V	Set Dry Gas	4.0 l/min
Scan End	1500 m/z	Set Collision Cell RF	600.0 Vpp	Set Divert Valve	Source

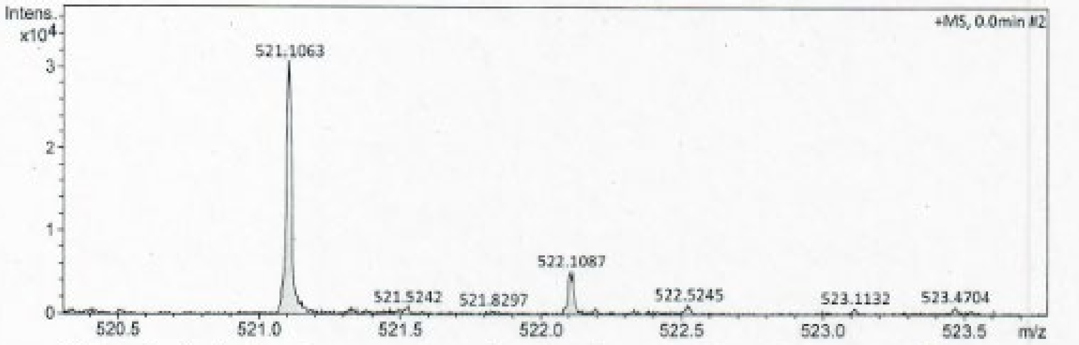

Meas. m/z	#	Ion Formula	m/z	err [ppm]	mSigma	# mSigma	Score	rdb	e⁻ Conf	N-Rule
521.1063	1	C16H16F15N2	521.1068	1.1	10.0	1	100.00	2.5	even	ok

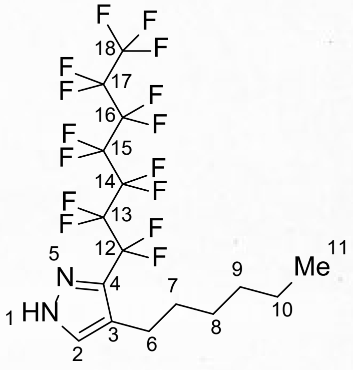

Mass Spectrum SmartFormula Report

Analysis Info

Analysis Name	D:\Data\Spektren 2023\CZE23HR000065.d	Acquisition Date	5/26/2023 12:45:51 PM
Method	tune_low_new.m	Operator	PT
Sample Name	Bunnemann LB_Py_V67 (CH3OH)	Instrument	maXis 288882.20213
Comment			

Acquisition Parameter

Source Type	ESI	Ion Polarity	Positive	Set Nebulizer	0.3 Bar
Focus	Not active	Set Capillary	4000 V	Set Dry Heater	180 °C
Scan Begin	50 m/z	Set End Plate Offset	-500 V	Set Dry Gas	4.0 l/min
Scan End	1500 m/z	Set Collision Cell RF	600.0 Vpp	Set Divert Valve	Source

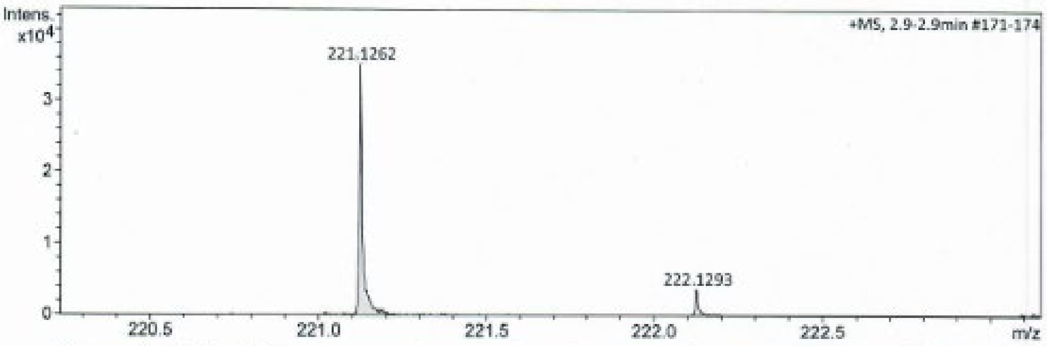

Meas. m/z	#	Ion Formula	m/z	err [ppm]	mSigma	# mSigma	Score	rdb	e⁻ Conf	N-Rule
221.1262	1	C10H16F3N2	221.1260	-1.0	9.1	1	100.00	2.5	even	ok

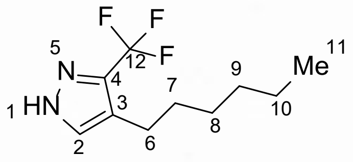

Mass Spectrum SmartFormula Report

Analysis Info

Acquisition Date 8/4/2022 3:48:36 PM

Analysis Name D:\Data\Spektren 2022\CZE22HR000090.d
Method tune_low_new.m
Sample Name Bunnemann LB_V33 (CH3OH)
Comment

Operator PT
Instrument maXis 288882.20213

Acquisition Parameter

Source Type	ESI	Ion Polarity	Positive	Set Nebulizer	0.3 Bar
Focus	Not active	Set Capillary	4000 V	Set Dry Heater	180 °C
Scan Begin	50 m/z	Set End Plate Offset	-500 V	Set Dry Gas	4.0 l/min
Scan End	1500 m/z	Set Collision Cell RF	600.0 Vpp	Set Divert Valve	Source

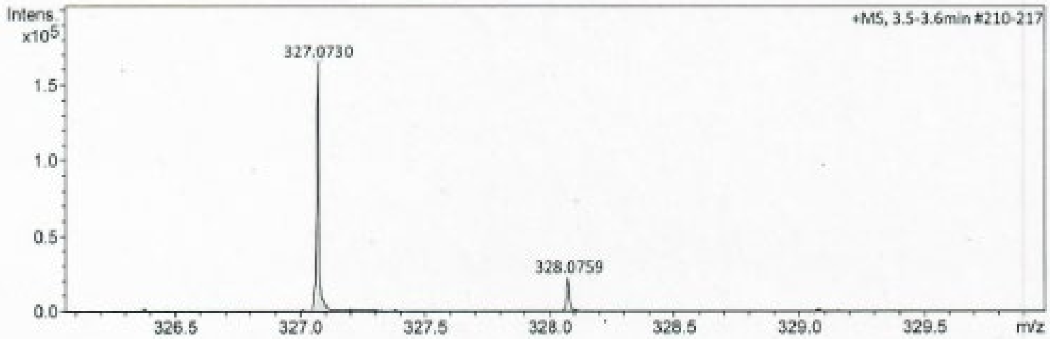

Meas. m/z	#	Ion Formula	m/z	err [ppm]	mSigma	# mSigma	Score	rdb	e⁻ Conf	N-Rule
327.0730	1	C13H10F7N2	327.0727	-0.9	8.6	1	100.00	6.5	even	ok

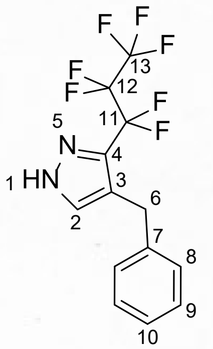

Bruker Compass DataAnalysis 4.2 printed: 8/4/2022 3:54:01 PM by: PT Page 1 of 1

Mass Spectrum SmartFormula Report

Analysis Info

Analysis Name	D:\Data\Spektren 2022\CZE22HR000091.d	Acquisition Date	8/4/2022 4:17:22 PM
Method	tune_low_new.m	Operator	PT
Sample Name	Bunnemann LB_V37 (CH3OH)	Instrument	maXis 288882.20213
Comment			

Acquisition Parameter

Source Type	ESI	Ion Polarity	Positive	Set Nebulizer	0.3 Bar
Focus	Not active	Set Capillary	4000 V	Set Dry Heater	180 °C
Scan Begin	50 m/z	Set End Plate Offset	-500 V	Set Dry Gas	4.0 l/min
Scan End	1500 m/z	Set Collision Cell RF	600.0 Vpp	Set Divert Valve	Source

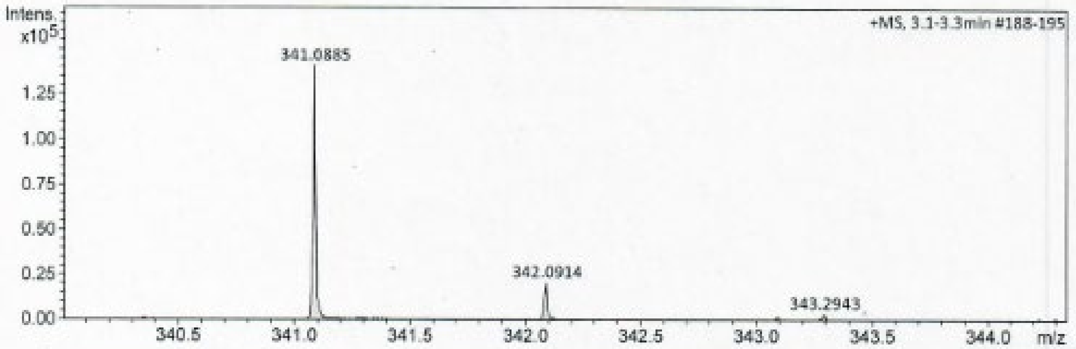

Meas. m/z	#	Ion Formula	m/z	err [ppm]	mSigma	# mSigma	Score	rdb	e⁻ Conf	N-Rule
341.0885	1	C14H12F7N2	341.0883	-0.6	8.2	1	100.00	6.5	even	ok

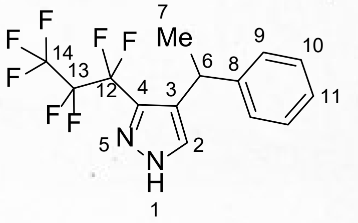

Mass Spectrum SmartFormula Report

Analysis Info

Analysis Name	D:\Data\Spektren 2022\CZE22HR000096.d	Acquisition Date	8/5/2022 12:29:52 PM
Method	tune_low_new.m	Operator	PT
Sample Name	Bunnemann LB_V45 (CH3OH)	Instrument	maXis 288882.20213
Comment			

Acquisition Parameter

Source Type	ESI	Ion Polarity	Positive	Set Nebulizer	0.3 Bar
Focus	Not active	Set Capillary	4000 V	Set Dry Heater	180 °C
Scan Begin	50 m/z	Set End Plate Offset	-500 V	Set Dry Gas	4.0 l/min
Scan End	1500 m/z	Set Collision Cell RF	600.0 Vpp	Set Divert Valve	Source

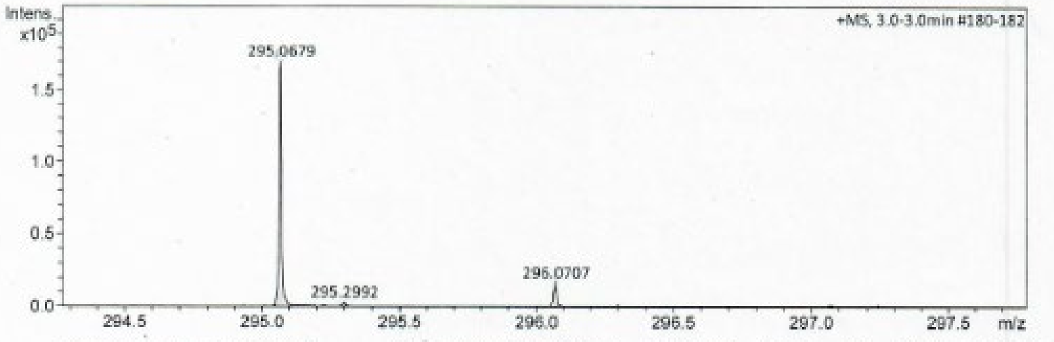

Meas. m/z	#	Ion Formula	m/z	err [ppm]	mSigma	# mSigma	Score	rdb	e⁻ Conf	N-Rule
295.0679	1	C9H10F7N2O	295.0676	-1.1	5.4	1	100.00	2.5	even	ok

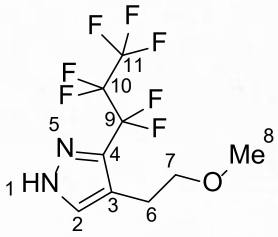

Mass Spectrum SmartFormula Report

Analysis Info

Analysis Name	D:\Data\Spektren 2022\CZE22HR000100.d	Acquisition Date	8/15/2022 12:09:26 PM
Method	tune_low_new.m		
Sample Name	Bunnemann LB_V47 (CH3OH)	Operator	PT
Comment		Instrument	maXis 288882.20213

Acquisition Parameter

Source Type	ESI	Ion Polarity	Positive	Set Nebulizer	0.3 Bar
Focus	Not active	Set Capillary	4000 V	Set Dry Heater	180 °C
Scan Begin	50 m/z	Set End Plate Offset	-500 V	Set Dry Gas	4.0 l/min
Scan End	1500 m/z	Set Collision Cell RF	600.0 Vpp	Set Divert Valve	Source

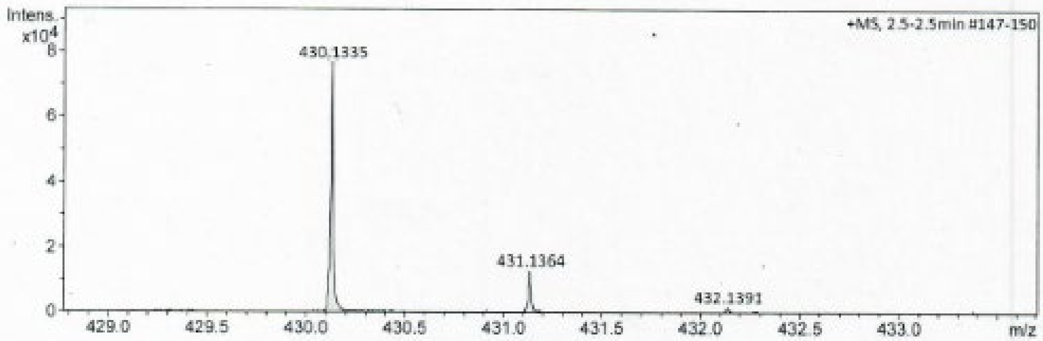

Meas. m/z	#	Ion Formula	m/z	err [ppm]	mSigma	# mSigma	Score	rdb	e⁻ Conf	N-Rule
430.1335	1	C15H20F7N3NaO2	430.1336	0.2	5.5	1	100.00	3.5	even	ok

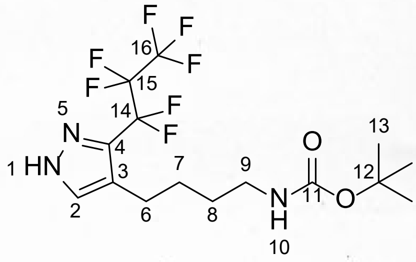

Mass Spectrum SmartFormula Report

Analysis Info

Analysis Name	D:\Data\Spektren 2022\CZE22HR000119.d	Acquisition Date	9/12/2022 8:34:49 AM
Method	tune_low_new.m	Operator	PT
Sample Name	Bunnemann LB_V52 (CH3OH)	Instrument	maXis 288882.20213
Comment			

Acquisition Parameter

Source Type	ESI	Ion Polarity	Positive	Set Nebulizer	0.3 Bar
Focus	Not active	Set Capillary	4000 V	Set Dry Heater	180 °C
Scan Begin	50 m/z	Set End Plate Offset	-500 V	Set Dry Gas	4.0 l/min
Scan End	1500 m/z	Set Collision Cell RF	600.0 Vpp	Set Divert Valve	Source

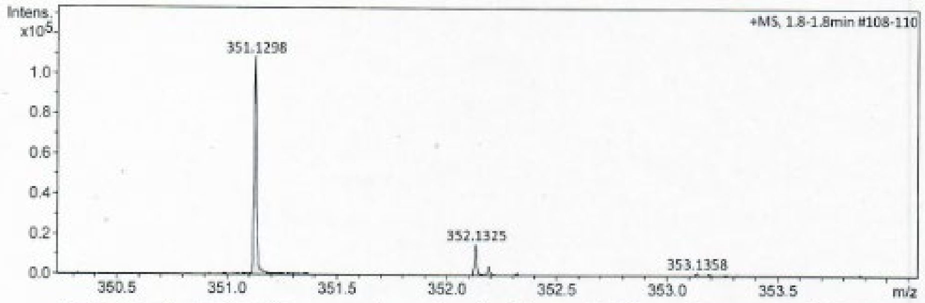

Meas. m/z	#	Ion Formula	m/z	err [ppm]	mSigma	# mSigma	Score	rdb	e⁻ Conf	N-Rule
351.1298	1	C13H18F7N2O	351.1302	1.2	6.0	1	100.00	2.5	even	ok

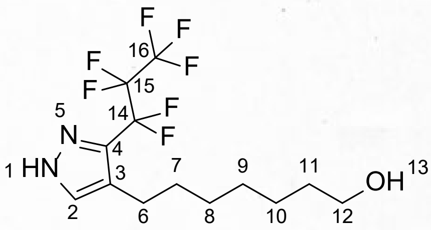

Mass Spectrum SmartFormula Report

Analysis Info

Analysis Name	D:\Data\Spektren 2023\CZE23HR000084.d	Acquisition Date	6/21/2023 8:54:58 AM
Method	tune_low_new.m	Operator	PT
Sample Name	Bunnemann LB_Py_V69_Frak1 (CH3OH)	Instrument	maXis 288882.20213
Comment			

Acquisition Parameter

Source Type	ESI	Ion Polarity	Positive	Set Nebulizer	0.3 Bar
Focus	Not active	Set Capillary	4000 V	Set Dry Heater	180 °C
Scan Begin	50 m/z	Set End Plate Offset	-500 V	Set Dry Gas	4.0 l/min
Scan End	1500 m/z	Set Collision Cell RF	600.0 Vpp	Set Divert Valve	Source

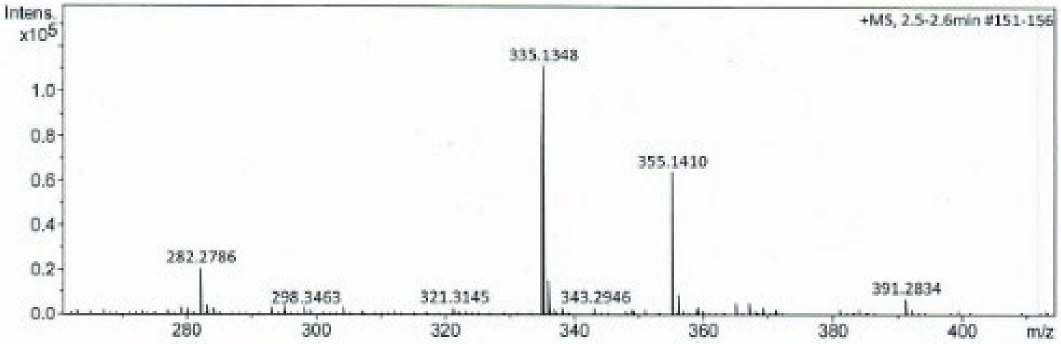

Meas. m/z	#	Ion Formula	m/z	err [ppm]	mSigma	# mSigma	Score	rdb	e⁻ Conf	N-Rule
335.1348	1	C13H18F7N2	335.1353	1.3	8.3	1	100.00	2.5	even	ok

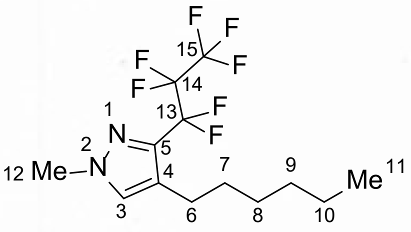

Mass Spectrum SmartFormula Report

Analysis Info

Analysis Name	D:\Data\Spektren 2023\CZE23HR000085.d	Acquisition Date	6/21/2023 9:07:48 AM
Method	tune_low_new.m	Operator	PT
Sample Name	Bunnemann LB_Py_V69_Frak2 (CH3OH)	Instrument	maXis 288882.20213
Comment			

Acquisition Parameter

Source Type	ESI	Ion Polarity	Positive	Set Nebulizer	0.3 Bar
Focus	Not active	Set Capillary	4000 V	Set Dry Heater	180 °C
Scan Begin	50 m/z	Set End Plate Offset	-500 V	Set Dry Gas	4.0 l/min
Scan End	1500 m/z	Set Collision Cell RF	600.0 Vpp	Set Divert Valve	Source

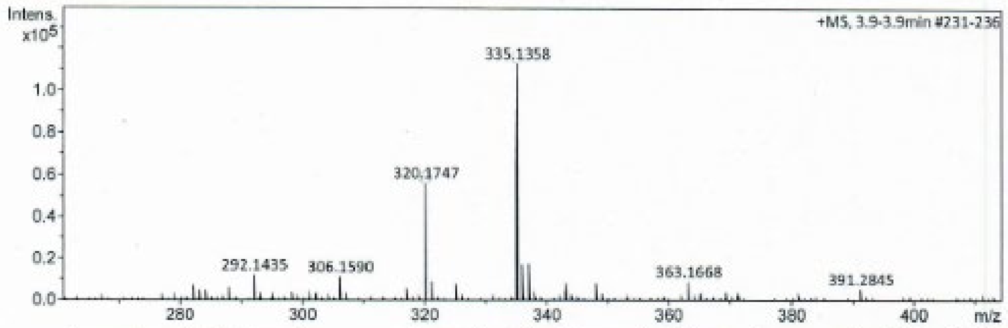

Meas. m/z	#	Ion Formula	m/z	err [ppm]	mSigma	# mSigma	Score	rdb	e⁻ Conf	N-Rule
320.1747	1	C12H23FN5O4	320.1729	-5.8	5.7	1	41.23	3.5	even	ok
	2	C13H19FN9	320.1742	-1.7	8.0	2	89.43	8.5	even	ok
	3	C15H22F4N3	320.1744	-0.9	8.1	3	100.00	4.5	even	ok
	4	C12H23F5N3O	320.1756	2.7	11.1	4	71.84	0.5	even	ok
	5	C10H20F2N9O	320.1753	1.9	11.1	5	81.43	4.5	even	ok
	6	C9H24F2N5O5	320.1740	-2.3	24.1	6	58.51	-0.5	even	ok
	7	C20H22N3O	320.1757	3.2	39.7	7	34.02	11.5	even	ok
335.1358	1	C13H18F7N2	335.1353	-1.4	83.8	1	100.00	2.5	even	ok

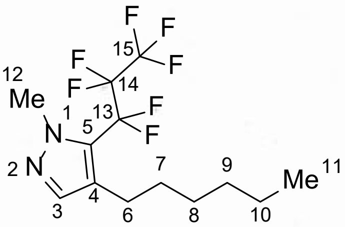

Supplement: Supplementary file 1 [file molecules-29-05034-s001.zip › molecules-3262835-supplementary.pdf]
